# Nickel-catalyzed cyclization of alkyne-nitriles with organoboronic acids involving *anti*-carbometalation of alkynes†
†Electronic supplementary information (ESI) available. CCDC 1440646 and 1440647. For ESI and crystallographic data in CIF or other electronic format see DOI: 10.1039/c6sc01191h


**DOI:** 10.1039/c6sc01191h

**Published:** 2016-05-19

**Authors:** Xingjie Zhang, Xin Xie, Yuanhong Liu

**Affiliations:** a State Key Laboratory of Organometallic Chemistry , Shanghai Institute of Organic Chemistry , Chinese Academy of Sciences , 345 Lingling Road , Shanghai 200032 , China . Email: yhliu@sioc.ac.cn

## Abstract

A nickel-catalyzed regioselective addition/cyclization of *o*-(cyano)phenyl propargyl ethers with arylboronic acids has been developed, which provides an efficient protocol for the synthesis of highly functionalized 1-naphthylamines with wide structural diversity. The reaction is characterized by a regioselective and *anti*-addition of the arylboronic acids to the alkyne and subsequent facile nucleophilic addition of the resulting alkenylmetal to the tethered cyano group. Mechanistic studies reveal that a Ni(i) species might be involved in the catalytic process.

## Abstract 2

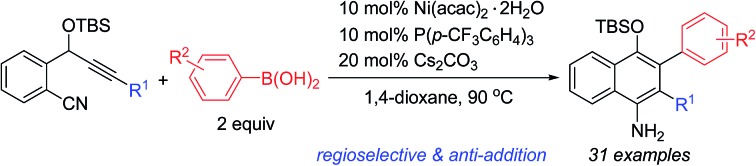
Nickel-catalyzed regioselective addition/cyclization of *o*-(cyano)phenyl propargyl ethers with arylboronic acids provides an efficient protocol for the synthesis of functionalized 1-naphthylamines.

## Introduction

**Scheme 1:**
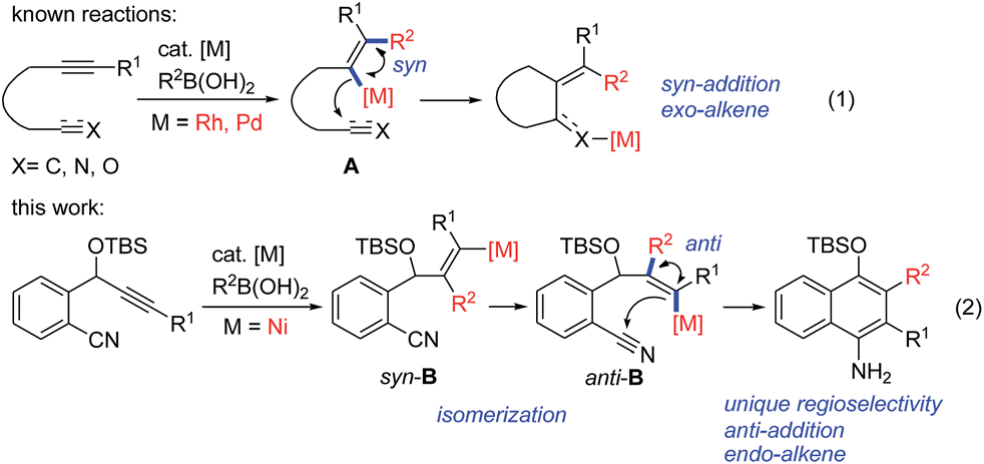
Metal-catalyzed cascade addition/cyclization reactions.

Transition-metal-catalyzed cascade reactions consisting of multiple carbometalation steps have attracted considerable attention in organic synthesis since these processes enable the rapid assembly of complex structures in an efficient, atom-economical and green manner.^1^ Among these reactions, organoboron compounds are one of the most widely used reagents, not only due to their chemical stability and ready availability, but also because they can undergo a series of addition reactions to unsaturated compounds such as alkynes, dienes, enones, aldehydes/ketones, nitriles and isocyanates *etc.* in the presence of a transition metal catalyst, especially Rh, Pd or Ni complexes.^2^ The development of cascade reactions by combining different types of these elemental reactions is undoubtedly important and attractive. In this regard, cascade reactions involving the addition of organoboron compounds to alkynes as the initial step have been realized mainly through Rh- 1b,c,^3^ or Pd-catalysis,^4^ as reported by Murakami, Hayashi, Lu and other groups. These catalytic reactions generally proceed by *syn*-1,2-addition of the organometal species generated through transmetalation between the organoboron and the metal complex across the carbon–carbon triple bond, followed by nucleophilic attack of the resulting alkenylmetal on the remaining electrophile (Scheme 1, eqn (1)). So far most of the reported reactions proceed *via* formation of regioisomer A in which the R^2^ group of R^2^B(OH)_2_ locates on a carbon adjacent to the alkyne terminus R^1^, leading to an *exo*-alkene upon cyclization^3^,^4^ (Scheme 1, eqn (1)). Cyclizations involving the regioselective formation of the alkenylmetal with a metal α-to the R^1^ substituent such as *syn*-B are quite rare^5^ (Scheme 1, eqn (2)), possibly because the subsequent cyclization process will involve a highly strained transition state. Thus, the development of new cyclization systems with controlled regiochemistry towards B is highly challenging. During our studies on nickel-catalyzed reactions, we found that such a transformation could be achieved by the addition of organoboron compounds to benzene-tethered alkyne-nitriles utilizing nickel as the catalyst, possibly through the isomerization of *syn*-B to *anti*-B. Herein, we report the first example of a nickel-catalyzed carboarylative cyclization of alkyne-nitriles with organoboronic acids involving regioselective and *anti*-carbonickelation of alkynes, which provides an efficient protocol for the synthesis of highly functionalized 1-naphthylamines. In addition, mechanistic studies revealed that Ni(i) species^6^ rather than Ni(ii) species were involved as the key intermediates, which has not been reported in Ni-catalyzed boron addition reactions.

## Results and discussion

We chose the nickel-catalyzed reaction of *o*-(cyano)phenyl propargyl ether^7^**1a** and phenylboronic acid as a model reaction for the optimization of the reaction conditions. Initially, we examined the reactions in the presence of Ni(COD)_2_ and various phosphine ligands such as PPh_3_ in 1,4-dioxane at 90 °C. However, only a trace of the desired cyclization product was observed, along with some byproducts (Table 1, entry 1). Replacing Ni(COD)_2_ with a Ni(ii) complex, Ni(acac)_2_·2H_2_O (acac = acetylacetonate), afforded the cyclized product 4-OTBS-substituted 1-naphthylamine **2a**, albeit in only 17–18% yields (entries 2–3). To our delight, the addition of 10 mol% of ^*t*^BuOK as a base improved the yield of **2a** dramatically to 66% within a short reaction time (entry 4). The results suggest that a base is necessary for this reaction, possibly for promoting the transmetalation step by formation of a borate^8^ with the organoboronic acid. The structure of **2a** also revealed that arylation in the initial step occurred regioselectively on the alkyne carbon that is closer to the OTBS group. Subsequently, the effects of bases, phosphine ligands and solvents were evaluated. Of the various bases, Cs_2_CO_3_ gave the best result (73%, entry 5). Increasing the catalytic loading of Cs_2_CO_3_ to 20 mol% had little effect on the yield of **2a** (entry 8). However, when a stoichiometric amount of Cs_2_CO_3_ was used, the yield was reduced rapidly (entry 9). It was remarkable that, unlike the use of more than one equivalent of base in most of the transition metal-catalyzed reactions involving organoborons, here only catalytic amounts of base were needed. Increasing the catalyst loading did not improve the yield of the product (entry 10). Triarylphosphine ligands and the N-heterocyclic carbene ligand IPr (IPr = 1,3-bis(2,6-diisopropylphenyl)imidazole-2-ylidene) were also effective, while ligands such as PPh_2_Me and PCy_3_ were less efficient (entries 11–17). Changing the solvent to THF or toluene afforded **2a** in satisfactory yields of 64–68% (entries 18–19). Addition of one equivalent of H_2_O as a promoter or proton source did not afford a better result (entry 20). Ni(acac)_2_ also catalyzed the reaction efficiently (entry 21). When Ni(COD)_2_ was used as the catalyst, only trace amounts of **2a** were obtained (entry 22). Without the phosphine ligand, the reaction also proceeded to afford **2a** in 65% yield, albeit with a longer reaction time (entry 23). Without a nickel catalyst, no reaction occurred (entry 24). On the basis of the above optimization studies, the reaction conditions shown in Table 1, entry 5 were chosen as the best conditions.

**Table 1:** Optimization studies for the formation of 1-naphthylamine **2a**

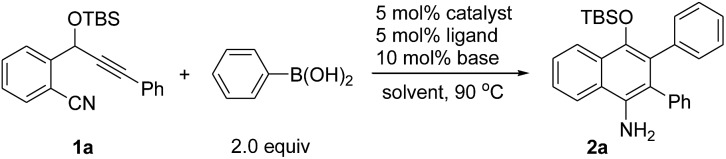						
Entry	Catalyst	Ligand	Base	Solvent	Time [h]	Yield^*a*^ [%]
1	Ni(COD)_2_	PPh_3_^*b*^	—	1,4-Dioxane	3	Trace
2	Ni(acac)_2_·2H_2_O	PPh_3_	—	1,4-Dioxane	24	18
3	Ni(acac)_2_·2H_2_O	P(*p*-CF_3_C_6_H_4_)_3_	—	1,4-Dioxane	24	17
4	Ni(acac)_2_·2H_2_O	P(*p*-CF_3_C_6_H_4_)_3_	^*t*^BuOK	1,4-Dioxane	4	66
5	Ni(acac)_2_·2H_2_O	P(*p*-CF_3_C_6_H_4_)_3_	Cs_2_CO_3_	1,4-Dioxane	3	73
6	Ni(acac)_2_·2H_2_O	P(*p*-CF_3_C_6_H_4_)_3_	CsF	1,4-Dioxane	6	67
7	Ni(acac)_2_·2H_2_O	P(*p*-CF_3_C_6_H_4_)_3_	K_2_CO_3_	1,4-Dioxane	10	31
8	Ni(acac)_2_·2H_2_O	P(*p*-CF_3_C_6_H_4_)_3_	Cs_2_CO_3_^*c*^	1,4-Dioxane	3	68
9	Ni(acac)_2_·2H_2_O	P(*p*-CF_3_C_6_H_4_)_3_	Cs_2_CO_3_^*d*^	1,4-Dioxane	10	8 (59)
10^*e*^	Ni(acac)_2_·2H_2_O	P(*p*-CF_3_C_6_H_4_)_3_	Cs_2_CO_3_	1,4-Dioxane	3	74
11	Ni(acac)_2_·2H_2_O	PPh_3_	Cs_2_CO_3_	1,4-Dioxane	5	69
12	Ni(acac)_2_·2H_2_O	P(*p*-MeC_6_H_4_)_3_	Cs_2_CO_3_	1,4-Dioxane	4	68
13	Ni(acac)_2_·2H_2_O	P(C_6_F_5_)_3_	Cs_2_CO_3_	1,4-Dioxane	5	63
14	Ni(acac)_2_·2H_2_O	PPh_2_Me	Cs_2_CO_3_	1,4-Dioxane	7	45
15	Ni(acac)_2_·2H_2_O	PCy_3_	Cs_2_CO_3_	1,4-Dioxane	17	55
16	Ni(acac)_2_·2H_2_O	IPr	Cs_2_CO_3_	1,4-Dioxane	6	64
17	Ni(acac)_2_·2H_2_O	IPr	^*t*^BuOK	1,4-Dioxane	7	62
18	Ni(acac)_2_·2H_2_O	P(*p*-CF_3_C_6_H_4_)_3_	Cs_2_CO_3_	THF	8	64
19	Ni(acac)_2_·2H_2_O	P(*p*-CF_3_C_6_H_4_)_3_	Cs_2_CO_3_	Toluene	3	68
20^*f*^	Ni(acac)_2_·2H_2_O	P(*p*-CF_3_C_6_H_4_)_3_	Cs_2_CO_3_	1,4-Dioxane	6	57
21	Ni(acac)_2_	P(*p*-CF_3_C_6_H_4_)_3_	Cs_2_CO_3_	1,4-Dioxane	3	72
22	Ni(COD)_2_	P(*p*-CF_3_C_6_H_4_)_3_^*b*^	Cs_2_CO_3_	1,4-Dioxane	9	Trace
23	Ni(acac)_2_·2H_2_O	—	Cs_2_CO_3_	1,4-Dioxane	5	65
24	—	P(*p*-CF_3_C_6_H_4_)_3_	Cs_2_CO_3_	1,4-Dioxane	10	(99)

^*a*^Isolated yields. The yields of the recovered **1a** were shown in parentheses.

^*b*^10 mol% of the ligand was used.

^*c*^20 mol% of Cs_2_CO_3_ was used.

^*d*^1.0 equiv. of Cs_2_CO_3_ was used.

^*e*^10 mol% Ni(acac)_2_·2H_2_O, 10 mol% P(*p*-CF_3_C_6_H_4_)_3_ and 20 mol% Cs_2_CO_3_ were used.

^*f*^One equiv. of H_2_O was added.

Next, we proceeded to investigate the scope of this new cascade addition/cyclization reaction catalyzed by Ni(acac)_2_·2H_2_O. The reactivity of various organoboronic acids was first examined using **1a** as a reaction partner (Table 2). During this process, we found that the 5 mol% catalyst loading was not effective in some cases and thus 10 mol% Ni(acac)_2_·2H_2_O, 10 mol% P(*p*-CF_3_C_6_H_4_)_3_ and 20 mol% Cs_2_CO_3_ were used in most of the cases to achieve better product yields. As shown in Table 2, a wide range of diversely substituted aryl- or heteroaryl-boronic acids were suitable for this reaction, leading to the desired 1-naphthylamines **2a–2s** in generally good to high yields. Arylboronic acids bearing electron-donating groups such as *p*-Me, *p*-^*t*^Bu and *p*-MeO or electron-withdrawing groups such as *p*-F, *p*-Cl, *p*-CF_3_, *p*-CN, *p*-CO_2_Et and *p*-Ac on the aryl ring underwent the cyclization smoothly to provide the corresponding 1-naphthylamines **2b–2j** in 64–77% yields, and these functional groups were well tolerated during the reaction. Of note is that the CN and Ac groups remained intact under the reaction conditions, and no nickel-catalyzed boron additions to these groups were observed. The results indicated that electron-poor or -rich aryl substituents on the arylboronic acid had little influence on the yields of products **2**. The sterically demanding *o*-MeO substituted arylboronic acid afforded **2k** with a longer reaction time and a lower yield of 47%, indicating that the reaction is markedly influenced by steric effects. Arylboronic acids with –MeO or –Cl substituents at the 3-, 3,4- or 3,5-positions of the phenyl ring, or with a biphenyl or 2-naphthyl ring transformed into products **2l–2m** and **2o–2r** efficiently in good yields. The use of 2-fluorophenylboronic acid gave **2n** in 41% yield. 2-Thienylboronic acid also participated in this cascade reaction, albeit with a lower yield of **2s**. However, when alkylboronic acids such as *n*-butylboronic acid were employed, no desired product was obtained.

**Table 2:** Scope of the reaction with respect to arylboronic acids^*a*^

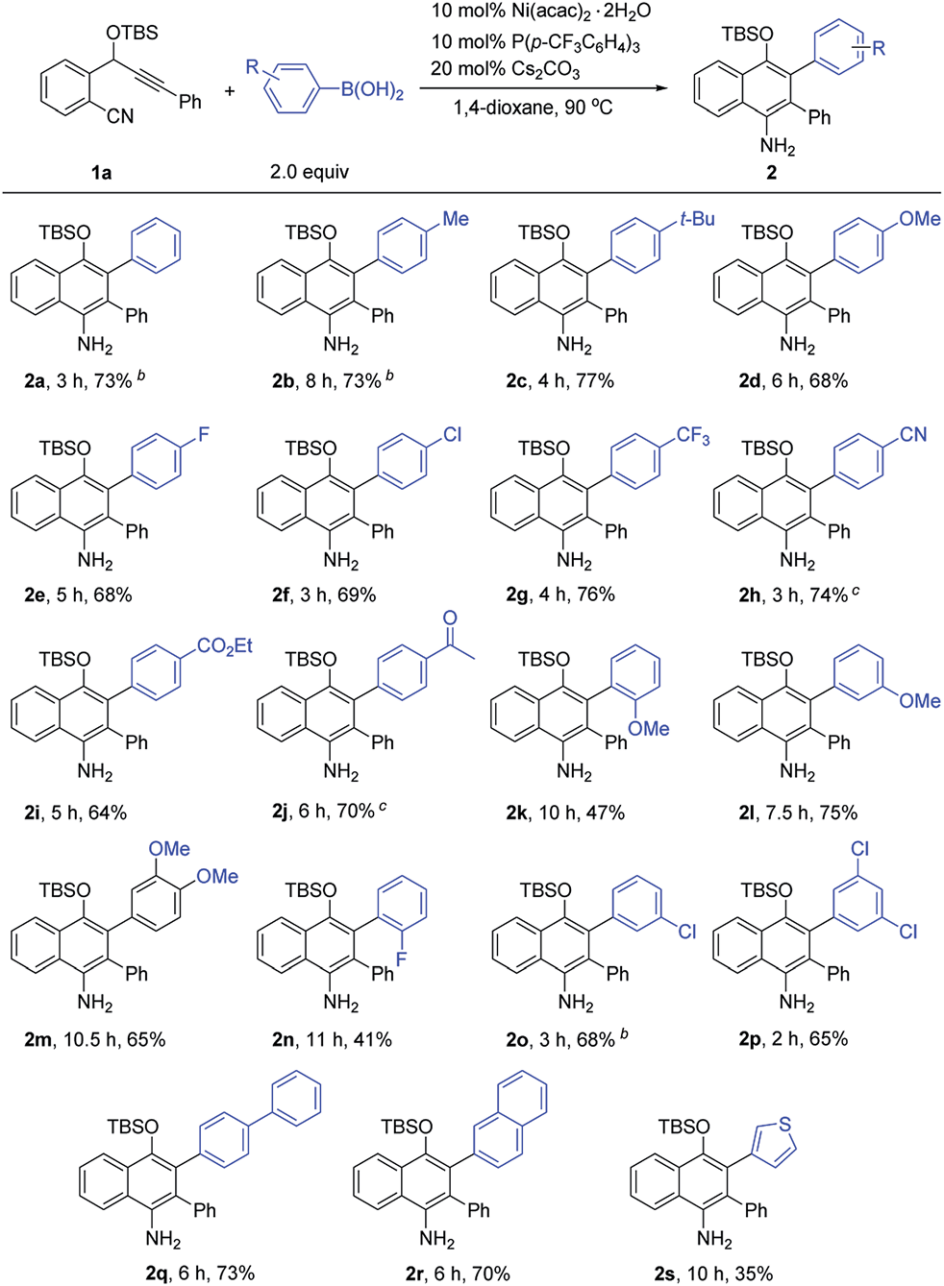

^*a*^The yields given are for the isolated products.

^*b*^5 mol% Ni(acac)_2_·2H_2_O, 5 mol% P(*p*-CF_3_C_6_H_4_)_3_ and 10 mol% Cs_2_CO_3_ were used.

^*c*^THF was used as the solvent.

The scope of *o*-(cyano)phenyl propargyl ethers was then examined (Table 3). A variety of electron-donating and -withdrawing groups on the aryl rings at the alkyne terminus were found to be compatible, such as *p*-Me, *p*-OMe, *p*-F, *p*-Cl, *p*-CF_3_ and *p*-CO_2_Me substituents, and the corresponding products **2t–2y** were formed in 61–76% yields. Interestingly, in contrast to the results of the reaction of **1a** with 2-thienylboronic acid, the presence of a 2-thienyl or 3-benzothienyl ring as the alkyne terminal did not have much influence on the reaction, and the corresponding products **2z** and **2za** were obtained in high yields of 80% and 74%, respectively. A 9*H*-fluorene substituent, which is a very useful unit in organofunctional materials, was also successfully incorporated into the product **2zb** in 70% yield. Alkenyl or alkyl-substituted alkynes, such as cyclohexenyl, cyclopropyl and propyl-substituted ones, however, afforded the desired products **2zc–2ze** in low yields of 23–35%. The structure of the 1-naphthylamine product was unambiguously confirmed by X-ray crystallographic analysis of **2o**.^9^ 1-Naphthylamines are important structural motifs found in a variety of biologically active substances. They also act as useful building blocks in synthetic chemistry and the dyestuffs industry. However, efficient methods for their synthesis are quite limited.^10^ Our reaction provides a convenient route to 1-naphthylamines.

**Table 3:** Scope of the *o*-(cyano)phenyl propargyl ethers^*a*^

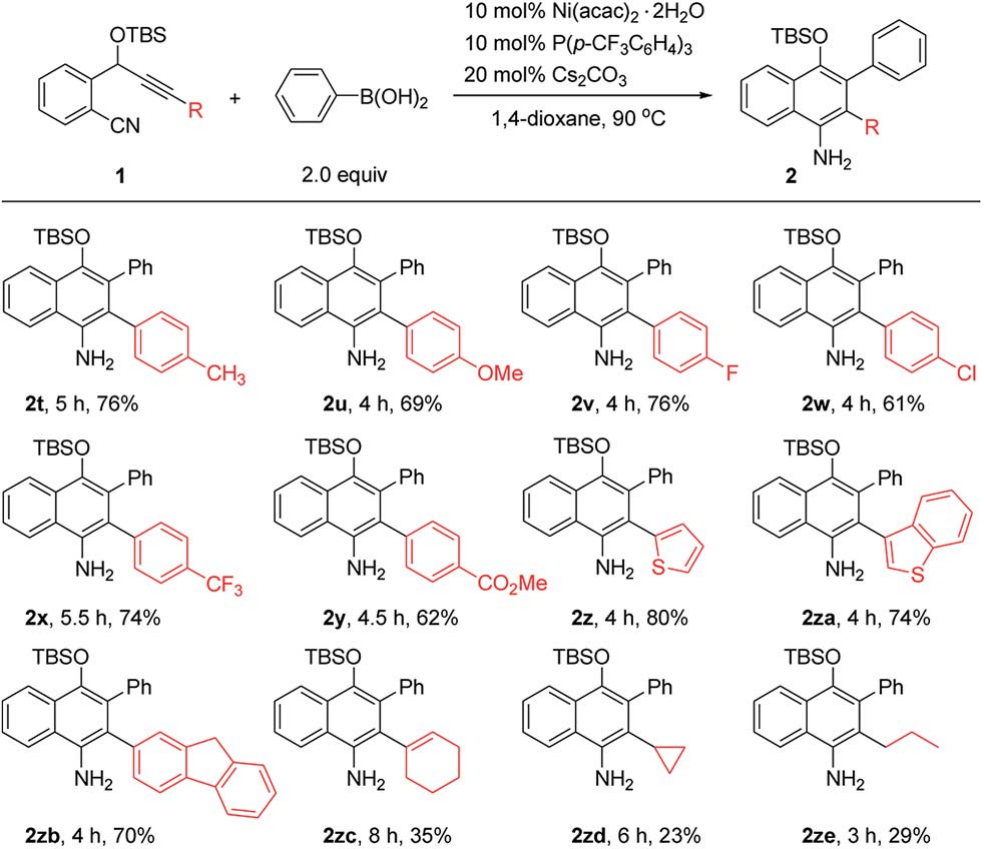

^*a*^The yields given are for the isolated products.

**Scheme 2:**
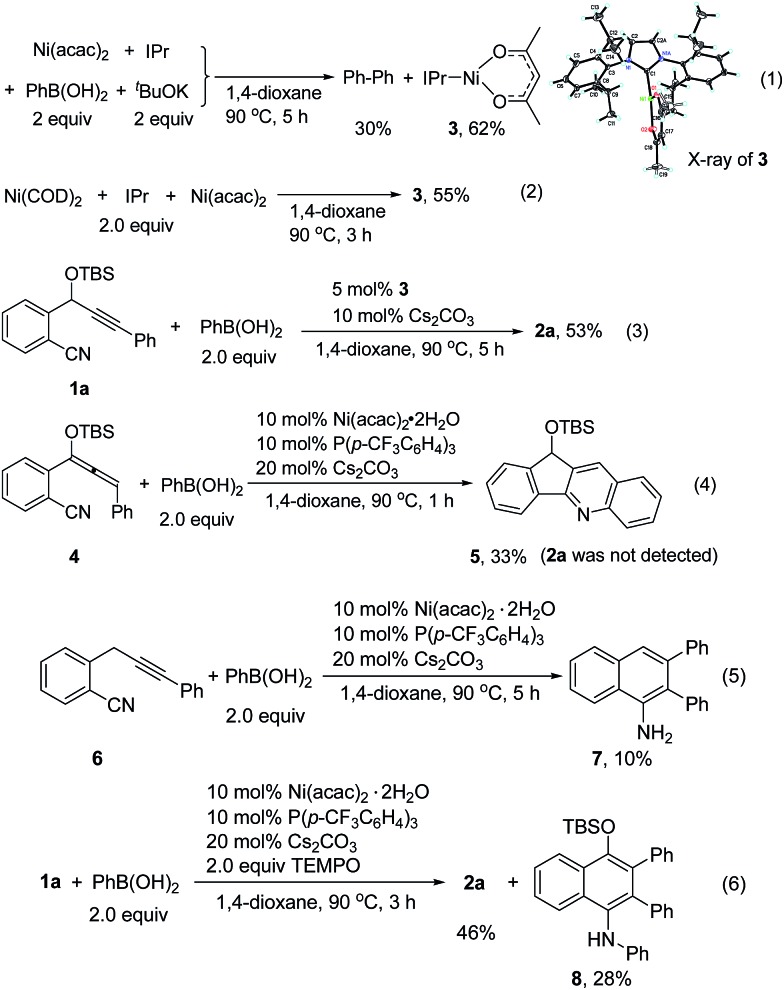
Mechanistic studies.

To understand the reaction mechanism, we tried to isolate the possible reaction intermediates. First, a stoichiometric reaction of Ni(acac)_2_ (1 equiv.), IPr (1 equiv.), PhB(OH)_2_ (2 equiv.) and ^*t*^BuOK (2 equiv.) was carried out (Scheme 2, eqn (1)). It was found that in addition to a biphenyl product, a red crystalline compound IPrNi(acac) **3** was isolated in 62% yield. Complex **3** is paramagnetic as indicated by the appearance of broad signals in the ^1^H NMR spectrum. The X-ray crystal analysis of **3** (ref. 9) clearly shows a rare, three-coordinate distorted T-shaped Ni(i) structure.6e,f When Cs_2_CO_3_ was used instead of ^*t*^BuOK, the same Ni(i) complex was also observed, albeit in a low yield of 14%. In this case, a large amount of precipitate could be observed. The precipitate was assumed to be the PhB(OH)_2_–Cs_2_CO_3_ adduct. Thus, the formation of this low-soluble adduct might prevent further reaction with the nickel complex. In fact, stirring a 1 : 1 mixture of PhB(OH)_2_ and Cs_2_CO_3_ in 1,4-dioxane at 90 °C resulted in the formation of a large amount of white precipitate. This might also explain why the use of 1 equivalent of Cs_2_CO_3_ provided the cyclized product **2a** in a low yield (Table 1, entry 9). Complex **3** might be formed by the comproportionation of Ni(0) and Ni(ii) species.6c,d To confirm this point, the stoichiometric reaction of Ni(COD)_2_ with Ni(acac)_2_ in the presence of IPr was carried out. To our delight, the same complex **3** was formed in moderate yield (Scheme 2, eqn (2)). This Ni(i) complex **3** was found to catalyze the cyclization of **1a** with PhB(OH)_2_ to afford **2a** (Scheme 2, eqn (3)), implying that the Ni(i) species was likely involved in the catalytic process. Recently, a Ni(i) species was proposed to be relevant in Ni-catalyzed cross-coupling reactions.^6^ Our results demonstrate that Ni(i) may also be involved in the Ni-catalyzed addition reactions of organoboron reagents to alkynes. In addition, the reaction of allene **4** with PhB(OH)_2_ under Ni-catalyzed conditions afforded indeno[1,2-*b*]quinoline **5***via* base-promoted cyano-Schmittel cyclization,^7^ while the desired **2a** was not observed (Scheme 2, eqn (4)), indicating that the reaction does not involve the allene intermediate.

**Scheme 3:**
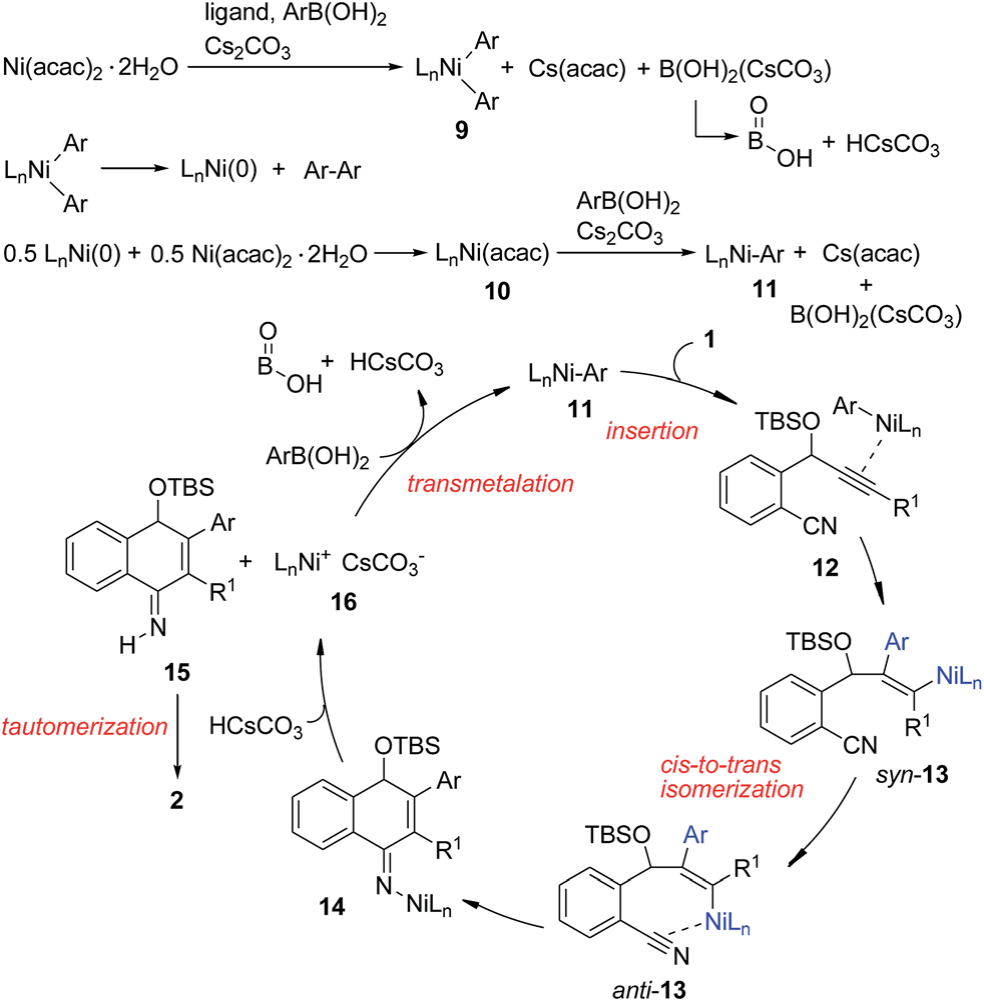
Possible reaction mechanism.

The origin of the regioselectivity in this reaction is not clear yet; it is possibly controlled by electronic factors in π–complex **12** (see Scheme 3).^11^ To understand the role of the OTBS group, alkyne-nitrile **6** without the OTBS group was synthesized. The Ni-catalyzed reaction of **6** with PhB(OH)_2_ afforded only 10% of the desired naphthylamine **7**, along with small amounts of an unidentified byproduct (Scheme 2, eqn (5)). The results indicate that the presence of the OTBS group is crucial for this reaction.

In addition, it was found that in the presence of a radical scavenger such as TEMPO, the desired **2a** and an *N*-arylated product **8** were formed in 74% combined yield (Scheme 2, eqn (6)). **8** was possibly formed by the Ni-catalyzed oxidative amination of the arylboronic acid with amine **2a**.^12^,^13^ The results indicate that the reaction was not inhibited by TEMPO, and this suggests that a radical species is not involved in this system.

Based on the above results, we propose the following reaction mechanism (Scheme 3). Initially, transmetalation of the arylboronic acid with the Ni(ii) complex, promoted by a base, provides diarylnickel(ii) species **9**, together with HOBO, HCsCO_3_ and Cs(acac).^14^**9** undergoes reductive elimination to form a Ni(0) species. The observation of biphenyl^15^ in the catalytic reaction of **1a** with PhB(OH)_2_ also indicates that Ni(ii) was reduced in the reaction process. This Ni(0) species comproportionates with Ni(ii) to afford Ni(i) complex **10**, which undergoes transmetalation with the arylboronic acid to give arylnickel(i) species **11**. Regioselective 1,2-addition of arylnickel(i) species **11** to the alkyne moiety in a *syn*-fashion takes place to give an alkenylnickel(i) intermediate *syn*-**13**. *cis*-to-*trans* isomerization of **13**,^16^ possibly through a carbene-like zwitterionic resonance species^17^ yields alkenylnickel(i) intermediate *anti*-**13** with a metal *trans*-to the Ar substituent. It was noted that most of the metal-catalyzed reactions of organoborons to alkynes gave the *syn*-addition product while few reactions produced the *anti*-addition product.2r,^17^ The regio- and stereochemistry for the addition process here are consistent with those observed for the cobalt(ii)-catalyzed hydroarylation of propargyl-alcohols or -carbamates with arylboronic acids.^18^ The cyano group may play a role in facilitating the *cis*–*trans* isomerization by stabilizing the metal species and directing the subsequent addition reaction. Nucleophilic attack of the alkenylmetal in *anti*-**13** to the cyano group forms a cyclized intermediate **14**. Subsequent protonation of **14** produces the N–H imine **15** and a nickel(i) species **16**. Tautomerization of **15** affords the observed product **2**. **16** undergoes transmetalation with ArB(OH)_2_ to regenerate the arylnickel(i) catalyst **11**.

## Conclusions

In summary, we have developed a nickel-catalyzed regioselective addition/cyclization of *o*-(cyano)phenyl propargyl ethers with arylboronic acids, which provides an efficient protocol for the synthesis of highly functionalized 1-naphthylamines with wide structural diversity. The reaction is characterized by a regioselective and *anti*-addition of the arylboronic acids to the alkyne and subsequent facile nucleophilic addition of the resulting alkenylmetal to the tethered cyano group. Mechanistic studies reveal that a Ni(i) species might be involved in the catalytic process. Further mechanistic studies and the extension to alkynes tethered with a wide variety of electrophiles are currently ongoing in our laboratory.

## Supplementary Material

Supplementary information

Crystal structure data
