# Supplementary material for: Nickel-catalyzed cyclization of alkyne-nitriles with organoboronic acids involving anti-carbometalation of alkynes
Source: Chem Sci. 2016 May 19;7(9):5815–20. doi: 10.1039/c6sc01191h (PMC6021782; doi:10.1039/c6sc01191h)

Electronic Supplementary Material (ESI) for Chemical Science.
This journal is © The Royal Society of Chemistry 2016

Supporting Information for

Nickel-Catalyzed Cyclization of Alkyne-Nitriles with Organoboronic Acids Involving *anti*-Carbometalation of Alkynes

Xingjie Zhang, Xin Xie and Yuanhong Liu*

State Key Laboratory of Organometallic Chemistry,

Shanghai Institute of Organic Chemistry, Chinese Academy of Sciences,

345 Lingling Lu, Shanghai 200032, People's Republic of China

Fax: (+86) 021-64166128, E-mail: yhliu@sioc.ac.cn

Contents: Page

General Methods	S1-S2
Synthesis and characterization of substrates **1**	S2-S11
Synthesis and characterization of 1-naphthylamine products **2**	S12-S34
Isolation of IPrNi(acac) **3**	S34-S37
Detection of biphenyl derivatives in catalytic reactions	S37-S38
Transformation of **1a** to **2a** using IPrNi(acac) **3** as the catalyst	S38-S39
Exclude the possibility of allene **4** as the intermediate	S39-S40
Synthesis of compounds **6** and **7**	S40-S41
Reaction of **1a** in the presence of TEMPO	S41-S43
Reaction of **2a** in the presence of TEMPO	S43
References	S43
X-ray crystal structure of compounds **2o** and **3**	S44
NMR spectra of all new compounds	S45-S92

General Methods. Unless noted, all reactions were carried out using standard Schlenk technique under an argon atmosphere or a dry box technique under a nitrogen atmosphere. Tetrahydrofuran and toluene were distilled from sodium and benzophenone, 1,4-dioxane

was distilled from sodium. EtMgBr (3.0 M solution in Et_2O) was purchased from J&K Chemical Company. All commercial reagents were used without further purification. ^1H and ^{13}C NMR spectra were recorded at room temperature in $CDCl_3$ (containing 0.03% TMS) solutions or C_6D_6 (containing 0.03% or 1% TMS) solutions on Varian or Agilent XL-400 MHz spectrometer. ^1H NMR spectra was recorded with tetramethylsilane (0.00 ppm) or solvent residual peak ($CDCl_3$: 7.26 ppm; C_6D_6: 7.16 ppm) as internal reference; ^{13}C NMR spectra was recorded with $CDCl_3$ (77.00 ppm) or C_6D_6 (128.06 ppm) as internal reference. High-resolution mass spectra was obtained by using Waters Micromass GCT Premier, Agilent Technologies 6224 TOF LC/MS or Thermo Fisher Scientific LTQ FT Ultra mass spectrometers. The IR spectra were measured on a Bruker Tensor 27 spectrometer. Single crystal X-ray diffraction data was collected at 273(2) K (for **2o**) and 130 K (for **3**) on a Bruker SMART diffractometer or a Bruker APEX-II diffractometer.

Synthesis of *o*-(cyano)phenyl propargyl ether 1.[1]

Typical procedure for the synthesis of 2-(1-((*tert*-Butyldimethylsilyl)oxy)-3-(*p*-tolyl)prop-2-yn-1-yl)benzonitrile (1b).

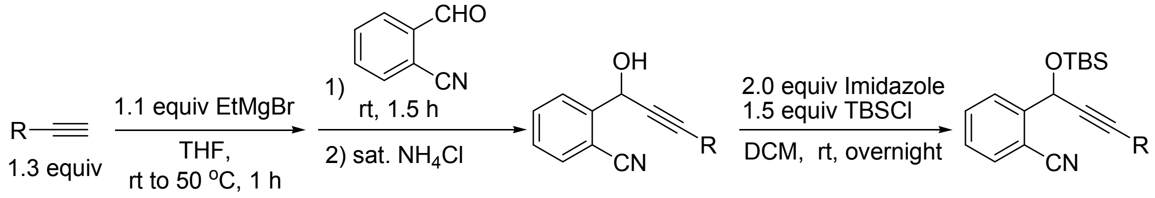

For **1b** R = *p*-tolyl

To a solution of 1-ethynyl-4-methylbenzene (755.0 mg, 6.5 mmol) in THF (15.0 mL) was added dropwise EtMgBr (1.8 mL, 3.0 M solution in Et_2O, 5.5 mmol) at room temperature under argon, then the mixture was stirred at 50 °C. After stirring for 1.0 h, 2-cyanobenzaldehyde (655.7 mg, 5.0 mmol) was added at room temperature, and then the reaction mixture was stirred at the same temperature until the reaction was completed as monitored by TLC (1.5 h). The resulting reaction mixture was quenched with saturated NH_4Cl solution, and extracted with ethyl acetate. The combined organic extracts were washed with water and brine, and dried over Na_2SO_4. The solvent was evaporated under the reduced pressure to afford the alcohol as an orange oil, which was used directly without further purification for the next step.

To a solution of the above crude alcohol in DCM (15.0 mL) were added imidazole (680.8 mg, 10.0 mmol) and TBSCl (1.13 g, 7.5 mmol). The reaction mixture was then stirred at room temperature overnight, and saturated NH_4Cl solution was added. Then the mixture was extracted with dichloromethane, washed with water and brine, dried over Na_2SO_4, and concentrated *in vacuo*. The residue was purified by column chromatography on silica gel (petroleum ether/ethyl acetate = 50/1) to afford **1b** in 76% overall yield (1.38 g) as a colorless oil.

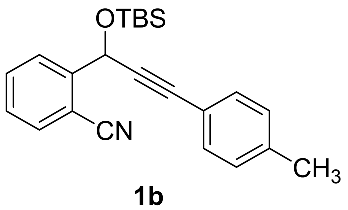

1b

2-(1-((*tert*-Butyldimethylsilyl)oxy)-3-(*p*-tolyl)prop-2-yn-1-yl)benzonitrile (1b). ^{1}H NMR(400 MHz, $CDCl_3$, Me_4Si): δ 0.25 (s, 3H), 0.30 (s, 3H), 0.98 (s, 9H), 2.35 (s, 3H), 6.00 (s, 1H), 7.12 (d, *J* = 8.0 Hz, 2H), 7.35 (d, *J* = 8.0 Hz, 2H), 7.40 (td, *J* = 7.6, 1.2 Hz, 1H), 7.62-7.68 (m, 2H), 7.89 (d, *J* = 7.6 Hz, 1H). ^{13}C NMR(100 MHz, $CDCl_3$, Me_4Si): δ -4.97, -4.41, 18.22, 21.42, 25.73, 63.45, 86.89, 87.47, 110.44, 117.19, 119.23, 127.21, 128.14, 128.97, 131.45, 132.90, 133.01, 138.69, 145.39. IR (film): 3678, 2954, 2930, 2887, 2857, 2225, 1907, 1601, 1510, 1468, 1333, 1254, 1206, 1180, 1107, 1067, 1006, 984, 940, 838, 816, 777, 762, 683, 623 cm^{-1}. HRMS (ESI) calcd for $C_{23}H_{31}N_2OSi$ $[M+NH_4]^+$: 379.2200, found 379.2202.

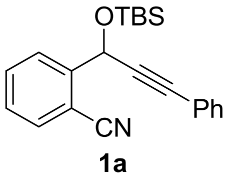

1a

2-(1-((*tert*-Butyldimethylsilyl)oxy)-3-phenylprop-2-yn-1-yl)benzonitrile (1a). (30 mmol scale. First step: To a solution of ethynylbenzene (36 mmol, 4.0 mL) in THF (100 mL) was added dropwise EtMgBr (3.0 M in diethyl ether, 33 mmol, 11 mL) at 0 ^{o}C, and the mixture was stirred at room temperature for 2.0 h, then 2-cyanobenzaldehyde (30 mmol, 3.93 g)

was added at 0 °C, and the mixture was stirred at room temperature for 2.5 h. Second step: DCM (100 mL), imidazole (60 mmol, 4.08 g), TBSCl (45 mmol, 6.78 g), stirred at room temperature overnight. Column chromatography on silica gel (eluent: petroleum ether: ethyl acetate =100:1) afforded the title product in 87% overall yield (9.03 g) as a light yellow oil. ^{1}H NMR(400 MHz, $CDCl_3$, Me_4Si): δ 0.26 (s, 3H), 0.31 (s, 3H), 0.99 (s, 9H), 6.01 (s, 1H), 7.30-7.34 (m, 3H), 7.41 (td, *J* = 7.8, 1.2 Hz, 1H), 7.45-7.47 (m, 2H), 7.63-7.68 (m, 2H), 7.90 (dd, *J* = 8.0, 0.4 Hz, 1H). ^{13}C NMR(100 MHz, $CDCl_3$, Me_4Si): δ -4.99, -4.44, 18.21, 25.71, 63.40, 86.67, 88.14, 110.41, 117.15, 122.26, 127.17, 128.17, 128.21, 128.53, 131.53, 132.91, 133.02, 145.25. The NMR data is in agreement with that previously reported.[1]

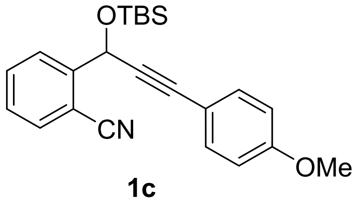

1c

2-(1-((*tert*-Butyldimethylsilyl)oxy)-3-(4-methoxyphenyl)prop-2-yn-1-yl)benzonitrile (1c). (5.0 mmol scale. First step: To a solution of 1-ethynyl-4-methoxybenzene (6.5 mmol, 859.0 mg) in THF (15.0 mL) was added dropwise EtMgBr (3.0 M in diethyl ether, 5.5 mmol, 1.8 mL) at room temperature, and the mixture was stirred at 50 °C for 1.0 h, then 2-cyanobenzaldehyde (5 mmol, 655.7 mg) was added at room temperature and stirred for 1.5 h. Second step: DCM (15.0 mL), imidazole (10 mmol, 680.8 mg), TBSCl (7.5 mmol, 1.13 g), stirred at room temperature for 2.5 h. Column chromatography on silica gel (eluent: petroleum ether: ethyl acetate = 50:1) afforded the title product in 77% overall yield (1.46 g) as a light yellow oil. ^{1}H NMR(400 MHz, $CDCl_3$, Me_4Si): δ 0.22 (s, 3H), 0.27 (s, 3H), 0.95 (s, 9H), 3.78 (s, 3H), 5.97 (s, 1H), 6.82 (d, *J* = 8.8 Hz, 2H), 7.36-7.40 (m, 3H), 7.60-7.65 (m, 2H), 7.87 (d, *J* = 8.0 Hz, 1H). ^{13}C NMR(100 MHz, $CDCl_3$, Me_4Si): δ -4.96, -4.42, 18.22, 25.74, 55.19, 63.51, 86.75, 86.83, 110.44, 113.86, 114.42, 117.19, 127.19, 128.09, 132.90, 132.98, 133.03, 145.52, 159.79. IR (film): 2954, 2931, 2890, 2857, 2224, 1605, 1570, 1509, 1466, 1446, 1290, 1249, 1175, 1107, 1066, 1033, 1008, 984, 833, 777, 684,

670, 623 cm^{-1}. HRMS (ESI) calcd for $C_{23}H_{31}N_2O_2Si$ [M+NH$_4$]$^+$: 395.2149, found 395.2150.

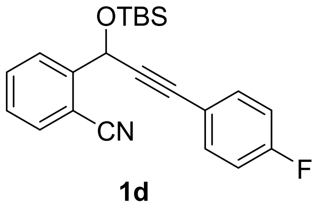

1d

2-(1-((*tert*-Butyldimethylsilyl)oxy)-3-(4-fluorophenyl)prop-2-yn-1-yl)benzonitrile (1d). (5.0 mmol scale. First step: To a solution of 1-ethynyl-4-fluorobenzene (6.5 mmol, 0.75 mL) in THF (15.0 mL) was added dropwise EtMgBr (3.0 M in diethyl ether, 5.5 mmol, 1.8 mL) at room temperature, and the mixture was stirred at 50 ^{o}C for 1.0 h, then 2-cyanobenzaldehyde (5 mmol, 655.7 mg) was added at room temperature and stirred for 2 h. Second step: DCM (15.0 mL), imidazole (10 mmol, 680.8 mg), TBSCl (7.5 mmol, 1.13 g), stirred at room temperature overnight. Column chromatography on silica gel (eluent: petroleum ether: ethyl acetate = 100:1) afforded the title product in 78% overall yield (1.43 g) as a yellow oil. ^{1}H NMR(400 MHz, $CDCl_3$, Me_4Si): δ 0.23 (s, 3H), 0.28 (s, 3H), 0.96 (s, 9H), 5.97 (s, 1H), 6.70 (t, J = 8.8 Hz, 2H), 7.39-7.44 (m, 3H), 7.62-7.68 (m, 2H), 7.86 (d, J = 7.6 Hz, 1H). ^{13}C NMR(100 MHz, $CDCl_3$, Me_4Si): δ -4.99, -4.47, 18.23, 25.71, 63.36, 85.57, 87.90 (d, J = 1.5 Hz), 110.37, 115.54 (d, J = 22.1 Hz), 117.14, 118.38 (d, J = 3.4 Hz), 127.08, 128.22, 133.02 (d, J = 9.9 Hz), 133.52 (d, J = 8.4 Hz), 145.24, 161.38, 163.87. IR (film): 3686, 3074, 2954, 2931, 2887, 2857, 2556, 2226, 2046, 1942, 1894, 1601, 1506, 1469, 1335, 1254, 1230, 1156, 1069, 1008, 984, 939, 835, 777, 762, 670, 623 cm^{-1}. HRMS (ESI) calcd for $C_{22}H_{28}FN_2OSi$ [M+NH$_4$]$^+$: 383.1949, found 383.1950.

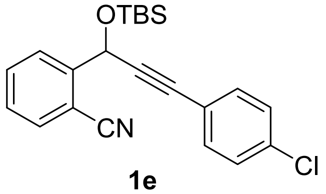

1e

2-(1-((*tert*-Butyldimethylsilyl)oxy)-3-(4-chlorophenyl)prop-2-yn-1-yl)benzonitrile (1e). (5.0 mmol scale. First step: To a solution of 1-chloro-4-ethynylbenzene (6 mmol, 819.5 mg)

in THF 20.0 mL was added dropwise EtMgBr (3.0 M in diethyl ether, 5.5 mmol, 1.8 mL) at room temperature, and the mixture was stirred at 40 °C for 1.0 h, then 2-cyanobenzaldehyde (5 mmol, 655.7 mg) was added at room temperature and stirred for 1.5 h. Second step: DCM (20.0 mL), imidazole (10 mmol, 680.8 mg), TBSCl (7.5 mmol, 1.13 g), stirred at room temperature for 3.5 h. Column chromatography on silica gel (eluent: petroleum ether: ethyl acetate = 100:1) afforded the title product in 68% overall yield (1.29 g) as a light yellow oil. ^{1}H NMR(400 MHz, $CDCl_3$, Me_4Si): δ 0.22 (s, 3H), 0.27 (s, 3H), 0.96 (s, 9H), 5.97 (s, 1H), 7.26 (d, *J* = 6.8 Hz, 2H), 7.36 (m, *J* = 6.6 Hz, 2H), 7.40 (td, *J* = 7.6, 0.8 Hz, 1H), 7.61-7.67 (m, 2H), 7.85 (d, *J* = 7.6 Hz, 1H). ^{13}C NMR(100 MHz, $CDCl_3$, Me_4Si): δ -5.00, -4.48, 18.21, 25.69, 63.34, 85.46, 89.14, 110.35, 117.10, 120.75, 127.07, 128.25, 128.56, 132.79, 132.96, 133.07, 134.61, 145.07. IR (film): 3679, 3073, 2954, 2931, 2888, 2857, 2711, 2364, 2226, 1941, 1903, 1596, 1488, 1468, 1396, 1330, 1254, 1206, 1069, 1012, 983, 831, 778, 759, 721, 674 cm^{-1}. HRMS (ESI) calcd for $C_{22}H_{28}ClN_2OSi$ $[M+NH_4]^+$: 399.1654, found 399.1655.

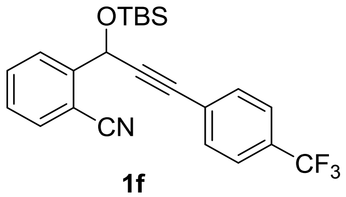

1f

2-(1-((*tert*-Butyldimethylsilyl)oxy)-3-(4-(trifluoromethyl)phenyl)prop-2-yn-1-yl)benzonitrile (1f). (5.0 mmol scale. First step: To a solution of 1-ethynyl-4-(trifluoromethyl)benzene (6.5 mmol, 1.11 g) in THF (15.0 mL) was added dropwise EtMgBr (3.0 M in diethyl ether, 6 mmol, 2.0 mL) at room temperature, and the mixture was stirred at 50 °C for 1.0 h, then 2-cyanobenzaldehyde (5 mmol, 655.7 mg) was added at room temperature and stirred for 3 h. Second step: DCM (15.0 mL), imidazole (10 mmol, 680.8 mg), TBSCl (7.5 mmol, 1.13 g), stirred at room temperature overnight. Column chromatography on silica gel (eluent: petroleum ether: diethyl ether = 100:1) afforded the title product in 78% overall yield (1.62 g) as a light yellow oil, which can be solidified upon standing. ^{1}H NMR(400 MHz, $CDCl_3$, Me_4Si): δ 0.24 (s, 3H), 0.30 (s, 3H), 0.98 (s, 9H), 6.01 (s, 1H), 7.42 (td, *J* = 7.4, 0.8 Hz, 1H), 7.56 (s, 4H), 7.64-7.69 (m, *2*H), 7.88 (d, *J*

= 8.0 Hz, 1H). ^{13}C NMR(100 MHz, $CDCl_3$, Me_4Si): δ -5.04, -4.55, 18.20, 25.66, 63.33, 85.11 (q, J = 0.7 Hz), 90.63, 110.39, 117.05, 123.76 (q, J = 274.7 Hz), 125.15 (q, J = 3.8 Hz), 126.09 (q, J = 1.6 Hz), 127.07, 128.36, 130.28 (q, J = 32.9 Hz), 131.84, 133.01, 133.12, 144.85. IR (film): 2955, 2933, 2890, 2859, 2225, 1614, 1468, 1404, 1320, 1282, 1254, 1162, 1119, 1048, 988, 885, 838, 778, 755, 721, 671, 633 cm^{-1}. HRMS (ESI) calcd for $C_{23}H_{28}F_3N_2OSi$ $[M+NH_4]^+$: 433.1918, found 433.1918.

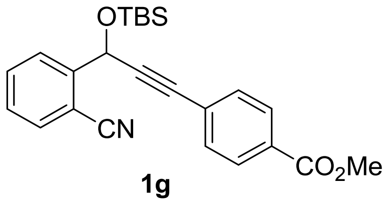

1g

Methyl 4-(3-((*tert*-butyldimethylsilyl)oxy)-3-(2-cyanophenyl)prop-1-yn-1-yl)-benzoate (1g). In this case, alkynyl lithium was used instead of alkynyl magnesium bromide. (4.0 mmol scale. First step: To a solution of methyl 4-ethynylbenzoate (5.2 mmol, 833.0 mg) in THF (15.0 mL) was added dropwise LDA (2.0 M in THF, 4.8 mmol, 2.4 mL) at -78 °C, and the mixture was stirred at the same temperature for 1.0 h, then 2-cyanobenzaldehyde (4 mmol, 524.5 mg) in THF (5 mL) was added at -78 °C and the mixture was stirred at room temperature for 3 h. Second step: DCM (15.0 mL), imidazole (8 mmol, 544.6 mg), TBSCl (6 mmol, 904.0 mg), stirred at room temperature overnight. Column chromatography on silica gel (eluent: petroleum ether: ethyl acetate = 50:1) afforded the title product in 16% overall yield (260.5 mg) as a yellow oil. ^{1}H NMR(400 MHz, $CDCl_3$, Me_4Si): δ 0.22 (s, 3H), 0.28 (s, 3H), 0.96 (s, 9H), 3.90 (s, 3H), 5.98 (s, 1H), 7.42 (td, J = 7.8, 1.2 Hz, 1H), 7.49 (d, J = 8.4 Hz, 2H), 7.63-7.68 (m, 2H), 7.86 (d, J = 8.0 Hz, 1H), 7.97 (d, J = 8.4 Hz, 2H). ^{13}C NMR(100 MHz, $CDCl_3$, Me_4Si): δ -4.99, -4.47, 18.24, 25.71, 52.21, 63.37, 85.75, 91.10, 110.38, 117.09, 126.94, 127.13, 128.34, 129.40, 129.84, 131.52, 133.01, 133.15, 144.95, 166.40. IR (film): 3681, 3554, 3431, 2953, 2888, 2857, 2365, 2226, 1936, 1724, 1605, 1468, 1437, 1405, 1275, 1177, 1107, 1069, 1016, 983, 836, 764, 719, 694, 673 cm^{-1}. HRMS (ESI) calcd for $C_{24}H_{31}N_2O_3Si$ $[M+NH_4]^+$: 423.2098, found 423.2098.

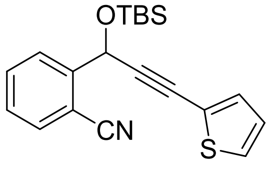

1h

2-(1-((*tert*-Butyldimethylsilyl)oxy)-3-(thiophen-2-yl)prop-2-yn-1-yl)benzonitrile (1h). (5.0 mmol scale. First step: To a solution of 2-ethynylthiophene (6.5 mmol, 0.65 mL) in THF (20.0 mL) was added dropwise EtMgBr (3.0 M in diethyl ether, 5.5 mmol, 1.8 mL) at room temperature, and the mixture was stirred at 50 °C for 1.0 h, then 2-cyanobenzaldehyde (5 mmol, 655.7 mg) was added at room temperature and stirred for 2 h. Second step: DCM (20.0 mL), imidazole (10 mmol, 680.8 mg), TBSCl (7.5 mol, 1.13 g), stirred at room temperature overnight. Column chromatography on silica gel (eluent: petroleum ether: ethyl acetate = 100:1) afforded the title product in 78% overall yield (1.37 g) as a brown oil. ^{1}H NMR(400 MHz, $CDCl_3$, Me_4Si): δ 0.21 (s, 3H), 0.27 (s, 3H), 0.95 (s, 9H), 5.99 (s, 1H), 6.95 (dd, *J* = 5.2, 3.6 Hz, 1H), 7.20-7.25 (m, 2H), 7.40 (td, *J* = 7.4, 0.8 Hz, 1H), 7.61-7.66 (m, 2H), 7.85 (d, *J* = 8.0 Hz, 1H). ^{13}C NMR(100 MHz, $CDCl_3$, Me_4Si): δ -4.98, -4.47, 18.22, 25.72, 63.52, 80.17, 91.86, 110.42, 117.08, 122.11, 126.91, 127.30, 127.51, 128.30, 132.40, 132.91, 133.10, 144.92. IR (film): 2954, 2930, 2888, 2857, 2225, 1599, 1468, 1424, 1359, 1316, 1255, 1208, 1187, 1107, 1064, 1006, 943, 836, 777, 702, 628 cm^{-1}. HRMS (ESI) calcd for $C_{20}H_{27}N_2OSSi$ $[M+NH_4]^+$: 371.1608, found 371.1608.

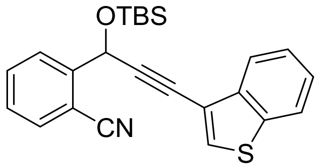

1i

2-(3-(Benzo[*b*]thiophen-3-yl)-1-((*tert*-butyldimethylsilyl)oxy)prop-2-yn-1-yl)benzonitrile (1i). (5.0 mmol scale. First step: To a solution of 3-ethynylbenzo[*b*]thiophene (7.8 mmol, 1.24 g), THF (15.0 mL) was added dropwise EtMgBr (3.0 M in diethyl ether, 6 mmol, 2.0 mL) was added at room temperature, and the mixture was stirred at 50 °C for 1.0 h, then 2-cyanobenzaldehyde (5 mmol, 655.7 mg) was added at room temperature and stirred for 2 h. Second step: DCM (15.0 mL), imidazole (10 mmol, 680.8 mg), TBSCl (7.5 mmol, 1.13 g), stirred at room temperature overnight.

Column chromatography on silica gel (eluent: petroleum ether: ethyl acetate = 100:1) afforded the title product in 78% overall yield (1.57 g) as a yellow oil. ^{1}H NMR(400 MHz, $CDCl_3$, Me_4Si): δ 0.27 (s, 3H), 0.33 (s, 3H), 1.00 (s, 9H), 6.10 (s, 1H), 7.37-7.48 (m, 3H), 7.64 (s, 1H), 7.65-7.71 (m, 2H), 7.84 (d, J = 8.0 Hz, 1H), 7.95 (t, J = 6.8 Hz, 2H). ^{13}C NMR(100 MHz, $CDCl_3$, Me_4Si): δ -4.95, -4.40, 18.24, 25.73, 63.55, 80.51, 90.69, 110.36, 117.17, 117.38, 122.50, 122.94, 124.77, 125.01, 127.12, 128.25, 130.64, 132.95, 133.12, 138.66, 138.98, 145.33. IR (film): 3101, 3069, 2953, 2930, 2887, 2856, 2224, 1598, 1467, 1428, 1391, 1344, 1317, 1255, 1208, 1182, 1163, 1104, 1068, 1007, 938, 836, 778, 756, 730, 673, 629 cm^{-1}. HRMS (ESI) calcd for $C_{24}H_{29}N_2OSSi$ $[M+NH_4]^+$: 421.1764, found 421.1766.

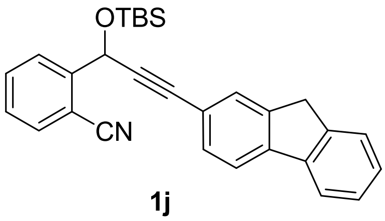

1j

2-(1-((*tert*-Butyldimethylsilyl)oxy)-3-(9*H*-fluoren-2-yl)prop-2-yn-1-yl)benzonitrile (1j). (4.0 mmol scale. First step: To a solution of 2-ethynyl-9*H*-fluorene (5.2 mmol, 989.0 mg), THF (15.0 mL) was added dropwise EtMgBr (3.0 M in diethyl ether, 4.8 mmol, 1.6 mL) at room temperature, and the mixture was stirred at 50 °C for 1.0 h, then 2-cyanobenzaldehyde (4 mmol, 524.5 mg) was added at room temperature and stirred for 2 h. Second step: DCM (15.0 mL), imidazole (8 mmol, 544.6 mg), TBSCl (6 mmol, 904.0 mg), stirred at room temperature overnight. Column chromatography on silica gel (eluent: petroleum ether: ethyl acetate = 100:1) afforded the title product in 73% overall yield (1.28 g) as a yellow oil. ^{1}H NMR(400 MHz, $CDCl_3$, Me_4Si): δ 0.29 (s, 3H), 0.35 (s, 3H), 1.01 (s, 9H), 3.87 (s, 2H), 6.06 (s, 1H), 7.30 (td, J = 7.2, 0.8 Hz, 1H), 7.38-7.44 (m, 2H), 7.49 (d, J = 7.6 Hz, 1H), 7.55 (d, J = 7.2 Hz, 1H), 7.64-7.73 (m, 4H), 7.77 (d, J = 7.6 Hz, 1H), 7.93 (d, J = 8.4 Hz, 1H). ^{13}C NMR(100 MHz, $CDCl_3$, Me_4Si): δ -4.93, -4.35, 18.26, 25.76, 36.61, 63.53, 87.43, 88.05, 110.43, 117.22, 119.63, 120.15, 120.26, 125.01, 126.83, 127.16, 127.19, 128.13, 128.15, 130.44, 132.94, 133.04, 140.87, 142.12, 143.03, 143.47, 145.43. IR (film): 3068, 3019, 2930, 2889, 2856, 2224, 1600, 1456, 1403, 1360, 1331, 1293, 1254,

1203, 1180, 1148, 1105, 1065, 1000, 951, 835, 762, 732, 690, 671, 647, 627 cm^{-1}. HRMS (ESI) calcd for $C_{29}H_{33}N_2OSi$ [M+NH$_4$]$^+$: 453.2357, found 453.2357.

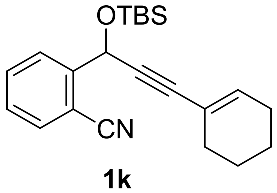

1k

2-(1-((*tert*-Butyldimethylsilyl)oxy)-3-(cyclohex-1-en-1-yl)prop-2-yn-1-yl)benzonitrile (1k). (5.0 mmol scale. First step: To a solution of 1-ethynylcyclohex-1-ene (6.5 mmol, 0.76 mL) in THF (15.0 mL) was added dropwise EtMgBr (3.0 M in diethyl ether, 5.5 mmol, 1.8 mL) at room temperature, and the mixture was stirred at 50 ^{o}C for 1.0 h, then 2-cyanobenzaldehyde (5 mmol, 655.7 mg) was added at room temperature and stirred for 2 h. Second step: DCM (15.0 mL), imidazole (10 mmol, 680.8 mg), TBSCl (7.5 mmol, 1.13 g), stirred at room temperature overnight. Column chromatography on silica gel (eluent: petroleum ether: ethyl acetate = 100:1) afforded the title product in 86% overall yield (1.51 g) as a yellow oil. ^{1}H NMR(400 MHz, $CDCl_3$, Me_4Si): δ 0.19 (s, 3H), 0.23 (s, 3H), 0.93 (s, 9H), 1.54-1.63 (m, 4H), 2.05-2.12 (m, 4H), 5.86 (s, 1H), 6.09-6.11 (m, 1H), 7.37 (td, *J* = 7.6, 1.2 Hz, 1H), 7.58-7.64 (m, 2H), 7.80 (dd, *J* = 8.0, 0.4 Hz, 1H). ^{13}C NMR(100 MHz, $CDCl_3$, Me_4Si): δ -4.99, -4.39, 18.21, 21.36, 22.11, 25.52, 25.74, 28.74, 63.41, 85.46, 88.61, 110.40, 117.20, 119.97, 127.15, 127.99, 132.86, 132.94, 135.48, 145.72. IR (film): 2931, 2887, 2857, 2224, 1468, 1447, 1359, 1340, 1254, 1199, 1179, 1105, 1066, 1002, 934, 838, 776, 761, 721, 673 cm^{-1}. HRMS (ESI) calcd for $C_{22}H_{33}N_2OSi$ [M+NH$_4$]$^+$: 369.2357, found 369.2357.

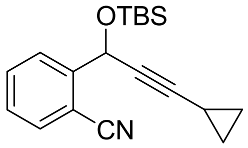

1l

2-(1-((*tert*-Butyldimethylsilyl)oxy)-3-cyclopropylprop-2-yn-1-yl)benzonitrile (1l). (5.0 mmol scale. First step: To a solution of ethynylcyclopropane (6.5 mmol, 0.60 mL) in THF (15.0 mL) was added dropwise EtMgBr (3.0 M in diethyl ether, 6 mmol, 2.0 mL) at room

temperature, and the mixture was stirred at 50 °C for 1.0 h, then 2-cyanobenzaldehyde (5 mmol, 655.7 mg) was added at room temperature and stirred for 2 h. Second step: DCM (15.0 mL), imidazole (10 mmol, 680.8 mg), TBSCl (7.5 mmol, 1.13 g), stirred at room temperature overnight. Column chromatography on silica gel (eluent: petroleum ether: ethyl acetate = 100:1) afforded the title product in 75% overall yield (1.17 g) as a yellow oil. ^{1}H NMR(400 MHz, $CDCl_3$, Me_4Si): δ 0.16 (s, 3H), 0.20 (s, 3H), 0.67-0.71 (m, 2H), 0.72-0.78 (m, 2H), 0.91 (s, 9H), 1.21-1.28 (m, 1H), 5.70 (d, J = 1.6 Hz, 1H), 7.35 (td, J = 7.8, 0.8 Hz, 1H), 7.57-7.62 (m, 2H), 7.76 (d, J = 7.6 Hz, 1H). ^{13}C NMR(100 MHz, $CDCl_3$, Me_4Si): δ -5.06, -4.49, -0.53, 8.00, 8.02, 18.15, 25.69, 63.08, 74.46, 90.65, 110.26, 117.15, 126.97, 127.89, 132.77, 132.88, 145.89. IR (film): 3825, 3802, 3680, 3561, 3436, 3095, 3073, 3013, 2954, 2931, 2888, 2857, 2741, 2710, 2650, 2480, 2359, 2227, 2078, 1946, 1837, 1600, 1469, 1359, 1254, 1158, 1106, 1068, 1027, 939, 897, 837, 775, 709, 671, 624 cm^{-1}. HRMS (ESI) calcd for $C_{19}H_{29}N_2OSi$ $[M+NH_4]^+$: 329.2044, found 329.2044.

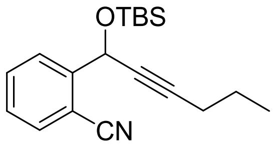

1m

2-(1-((*tert*-Butyldimethylsilyl)oxy)hex-2-yn-1-yl)benzonitrile (1m). (5.0 mmol scale. First step: To a solution of pent-1-yne (6 mmol, 0.60 mL) in THF (20.0 mL) was added dropwise EtMgBr (3.0 M in diethyl ether, 5.5 mmol, 1.8 mL) at 0 °C, and the mixture was stirred at room temperature for 4.0 h, then 2-cyanobenzaldehyde (5 mmol, 655.7 mg) was added at 0 °C and stirred at room temperature for 3.5 h. Second step: DCM (20.0 mL), imidazole (10 mmol, 680.8 mg), TBSCl (7.5 mmol, 1.13 g), stirred at room temperature overnight. Column chromatography on silica gel (eluent: petroleum ether: ethyl acetate = 30:1) afforded the title product in 85% overall yield (1.33 g) as a colorless oil. ^{1}H NMR(400 MHz, $CDCl_3$, Me_4Si): δ 0.17 (s, 3H), 0.21 (s, 3H), 0.92 (s, 9H), 0.96 (t, J = 7.2 Hz, 3H), 1.48-1.57 (m, 2H), 2.18 (td, J = 7.0, 2.0 Hz, 2H), 5.75 (t, J = 2.0 Hz, 1H), 7.36 (td, J = 7.8, 1.2 Hz, 1H), 7.58-7.63 (m, 2H), 7.80 (d, J = 7.6 Hz, 1H). ^{13}C NMR(100 MHz, $CDCl_3$, Me_4Si): δ -5.07, -4.52, 13.43, 18.19, 20.71, 21.77, 25.70, 63.06, 79.51, 87.57,

110.23, 117.19, 126.95, 127.90, 132.76, 132.92, 146.09. IR (film): 3071, 2941, 2862, 2283, 2229, 1940, 1600, 1464, 1332, 1256, 1209, 1139, 1060, 946, 842, 769, 717, 674 cm^{-1}. HRMS (ESI) calcd for $C_{19}H_{31}N_2OSi$ $[M+NH_4]^+$: 331.2200, found 331.2201.

Synthesis of 1-naphthylamines 2.

Typical procedure for the synthesis of 1-naphthylamine 2c.

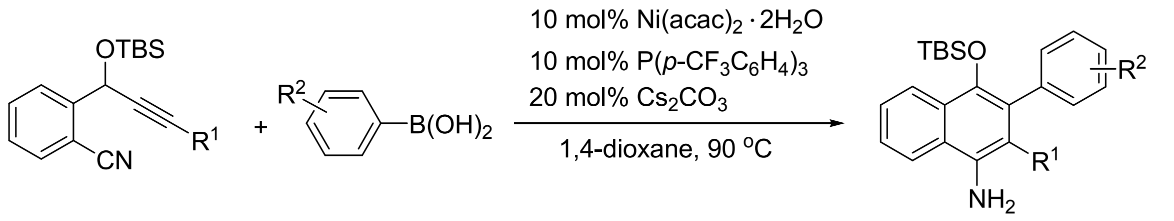

1a 2.0 equiv **2**

For **2c**, R^1 = Ph, R^2 = *p*-^{t}Bu

In a nitrogen-filled glovebox, $Ni(acac)_2{\cdot}2H_2O$ (0.03 mmol, 8.8 mg), P(*p*-$CF_3C_6H_4)_3$ (0.03 mmol, 14.0 mg), Cs_2CO_3 (0.06 mmol, 19.5 mg) and (4-(*tert*-butyl)phenyl)boronic acid (0.6 mmol, 106.8 mg) were added to an oven-dried screw-cap vial (volume: 4.0 mL), then 1,4-dioxane (2.0 mL) and *o*-(cyano)phenyl propargyl ether **1a** (104.3 mg, 0.3 mmol) were added. The vial cap was then securely fitted and sealed with electrical tape before the vial was removed from the glovebox. Then the vial was taken outside the glovebox and stirred at 90 °C in an oil-bath until the reaction was complete as monitored by TLC (4 h). After the mixture was cooled down to room temperature, the mixture was filtered through a short silica gel column and washed with ethyl acetate. The solvent was evaporated under the reduced pressure and the residue was purified by column chromatography on silica gel (eluent: petroleum ether: ethyl acetate = 15:1) to afford **2c** in 77% yield (111.7 mg) as a light yellow solid. M.p. = 175-176 °C.

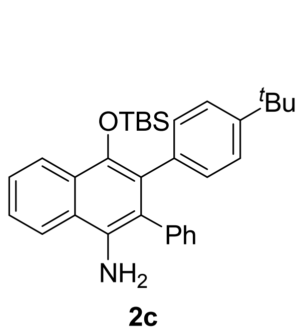

2c

3-(4-(*tert*-Butyl)phenyl)-4-((*tert*-butyldimethylsilyl)oxy)-2-phenylnaphthalen-1-amine (2c). ^{1}H NMR(400 MHz, C_6D_6, Me_4Si): δ -0.26 (s, 6H), 1.02 (s, 9H), 1.16 (s, 9H), 3.54 (s, 2H), 6.90-6.93 (m, 1H), 7.01 (t, *J* = 7.2 Hz, 2H), 7.09-7.15 (m, 4H), 7.21 (d, *J* = 8.4 Hz, 2H), 7.29 (td, *J* = 8.4, 1.2 Hz, 1H), 7.43 (t, *J* = 6.8 Hz, 1H), 7.57 (d, *J* = 8.4 Hz, 1H), 8.49 (d, *J* = 7.6 Hz, 1H). ^{13}C NMR(100 MHz, C_6D_6, Me_4Si): δ -3.77, 18.92, 26.51, 31.43, 34.39, 121.97, 123.21, 124.35, 124.51, 124.78, 125.32, 125.47, 126.82, 128.53, 128.92, 129.05, 131.70, 132.60, 134.70, 135.93, 139.48, 141.31, 148.97. IR (neat): 3477, 3462, 3371, 3078, 3052, 3029, 2957, 2928, 2884, 2857, 1614, 1579, 1515, 1492, 1467, 1443, 1392, 1370, 1254, 1178, 1145, 1108, 1071, 1031, 899, 834, 813, 779, 757, 732, 703, 673 cm^{-1}. HRMS (ESI) calcd for $C_{32}H_{40}NOSi$ $[M+H]^+$: 482.2874, found 482.2868.

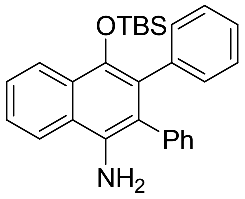

2a

4-((*tert*-Butyldimethylsilyl)oxy)-2,3-diphenylnaphthalen-1-amine (2a). (0.3 mmol scale, $Ni(acac)_2 \cdot 2H_2O$ (0.015 mmol, 4.4 mg), $P(p\text{-}CF_3C_6H_4)_3$ (0.015 mmol, 7.0 mg), Cs_2CO_3 (0.03 mmol, 9.8 mg), phenylboronic acid (0.6 mmol, 73.2 mg), 1,4-dioxane (2.0 mL) and *o*-(cyano)phenyl propargyl ether **1a** (104.3 mg, 0.3 mmol) were stirred at 90 °C for 3 h. Purification of the crude product by column chromatography on silica gel (eluent: petroleum ether: ethyl acetate = 13:1) afforded the title product in 73% yield (92.7 mg) as a light yellow solid. M.p. = 183-184 °C. ^{1}H NMR(400 MHz, C_6D_6, Me_4Si): δ -0.27 (s, 6H), 1.02 (s, 9H), 3.54 (s, 2H), 6.92-6.95 (m, 2H), 7.01 (td, *J* = 7.2, 3.6 Hz, 4H), 7.08-7.10 (m, 2H), 7.22-7.24 (m, 2H), 7.30 (t, *J* = 7.0 Hz, 1H), 7.43 (t, *J* = 7.6 Hz, 1H), 7.57 (d, *J* = 8.4 Hz, 1H), 8.49 (d, *J* = 8.4 Hz, 1H). ^{13}C NMR(100 MHz, C_6D_6, Me_4Si): δ -3.71, 18.88, 26.46, 121.97, 123.10, 124.52, 124.87, 125.37, 125.58, 126.31, 126.83, 127.43, 128.57, 128.90, 129.05, 131.68, 132.93, 134.74, 138.93, 139.39, 141.14. IR (neat): 3451, 3371, 3074, 3052, 3033, 2951, 2928, 2884, 2855, 1608, 1579, 1491, 1469, 1444, 1388, 1370, 1257, 1177, 1144, 1069, 1029, 920, 894, 873, 843, 827, 813, 786, 758, 734, 697, 678, 659 cm^{-1}. HRMS (ESI) calcd for $C_{28}H_{32}NOSi$ $[M+H]^+$: 426.2248, found 426.2246.

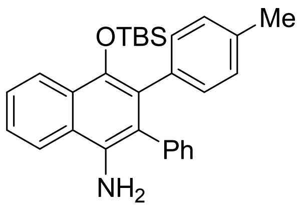

2b

4-((*tert*-Butyldimethylsilyl)oxy)-2-phenyl-3-(*p*-tolyl)naphthalen-1-amine (2b). (0.3 mmol scale, Ni(acac)$_2$·2H$_2$O (0.015 mmol, 4.4 mg), P(*p*-CF$_3$C$_6$H$_4$)$_3$ (0.015 mmol, 7.0 mg), Cs$_2$CO$_3$ (0.03 mmol, 9.8 mg), *p*-tolylboronic acid (0.6 mmol, 81.6 mg), 1,4-dioxane (2.0 mL) and *o*-(cyano)phenyl propargyl ether **1a** (104.3 mg, 0.3 mmol) were stirred at 90 °C for 8 h. Purification of the crude product by column chromatography on silica gel (eluent: petroleum ether: ethyl acetate = 20:1) afforded the title product in 73% yield (95.9 mg) as a light yellow solid. M.p. = 150-152 °C. ^{1}H NMR(400 MHz, C$_6$D$_6$, Me$_4$Si): δ -0.24 (s, 6H), 1.03 (s, 9H), 2.00 (s, 3H), 3.53 (s, 2H), 6.85 (d, *J* = 8.0 Hz, 2H), 6.94 (t, *J* = 7.2 Hz, 1H), 7.03 (t, *J* = 7.2 Hz, 2H), 7.11 (d, *J* = 7.2 Hz, 2H), 7.16 (d, *J* = 8.4 Hz, 2H), 7.30 (t, *J* = 7.2 Hz, 1H), 7.44 (t, *J* = 7.2 Hz, 1H), 7.58 (d, *J* = 8.4 Hz, 1H), 8.50 (d, *J* = 8.8 Hz, 1H). ^{13}C NMR(100 MHz, C$_6$D$_6$, Me$_4$Si): δ -3.70, 18.90, 21.16, 26.49, 121.96, 123.28, 124.50, 124.80, 125.34, 125.48, 126.81, 128.20, 128.57, 128.94, 129.03, 131.71, 132.84, 134.72, 135.58, 135.93, 139.58, 141.22. IR (neat): 3454, 3374, 3075, 3051, 3024, 2925, 2855, 1954, 1900, 1608, 1468, 1442, 1371, 1256, 1178, 1071, 1028, 897, 829, 784, 760, 738, 722, 700, 675, 659 cm^{-1}. HRMS (ESI) calcd for C$_{29}$H$_{34}$NOSi [M+H]$^+$: 440.2404, found 440.2399.

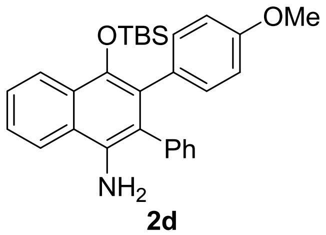

2d

4-((*tert*-Butyldimethylsilyl)oxy)-3-(4-methoxyphenyl)-2-phenylnaphthalen-1-amine (2d). (0.3 mmol scale, Ni(acac)$_2$·2H$_2$O (0.03 mmol, 8.8 mg), P(*p*-CF$_3$C$_6$H$_4$)$_3$ (0.03 mmol, 14.0 mg), Cs$_2$CO$_3$ (0.06 mmol, 19.5 mg), (4-methoxyphenyl)boronic acid (0.6 mmol, 91.2 mg), 1,4-dioxane (2.0 mL) and *o*-(cyano)phenyl propargyl ether **1a** (104.3 mg, 0.3 mmol) were stirred at 90 °C for 6 h. Purification of the crude product by column chromatography

on silica gel which was treated with petroleum ether/Et_3N = 1:1 and then petroleum ether before loading the sample (eluent: petroleum ether: ethyl acetate = 15:1) afforded the title product in 68% yield (93.2 mg) as a yellow solid. M.p. = 162-163 °C. ^{1}H NMR(400 MHz, C_6D_6, Me_4Si): δ -0.23 (s, 6H), 1.04 (s, 9H), 3.20 (s, 3H), 3.57 (s, 2H), 6.64 (d, *J* = 8.8 Hz, 2H), 6.95 (t, *J* = 7.2 Hz, 1H), 7.04 (t, *J* = 7.2 Hz, 2H), 7.11-7.13 (m, 2H), 7.14-7.16 (m, 2H), 7.30 (t, *J* = 7.4 Hz, 1H), 7.44 (t, *J* = 7.2 Hz, 1H), 7.59 (d, *J* = 8.8 Hz, 1H), 8.50 (d, *J* = 8.4 Hz, 1H). ^{13}C NMR(100 MHz, C_6D_6, Me_4Si): δ -3.70, 18.89, 26.49, 54.58, 113.01, 121.96, 123.38, 124.47, 124.73, 125.35, 125.44, 126.81, 128.63, 128.74, 128.95, 131.06, 131.71, 133.93, 134.73, 139.62, 141.24, 158.51. IR (neat): 3458, 3378, 3073, 3035, 2951, 2928, 2886, 2856, 1605, 1568, 1512, 1465, 1445, 1417, 1372, 1285, 1243, 1173, 1143, 1073, 1028, 899, 831, 773, 759, 701, 673, 643 cm^{-1}. HRMS (ESI) calcd for $C_{29}H_{34}NO_2Si$ $[M+H]^+$: 456.2353, found 456.2341.

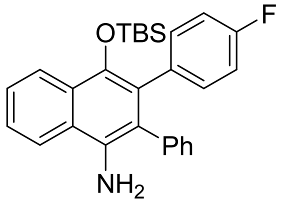

2e

4-((*tert*-Butyldimethylsilyl)oxy)-3-(4-fluorophenyl)-2-phenylnaphthalen-1-amine (2e). (0.3 mmol scale, $Ni(acac)_2 \cdot 2H_2O$ (0.03 mmol, 8.8 mg), P(*p*-$CF_3C_6H_4$)$_3$ (0.03 mmol, 14.0 mg), Cs_2CO_3 (0.06 mmol, 19.5 mg), (4-fluorophenyl)boronic acid (0.6 mmol, 84.0 mg), 1,4-dioxane (2.0 mL) and *o*-(cyano)phenyl propargyl ether **1a** (104.3 mg, 0.3 mmol) were stirred at 90 °C for 5 h. Purification of the crude product by column chromatography on silica gel (eluent: petroleum ether: ethyl acetate = 13:1) afforded the title product in 68% yield (90.1 mg) as a light yellow solid. M.p. = 148-149 °C. ^{1}H NMR(400 MHz, C_6D_6, Me_4Si): δ -0.30 (s, 6H), 0.99 (s, 9H), 3.53 (bs, 2H), 6.67 (t, *J* = 8.8 Hz, 2H), 6.92-6.98 (m, 1H), 7.01-7.04 (m, 6H), 7.28 (t, *J* = 8.0 Hz, 1H), 7.42 (t, *J* = 8.0 Hz, 1H), 7.55 (d, *J* = 8.8 Hz, 1H), 8.44 (d, *J* = 8.4 Hz, 1H). ^{13}C NMR(100 MHz, C_6D_6, Me_4Si): δ -3.72, 18.81, 26.35, 114.34 (d, *J* = 21 Hz), 121.95, 122.87, 124.45, 124.85, 125.46, 125.70, 126.97, 127.91, 128.66, 128.82, 131.61, 134.41 (d, *J* = 7.6 Hz), 134.76, 134.82 (d, *J* = 3.5 Hz), 139.16, 141.13, 161.80 (d, *J* = 245.4 Hz). IR (neat): 3458, 3377, 3077, 3055, 2949, 2928, 2888,

2855, 1604, 1510, 1469, 1443, 1371, 1258, 1220, 1182, 1154, 1073, 1029, 901, 832, 809, 780, 760, 740, 702, 676, 658 cm^{-1}. HRMS (ESI) calcd for $C_{28}H_{31}FNOSi$ [M+H]$^+$: 444.2153, found 444.2148.

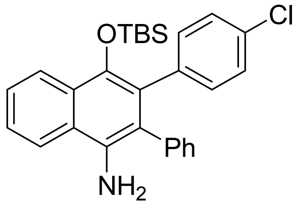

2f

4-((*tert*-Butyldimethylsilyl)oxy)-3-(4-chlorophenyl)-2-phenylnaphthalen-1-amine (2f). (0.3 mmol scale, Ni(acac)$_2$·2H$_2$O (0.03 mmol, 8.8 mg), P(*p*-CF$_3$C$_6$H$_4$)$_3$ (0.03 mmol, 14.0 mg), Cs_2CO_3 (0.06 mmol, 19.5 mg), (4-chlorophenyl)boronic acid (0.6 mmol, 93.8 mg), 1,4-dioxane (2.0 mL) and *o*-(cyano)phenyl propargyl ether **1a** (104.3 mg, 0.3 mmol) were stirred at 90 ^oC for 3 h. Purification of the crude product by column chromatography on silica gel (eluent: petroleum ether: ethyl acetate = 15:1) afforded the title product in 69% yield (95.4 mg) as a yellow solid. M.p. = 154-155 ^oC. ^1H NMR(400 MHz, C_6D_6, Me_4Si): δ -0.32 (s, 6H), 0.97 (s, 9H), 3.52 (s, 2H), 6.91-6.95 (m, 1H), 6.97-7.00 (m, 8H), 7.28 (t, J = 7.4 Hz, 1H), 7.41 (t, J = 7.2 Hz, 1H), 7.53 (d, J = 8.4 Hz, 1H), 8.43 (d, J = 8.0 Hz, 1H). ^{13}C NMR(100 MHz, C_6D_6, Me_4Si): δ -3.73, 18.79, 26.31, 121.94, 122.55, 124.45, 124.89, 125.50, 125.78, 127.08, 127.64, 127.69, 128.70, 128.79, 131.58, 132.33, 134.25, 134.82, 137.40, 138.96, 141.06. IR (neat): 3456, 3374, 3078, 3058, 2949, 2928, 2887, 2855, 1607, 1579, 1492, 1470, 1442, 1370, 1300, 1258, 1183, 1145, 1090, 1073, 1029, 1015, 901, 830, 782, 761, 739, 703, 675 cm^{-1}. HRMS (ESI) calcd for $C_{28}H_{31}ClNOSi$ [M+H]$^+$: 460.1858, found 460.1857.

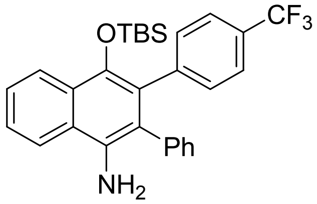

2g

4-((*tert*-Butyldimethylsilyl)oxy)-2-phenyl-3-(4-(trifluoromethyl)phenyl)naphthalen-1-amine (2g). (0.3 mmol scale, $Ni(acac)_2{\cdot}2H_2O$ (0.03 mmol, 8.8 mg), $P(p\text{-}CF_3C_6H_4)_3$ (0.03 mmol, 14.0 mg), Cs_2CO_3 (0.06 mmol, 19.5 mg), (4-(trifluoromethyl)phenyl)boronic acid (0.6 mmol, 114.0 mg), 1,4-dioxane (2.0 mL) and *o*-(cyano)phenyl propargyl ether **1a** (104.3 mg, 0.3 mmol) were stirred at 90 °C for 4 h. Purification of the crude product by column chromatography on silica gel (eluent: petroleum ether: ethyl acetate = 15:1) afforded the title product in 76% yield (112.2 mg) as a yellow solid. M.p. = 160-161 °C. ^{1}H NMR(400 MHz, C_6D_6, Me_4Si): δ -0.36 (s, 6H), 0.94 (s, 9H), 3.53 (s, 2H), 6.88-6.93 (m, 1H), 6.96-6.97 (m, 4H), 7.14 (d, J = 8.0 Hz, 2H), 7.23 (d, J = 8.0 Hz, 2H), 7.28 (t, J = 7.6 Hz, 1H), 7.41 (t, J = 7.6 Hz, 1H), 7.53 (d, J = 8.4 Hz, 1H), 8.42 (d, J = 8.0 Hz, 1H). ^{13}C NMR(100 MHz, C_6D_6, Me_4Si): δ -3.81, 18.75, 26.20, 121.96, 122.21, 124.27 (q, J = 3.5 Hz), 124.50, 125.05, 125.08 (q, J = 272 Hz), 125.59, 126.00, 127.21, 127.58, 128.45 (q, J = 31.9 Hz), 128.71, 128.74, 131.53, 133.22, 134.90, 138.67, 141.12, 142.95 (q, J = 1.4 Hz). IR (neat): 3453, 3373, 3075, 3055, 3021, 2949, 2927, 2884, 2855, 1608, 1492, 1444, 1408, 1390, 1371, 1325, 1258, 1170, 1122, 1065, 1017, 900, 838, 784, 761, 703, 677, 661 cm^{-1}. HRMS (ESI) calcd for $C_{29}H_{31}F_3NOSi$ $[M+H]^+$: 494.2122, found 494.2117.

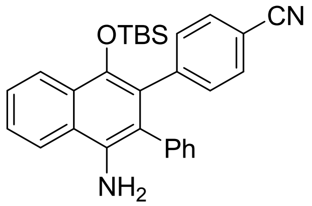

2h

4-(4-Amino-1-((*tert*-butyldimethylsilyl)oxy)-3-phenylnaphthalen-2-yl)benzonitrile (2h). (0.3 mmol scale, $Ni(acac)_2{\cdot}2H_2O$ (0.03 mmol, 8.8 mg), $P(p\text{-}CF_3C_6H_4)_3$ (0.03 mmol, 14.0 mg), Cs_2CO_3 (0.06 mmol, 19.5 mg), (4-cyanophenyl)boronic acid (0.6 mmol, 88.2 mg), tetrahydrofuran (2.0 mL) and *o*-(cyano)phenyl propargyl ether **1a** (104.3 mg, 0.3 mmol) were stirred at 90 °C for 3 h. Purification of the crude product by column chromatography on silica gel (eluent: petroleum ether: ethyl acetate = 10:1) afforded the title product in 74% yield (100.2 mg) as a yellow solid. M.p. = 91-92 °C. ^{1}H NMR(400 MHz, C_6D_6, Me_4Si): δ -0.41 (s, 6H), 0.92 (s, 9H), 3.59 (bs, 2H), 6.90-7.01 (m, 9H), 7.29 (t, J = 6.8 Hz, 1H), 7.41 (t,

J = 7.2 Hz, 1H), 7.56 (d, J = 8.4 Hz, 1H), 8.38 (d, J = 8.4 Hz, 1H). ^{13}C NMR(100 MHz, C_6D_6, Me_4Si): δ -3.78, 18.71, 26.20, 110.23, 119.06, 121.73, 122.03, 124.43, 125.09, 125.68, 126.16, 127.21, 127.27, 128.64, 128.76, 130.91, 131.49, 133.39, 135.04, 138.51, 140.89, 143.71. IR (neat): 3456, 3378, 3077, 3050, 2953, 2930, 2890, 2857, 2361, 2226, 1952, 1924, 1735, 1705, 1606, 1578, 1497, 1467, 1441, 1374, 1305, 1254, 1176, 1146, 1074, 1012, 904, 837, 763, 703, 677, 641 cm^{-1}. HRMS (ESI) calcd for $C_{29}H_{31}N_2OSi$ $[M+H]^+$: 451.2200, found 451.2206.

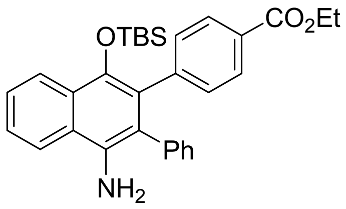

2i

Ethyl 4-(4-amino-1-((*tert*-butyldimethylsilyl)oxy)-3-phenylnaphthalen-2-yl)benzoate (2i). (0.3 mmol scale, $Ni(acac)_2 \cdot 2H_2O$ (0.03 mmol, 8.8 mg), P(p-$CF_3C_6H_4)_3$ (0.03 mmol, 14.0 mg), Cs_2CO_3 (0.06 mmol, 19.5 mg), (4-(ethoxycarbonyl)phenyl)boronic acid (0.6 mmol, 116.4 mg), 1,4-dioxane (2.0 mL) and *o*-(cyano)phenyl propargyl ether **1a** (104.3 mg, 0.3 mmol) were stirred at 90 °C for 5 h. Purification of the crude product by column chromatography on silica gel (eluent: petroleum ether: ethyl acetate = 10:1) afforded the title product in 64% yield (96.0 mg) as a light yellow solid. M.p. = 150-151 °C. ^{1}H NMR(400 MHz, C_6D_6, Me_4Si): δ -0.33 (s, 6H), 0.92 (t, J = 6.8 Hz, 3H), 0.96 (s, 9H), 3.60 (bs, 2H), 4.01 (q, J = 7.2 Hz, 2H), 6.88-6.92 (m, 1H), 6.97-7.04 (m, 4H), 7.26 (d, J = 7.6 Hz, 2H), 7.30 (td, J = 7.8, 1.2 Hz, 1H), 7.42 (td, J = 8.4, 0.8 Hz, 1H), 7.59 (d, J = 8.0 Hz, 1H), 8.04 (dd, J = 6.8, 1.6 Hz, 2H), 8.44 (d, J = 8.0 Hz, 1H). ^{13}C NMR(100 MHz, C_6D_6, Me_4Si): δ -3.68, 14.17, 18.78, 26.30, 60.70, 122.01, 122.42, 124.51, 125.05, 125.52, 125.90, 127.13, 128.11, 128.71, 128.77, 128.79, 131.56, 133.01, 134.92, 138.93, 141.08, 143.97, 166.24. IR (neat): 3459, 3378, 3079, 3058, 2947, 2927, 2883, 2854, 1713, 1606, 1492, 1469, 1445, 1388, 1364, 1274, 1178, 1145, 1128, 1108, 1071, 1022, 900, 854, 841, 827, 813, 784, 761, 737, 706, 675, 658 cm^{-1}. HRMS (ESI) calcd for $C_{31}H_{36}NO_3Si$ $[M+H]^+$: 498.2459, found 498.2461.

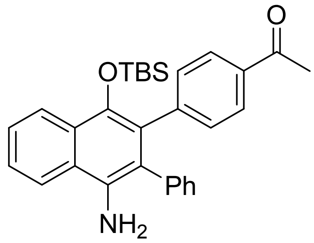

2j

1-(4-(4-Amino-1-((*tert*-butyldimethylsilyl)oxy)-3-phenylnaphthalen-2-yl)phenyl)ethanone (2j). (0.3 mmol scale, Ni(acac)$_2$·2H_2O (0.03 mmol, 8.8 mg), P(*p*-$CF_3C_6H_4$)$_3$ (0.03 mmol, 14.0 mg), Cs_2CO_3 (0.06 mmol, 19.5 mg), (4-acetylphenyl)boronic acid (0.6 mmol, 98.4 mg), tetrahydrofuran (2.0 mL) and *o*-(cyano)phenyl propargyl ether **1a** (104.3 mg, 0.3 mmol) were stirred at 90 ^{o}C for 6 h. Purification of the crude product by column chromatography on silica gel (eluent: petroleum ether: ethyl acetate = 10:1) afforded the title product in 70% yield (97.7 mg) as a yellow solid. M.p. = 161-162 ^{o}C. ^{1}H NMR(400 MHz, C_6D_6, Me_4Si): δ -0.32 (s, 6H), 0.98 (s, 9H), 2.00 (s, 3H), 3.64 (bs, 2H), 6.93 (t, *J* = 6.8 Hz, 1H), 7.00-7.06 (m, 4H), 7.24 (d, *J* = 8.4 Hz, 2H), 7.31 (t, *J* = 7.2 Hz, 1H), 7.43 (t, *J* = 7.2 Hz, 1H), 7.62 (d, *J* = 8.8 Hz, 1H), 7.68 (d, *J* = 8.4 Hz, 2H), 8.45 (d, *J* = 8.4 Hz, 1H). ^{13}C NMR(100 MHz, C_6D_6, Me_4Si): δ -3.69, 18.80, 26.07, 26.31, 122.06, 122.28, 124.50, 125.05, 125.56, 125.95, 127.15, 127.45, 128.05, 128.74, 128.79, 131.59, 133.06, 135.03, 135.32, 139.00, 141.07, 143.98, 196.31. IR (neat): 3468, 3375, 3081, 3058, 3033, 2953, 2926, 2888, 2855, 1665, 1626, 1601, 1579, 1558, 1493, 1465, 1427, 1374, 1253, 1185, 1147, 1075, 1009, 955, 906, 839, 765, 728, 699, 672 cm^{-1}. HRMS (ESI) calcd for $C_{30}H_{34}NO_2Si$ [M+H]$^+$: 468.2353, found 468.2354.

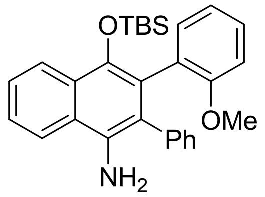

2k

4-((*tert*-Butyldimethylsilyl)oxy)-3-(2-methoxyphenyl)-2-phenylnaphthalen-1-amine (2k). (0.3 mmol scale, Ni(acac)$_2$·2H_2O (0.03 mmol, 8.8 mg), P(*p*-$CF_3C_6H_4$)$_3$ (0.03 mmol,

14.0 mg), Cs_2CO_3 (0.06 mmol, 19.5 mg), (2-methoxyphenyl)boronic acid (0.6 mmol, 91.2 mg), 1,4-dioxane (2.0 mL) and *o*-(cyano)phenyl propargyl ether **1a** (104.3 mg, 0.3 mmol) were stirred at 90 °C for 10 h. Purification of the crude product by column chromatography on silica gel (eluent: petroleum ether: ethyl acetate = 10:1) afforded the title product in 47% yield (64.7 mg) as a light yellow solid. M.p. = 159-160 °C. ^{1}H NMR(400 MHz, C_6D_6, Me_4Si): δ -0.26 (s, 3H), -0.06 (s, 3H), 1.03 (s, 9H), 3.09 (s, 3H), 3.54 (bs, 2H), 6.30 (d, *J* = 8.4 Hz, 1H), 6.79 (t, *J* = 7.6 Hz, 1H), 6.94-7.00 (m, 4H), 7.09-7.11 (m, 1H), 7.27-7.31 (m, 2H), 7.39-7.45 (m, 2H), 7.60 (d, *J* = 8.4 Hz, 1H), 8.48 (d, *J* = 8.4 Hz, 1H). ^{13}C NMR(100 MHz, C_6D_6, Me_4Si): δ -4.34, -3.49, 18.79, 26.46, 54.11, 110.19, 119.88, 121.98, 124.10, 124.38, 125.05, 125.18, 125.34, 126.09, 126.57, 127.52, 127.97, 128.52, 128.92, 131.40, 131.48, 134.10, 139.58, 141.55, 157.36. IR (neat): 3441, 3375, 3053, 3026, 2952, 2929, 2890, 2855, 1608, 1580, 1494, 1463, 1436, 1374, 1298, 1246, 1180, 1148, 1115, 1072, 1028, 905, 829, 751, 701, 676 cm^{-1}. HRMS (ESI) calcd for $C_{29}H_{34}NO_2Si$ $[M+H]^+$: 456.2353, found 456.2360.

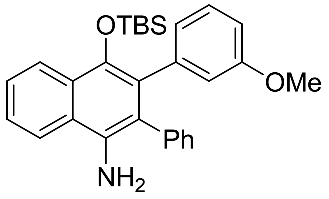

2l

4-((*tert*-Butyldimethylsilyl)oxy)-3-(3-methoxyphenyl)-2-phenylnaphthalen-1-amine (2l). (0.3 mmol scale, $Ni(acac)_2 \cdot 2H_2O$ (0.03 mmol, 8.8 mg), $P(p\text{-}CF_3C_6H_4)_3$ (0.03 mmol, 14.0 mg), Cs_2CO_3 (0.06 mmol, 19.5 mg), (3-methoxyphenyl)boronic acid (0.6 mmol, 91.2 mg), 1,4-dioxane (2.0 mL) and *o*-(cyano)phenyl propargyl ether **1a** (104.3 mg, 0.3 mmol) were stirred at 90 °C for 7.5 h. Purification of the crude product by column chromatography on silica gel (eluent: petroleum ether: ethyl acetate = 13:1) afforded the title product in 75% yield (102.7 mg) as a yellow solid. M.p. = 159-160 °C. ^{1}H NMR(400 MHz, C_6D_6, Me_4Si): δ -0.21 (s, 6H), 1.04 (s, 9H), 3.28 (s, 3H), 3.56 (s, 2H), 6.61-6.64 (m, 1H), 6.87-6.88 (m, 2H), 6.92-6.96 (m, 2H), 7.02 (t, *J* = 7.2 Hz, 2H), 7.11 (d, *J* = 7.2 Hz, 2H), 7.30 (t, *J* = 7.6 Hz, 1H), 7.43 (t, *J* = 7.2 Hz, 1H), 7.58 (d, *J* = 8.4 Hz, 1H), 8.50 (d, *J* = 8.0 Hz, 1H). ^{13}C NMR(100 MHz, C_6D_6, Me_4Si): δ -3.69, 18.83, 26.46, 54.73, 112.67,

118.30, 121.97, 123.01, 124.52, 124.85, 125.40, 125.53, 125.61, 126.85, 128.33, 128.60, 128.90, 128.97, 131.58, 134.74, 139.48, 140.08, 141.12, 159.30. IR (neat): 3456, 3375, 3074, 3057, 2950, 2926, 2888, 2854, 1601, 1490, 1464, 1428, 1372, 1313, 1284, 1246, 1213, 1161, 1069, 1042, 1007, 935, 898, 838, 810, 775, 759, 737, 699, 677 cm^{-1}. HRMS (ESI) calcd for $C_{29}H_{34}NO_2Si$ $[M+H]^+$: 456.2353, found 456.2351.

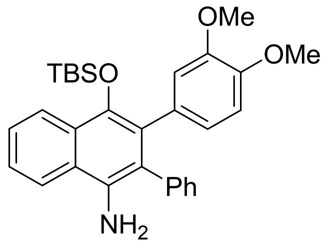

2m

4-((*tert*-Butyldimethylsilyl)oxy)-3-(3,4-dimethoxyphenyl)-2-phenylnaphthalen-1-amine (2m). (0.3 mmol scale, $Ni(acac)_2 \cdot 2H_2O$ (0.03 mmol, 8.8 mg), P(*p*-$CF_3C_6H_4)_3$ (0.03 mmol, 14.0 mg), Cs_2CO_3 (0.06 mmol, 19.5 mg), (3,4-dimethoxyphenyl)boronic acid (0.6 mmol, 109.2 mg), 1,4-dioxane (2.0 mL) and *o*-(cyano)phenyl propargyl ether **1a** (104.3 mg, 0.3 mmol) were stirred at 90 °C for 10.5 h. Purification of the crude product by column chromatography on silica gel (eluent: petroleum ether: ethyl acetate : dichloromethane = 15:1:1) afforded the title product in 65% yield (94.2 mg) as a yellow solid. M.p. = 188-189 °C. ^1H NMR(400 MHz, C_6D_6, Me_4Si): δ -0.20 (s, 6H), 1.06 (s, 9H), 3.31 (s, 3H), 3.38 (s, 3H), 3.59 (bs, 2H), 6.48 (d, J = 8.4 Hz, 1H), 6.72 (d, J = 1.6 Hz, 1H), 6.87 (dd, J = 8.2, 2.0 Hz, 1H), 6.93 (t, J = 7.6 Hz, 1H), 7.03 (t, J = 7.6 Hz, 2H), 7.13-7.16 (m, 2H), 7.31 (t, J = 8.0 Hz, 1H), 7.45 (t, J = 7.6 Hz, 1H), 7.62 (d, J = 8.4 Hz, 1H), 8.53 (d, J = 8.4 Hz, 1H). ^{13}C NMR(100 MHz, C_6D_6, Me_4Si): δ -3.70, 18.87, 26.48, 55.38, 55.59, 111.13, 117.27, 121.97, 123.30, 124.49, 124.73, 125.41, 125.49, 125.50, 126.79, 128.63 (br), 128.96, 129.03, 131.19, 131.64 (br), 134.76, 139.81, 141.23, 148.62, 149.06. IR (neat): 3451, 3371, 3075, 2997, 2951, 2928, 2856, 1737, 1607, 1585, 1514, 1493, 1465, 1412, 1369, 1313, 1250, 1188, 1159, 1135, 1075, 1025, 944, 893, 864, 831, 780, 758, 703, 673 cm^{-1}. HRMS (ESI) calcd for $C_{30}H_{36}NO_3Si$ $[M+H]^+$: 486.2459, found 486.2469.

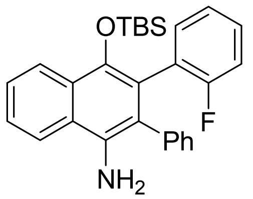

2n

4-((*tert*-Butyldimethylsilyl)oxy)-3-(2-fluorophenyl)-2-phenylnaphthalen-1-amine (2n). (0.3 mmol scale, Ni(acac)$_2$·2H$_2$O (0.03 mmol, 8.8 mg), P(*p*-CF$_3$C$_6$H$_4$)$_3$ (0.03 mmol, 14.0 mg), Cs$_2$CO$_3$ (0.06 mmol, 19.5 mg), (2-fluorophenyl)boronic acid (0.6 mmol, 84.0 mg), 1,4-dioxane (2.0 mL) and *o*-(cyano)phenyl propargyl ether **1a** (104.3 mg, 0.3 mmol) were stirred at 90 ^{o}C for 11 h. Purification of the crude product by column chromatography on silica gel which was treated with petroleum ether/Et$_3$N = 1:1 and then petroleum ether before loading the sample (eluent: petroleum ether: ethyl acetate = 15:1) afforded the title product in 41% yield (54.5 mg) as a light yellow solid. M.p. = 192-193 ^{o}C. ^{1}H NMR(400 MHz, C$_6$D$_6$, Me$_4$Si): δ -0.21 (s, 3H), -0.19 (s, 3H), 1.00 (s, 9H), 3.51 (s, 2H), 6.66-6.72 (m, 2H), 6.74-6.79 (m, 1H), 6.92-7.07 (m, 4H), 7.12-7.15 (m, 1H), 7.30 (t, *J* = 7.2 Hz, 1H), 7.36 (d, *J* = 8.0 Hz, 1H), 7.41 (t, *J* = 7.2 Hz, 1H), 7.57 (d, *J* = 8.4 Hz, 1H), 8.46 (d, *J* = 8.4 Hz, 1H). ^{13}C NMR(100 MHz, C$_6$D$_6$, Me$_4$Si): δ -3.99, -3.60, 18.75, 26.30, 115.43 (d, *J* = 22.5 Hz), 122.03, 123.18 (d, *J* = 3.1 Hz), 123.39, 124.50, 125.32, 125.36, 125.79, 127.02, 127.18 (d, *J* = 16.4 Hz), 128.20, 128.63, 128.67, 128.85 (d, *J* = 7.9 Hz), 131.36, 134.58 (d, *J* = 3.8 Hz), 134.65, 138.96, 141.83, 160.84 (d, *J* = 246.1 Hz). IR (neat): 3458, 3382, 3077, 3042, 2951, 2929, 2889, 2856, 1608, 1575, 1493, 1466, 1441, 1375, 1258, 1228, 1179, 1147, 1103, 1072, 1028, 902, 883, 828, 784, 755, 702, 677, 656 cm^{-1}. HRMS (ESI) calcd for C$_{28}$H$_{31}$FNOSi [M+H]$^+$: 444.2153, found 444.2157.

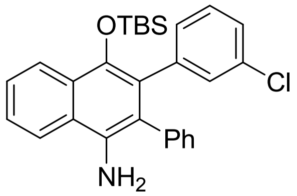

2o

4-((*tert*-Butyldimethylsilyl)oxy)-3-(3-chlorophenyl)-2-phenylnaphthalen-1-amine (2o). (0.3 mmol scale, Ni(acac)$_2$·2H$_2$O (0.015 mmol, 4.4 mg), P(*p*-CF$_3$C$_6$H$_4$)$_3$ (0.015 mmol, 7.0 mg), Cs$_2$CO$_3$ (0.03 mmol, 9.8 mg), (3-chlorophenyl)boronic acid (0.6 mmol, 93.8 mg), 1,4-

dioxane (2.0 mL) and *o*-(cyano)phenyl propargyl ether **1a** (104.3 mg, 0.3 mmol) were stirred at 90 °C for 3 h. Purification of the crude product by column chromatography on silica gel (eluent: petroleum ether: ethyl acetate = 15:1) afforded the title product in 68% yield (93.2 mg) as a light yellow solid. M.p. = 183-184 °C. ^{1}H NMR(400 MHz, C_6D_6, Me_4Si): δ -0.28 (bs, 6H), 1.02 (s, 9H), 3.51 (s, 2H), 6.68 (t, *J* = 8.0 Hz, 1H), 6.89-7.01 (m, 7H), 7.29 (t, *J* = 7.8, 1.2 Hz, 1H), 7.40-7.44 (m, 2H), 7.54 (d, *J* = 8.4 Hz, 1H), 8.44 (dd, *J* = 8.4, 0.4 Hz, 1H). ^{13}C NMR(100 MHz, C_6D_6, Me_4Si): δ -3.73, 18.73, 26.33, 121.95, 122.47, 124.51, 124.99, 125.52, 125.86, 126.38, 127.10, 127.53, 128.58, 128.770 (br), 128.774, 130.98, 131.56 (br), 133.10, 133.44, 134.85, 138.90, 140.84, 141.09. IR (neat): 3453, 3373, 3076, 3059, 2927, 2890, 2855, 1609, 1595, 1574, 1491, 1470, 1426, 1407, 1372, 1258, 1224, 1183, 1147, 1094, 1069, 1028, 1005, 914, 871, 844, 828, 810, 786, 775, 759, 722, 704, 678 cm^{-1}. HRMS (ESI) calcd for $C_{28}H_{31}ClNOSi$ $[M+H]^+$: 460.1858, found 460.1860.

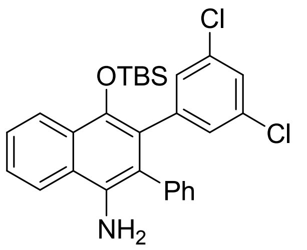

2p

4-((*tert*-Butyldimethylsilyl)oxy)-3-(3,5-dichlorophenyl)-2-phenylnaphthalen-1-amine (2p). (0.3 mmol scale, $Ni(acac)_2 \cdot 2H_2O$ (0.03 mmol, 8.8 mg), $P(p\text{-}CF_3C_6H_4)_3$ (0.03 mmol, 14.0 mg), Cs_2CO_3 (0.06 mmol, 19.5 mg), (3,5-dichlorophenyl)boronic acid (0.6 mmol, 114.5 mg), 1,4-dioxane (2.0 mL) and *o*-(cyano)phenyl propargyl ether **1a** (104.3 mg, 0.3 mmol) were stirred at 90 °C for 2 h. Purification of the crude product by column chromatography on silica gel (eluent: petroleum ether: ethyl acetate = 15:1) afforded the title product in 65% yield (96.8 mg) as a light yellow solid. M.p. = 142-143 °C. ^{1}H NMR(400 MHz, C_6D_6, Me_4Si): δ -0.30 (s, 6H), 1.02 (s, 9H), 3.50 (bs, 2H), 6.95-6.98 (m, 4H), 7.00-7.03 (m, 2H), 7.12 (d, *J* = 2.0 Hz, 2H), 7.28 (t, *J* = 7.4 Hz, 1H), 7.40 (t, *J* = 7.2 Hz, 1H), 7.50 (d, *J* = 8.4 Hz, 1H), 8.39 (d, *J* = 8.8 Hz, 1H). ^{13}C NMR(100 MHz, C_6D_6, Me_4Si): δ -3.79, 18.61, 26.22, 121.88, 121.97, 124.49, 125.10, 125.65, 126.12, 126.21, 126.37, 127.43, 128.65, 128.92, 131.38, 131.42, 134.00, 134.95, 138.40, 141.05, 142.06.

IR (neat): 3454, 3374, 3098, 3079, 3057, 3027, 2956, 2929, 2893, 2857, 1960, 1740, 1713, 1693, 1613, 1580, 1556, 1492, 1462, 1412, 1373, 1252, 1183, 1150, 1117, 1077, 1030, 1000, 931, 894, 870, 854, 830, 801, 775, 760, 706, 688, 671 cm^{-1}. HRMS (ESI) calcd for $C_{28}H_{30}Cl_2NOSi$ $[M+H]^+$: 494.1468, found 494.1468.

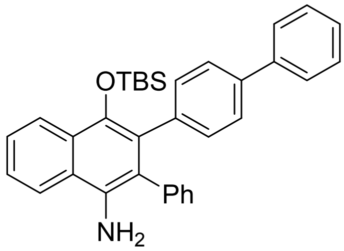

2q

3-([1,1'-Biphenyl]-4-yl)-4-((*tert*-butyldimethylsilyl)oxy)-2-phenylnaphthalen-1-amine (2q). (0.3 mmol scale, $Ni(acac)_2 \cdot 2H_2O$ (0.03 mmol, 8.8 mg), $P(p\text{-}CF_3C_6H_4)_3$ (0.03 mmol, 14.0 mg), Cs_2CO_3 (0.06 mmol, 19.5 mg), [1,1'-biphenyl]-4-ylboronic acid (0.6 mmol, 118.8 mg), 1,4-dioxane (2.0 mL) and *o*-(cyano)phenyl propargyl ether **1a** (104.3 mg, 0.3 mmol) were stirred at 90 °C for 6 h. Purification of the crude product by column chromatography on silica gel (eluent: petroleum ether: ethyl acetate = 15:1) afforded the title product in 73% yield (109.3 mg) as a yellow solid. M.p. = 180-181 °C. ^1H NMR(400 MHz, C_6D_6, Me_4Si): δ -0.24 (s, 6H), 1.02 (s, 9H), 3.58 (s, 2H), 6.95 (t, *J* = 7.2 Hz, 1H), 7.01-7.09 (m, 3H), 7.12-7.16 (m, 4H), 7.28-7.34 (m, 5H), 7.37-7.39 (m, 2H), 7.45 (t, *J* = 8.0 Hz, 1H), 7.59 (d, *J* = 8.4 Hz, 1H), 8.51 (d, *J* = 8.0 Hz, 1H). ^{13}C NMR(100 MHz, C_6D_6, Me_4Si): δ -3.69, 18.89, 26.44, 122.00, 122.96, 124.53, 124.89, 125.44, 125.66, 126.10, 126.96, 127.23, 127.27, 128.66, 128.93, 128.96, 131.74, 133.38, 134.83, 137.96, 139.06, 139.37, 141.22, 141.28. IR (neat): 3452, 3367, 3076, 3056, 3029, 2953, 2928, 2884, 2855, 1611, 1579, 1488, 1469, 1442, 1371, 1255, 1180, 1147, 1075, 1007, 904, 838, 778, 760, 730, 698, 676 cm^{-1}. HRMS (ESI) calcd for $C_{34}H_{36}NOSi$ $[M+H]^+$: 502.2561, found 502.2562.

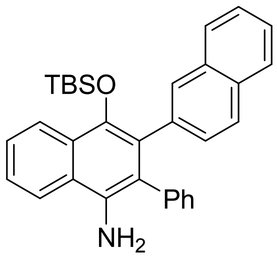

2r

1-((*tert*-Butyldimethylsilyl)oxy)-3-phenyl-[2,2'-binaphthalen]-4-amine (2r). (0.3 mmol scale, $Ni(acac)_2 \cdot 2H_2O$ (0.03 mmol, 8.8 mg), P(*p*-$CF_3C_6H_4)_3$ (0.03 mmol, 14.0 mg), Cs_2CO_3 (0.06 mmol, 19.5 mg), naphthalen-2-ylboronic acid (0.6 mmol, 103.2 mg), 1,4-dioxane (2.0 mL) and *o*-(cyano)phenyl propargyl ether **1a** (104.3 mg, 0.3 mmol) were stirred at 90 °C for 6 h. Purification of the crude product by column chromatography on silica gel (eluent: petroleum ether: ethyl acetate = 13:1) afforded the title product in 70% yield (100.5 mg) as a light yellow solid. M.p. = 160-161 °C. ^1H NMR(400 MHz, C_6D_6, Me_4Si): δ -0.46 (bs, 3H), -0.31 (bs, 3H), 0.93 (s, 9H), 3.59 (s, 2H), 6.81-7.01 (m, 3H), 6.83 (t, *J* = 6.8 Hz, 1H), 7.13-7.21 (m, 3H), 7.27-7.33 (m, 2H), 7.41-7.47 (m, 2H), 7.51 (d, *J* = 8.0 Hz, 1H), 7.58-7.61 (m, 2H), 7.83 (s, 1H), 8.53 (d, *J* = 8.4 Hz, 1H). ^{13}C NMR(100 MHz, C_6D_6, Me_4Si): δ -3.72, 18.72, 26.33, 122.02, 123.08, 124.54, 124.95, 125.48, 125.69, 125.88, 125.96, 126.64, 126.92, 127.83, 128.17, 128.61 (br), 128.84, 128.99, 131.04, 131.74 (br), 132.14, 132.24, 133.45, 134.84, 136.36, 139.24, 141.36. IR (neat): 3455, 3374, 3078, 3056, 3023, 2949, 2928, 2893, 2855, 1607, 1579, 1493, 1468, 1443, 1414, 1375, 1338, 1257, 1219, 1200, 1170, 1142, 1125, 1070, 1029, 957, 911, 884, 855, 839, 825, 783, 760, 744, 701, 673, 631 cm^{-1}. HRMS (ESI) calcd for $C_{32}H_{34}NOSi$ $[M+H]^+$: 476.2404, found 476.2403.

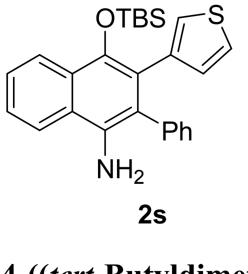

2s

4-((*tert*-Butyldimethylsilyl)oxy)-2-phenyl-3-(thiophen-3-yl)naphthalen-1-amine (2s). (0.3 mmol scale, $Ni(acac)_2 \cdot 2H_2O$ (0.03 mmol, 8.8 mg), P(*p*-$CF_3C_6H_4)_3$ (0.03 mmol, 14.0

mg), Cs_2CO_3 (0.06 mmol, 19.5 mg), thiophen-3-ylboronic acid (0.6 mmol, 76.8 mg), 1,4-dioxane (2.0 mL) and *o*-(cyano)phenyl propargyl ether **1a** (104.3 mg, 0.3 mmol) were stirred at 90 °C for 10 h. Purification of the crude product by column chromatography on silica gel (eluent: petroleum ether: ethyl acetate = 10:1) afforded the title product in 35% yield (44.9 mg) as a light yellow solid. M.p. = 177-178 °C. ^{1}H NMR(400 MHz, $CDCl_3$, Me_4Si): δ -0.33 (s, 6H), 0.98 (s, 9H), 3.85 (bs, 2H), 6.77 (d, *J* = 4.8 Hz, 1H), 6.84 (dd, *J* = 2.8, 0.8 Hz, 1H), 6.98 (dd, *J* = 4.8, 3.2 Hz, 1H), 7.12-7.14 (m, 2H), 7.23-7.25 (m, 1H), 7.29 (t, *J* = 7.6 Hz, 2H), 7.47-7.52 (m, 2H), 7.83-7.86 (m, 1H), 8.18-8.21 (m, 1H). ^{13}C NMR(100 MHz, $CDCl_3$, Me_4Si): δ -4.46, 18.54, 26.15, 121.12, 122.58, 123.30, 123.51, 124.12, 124.19, 125.09, 125.49, 126.82, 128.13, 128.41, 130.79, 131.25, 133.40, 137.94, 138.78, 141.50. IR (neat): 3463, 3376, 3080, 3054, 2951, 2928, 2888, 2855, 1741, 1611, 1575, 1491, 1467, 1440, 1374, 1256, 1206, 1162, 1072, 1005, 943, 895, 844, 826, 765, 706, 669 cm^{-1}. HRMS (ESI) calcd for $C_{26}H_{30}NOSSi$ [M+H]$^+$: 432.1812, found 432.1813.

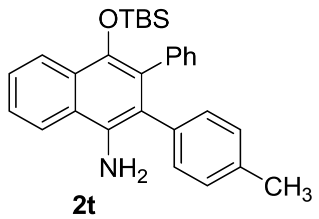

2t

4-((*tert*-Butyldimethylsilyl)oxy)-3-phenyl-2-(*p*-tolyl)naphthalen-1-amine (2t). (0.3 mmol scale, $Ni(acac)_2{\cdot}2H_2O$ (0.03 mmol, 8.8 mg), P(*p*-$CF_3C_6H_4$)$_3$ (0.03 mmol, 14.0 mg), Cs_2CO_3 (0.06 mmol, 19.5 mg), phenylboronic acid (0.6 mmol, 73.2 mg), 1,4-dioxane (2.0 mL) and *o*-(cyano)phenyl propargyl ether **1b** (108.5 mg, 0.3 mmol) were stirred at 90 °C for 5 h. Purification of the crude product by column chromatography on silica gel (eluent: petroleum ether: ethyl acetate = 15:1) afforded the title product in 76% yield (100.3 mg) as a light yellow solid. M.p. = 189-191 °C. ^{1}H NMR(400 MHz, C_6D_6, Me_4Si): δ -0.25 (s, 6H), 1.03 (s, 9H), 2.01 (s, 3H), 3.60 (s, 2H), 6.86 (d, *J* = 7.6 Hz, 2H), 6.93 (t, *J* = 7.6 Hz, 1H), 7.01-7.05 (m, 4H), 7.27-7.33 (m, 3H), 7.44 (t, *J* = 7.2 Hz, 1H), 7.60 (d, *J* = 8.4 Hz, 1H), 8.50 (d, *J* = 8.8 Hz, 1H). ^{13}C NMR(100 MHz, C_6D_6, Me_4Si): δ -3.69, 18.89, 21.15, 26.48, 122.00, 123.16, 124.52, 124.91, 125.28, 125.53, 126.31, 127.44, 128.85, 129.20, 129.39, 131.56, 132.97, 134.93, 136.15, 136.36, 139.11, 141.15. IR (neat): 3458, 3375, 3078, 3057,

3026, 2951, 2928, 2890, 2856, 1609, 1581, 1512, 1495, 1467, 1439, 1374, 1309, 1256, 1178, 1142, 1072, 1026, 899, 832, 785, 758, 702, 671 cm^{-1}. HRMS (ESI) calcd for $C_{29}H_{34}NOSi$ $[M+H]^+$: 440.2404, found 440.2409.

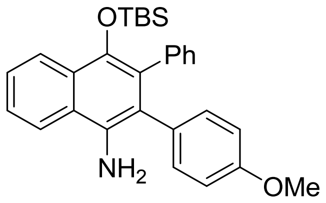

2u

4-((*tert*-Butyldimethylsilyl)oxy)-2-(4-methoxyphenyl)-3-phenylnaphthalen-1-amine (2u). (0.3 mmol scale, $Ni(acac)_2 \cdot 2H_2O$ (0.03 mmol, 8.8 mg), $P(p\text{-}CF_3C_6H_4)_3$ (0.03 mmol, 14.0 mg), Cs_2CO_3 (0.06 mmol, 19.5 mg), phenylboronic acid (0.6 mmol, 73.2 mg), 1,4-dioxane (2.0 mL) and *o*-(cyano)phenyl propargyl ether **1c** (113.3 mg, 0.3 mmol) were stirred at 90 °C for 4 h. Purification of the crude product by column chromatography on silica gel (eluent: petroleum ether: ethyl acetate = 8:1) afforded the title product in 69% yield (94.8 mg) as a yellow solid. M.p. = 216-217 °C. ^1H NMR(400 MHz, C_6D_6, Me_4Si): δ -0.25 (s, 6H), 1.03 (s, 9H), 3.20 (s, 3H), 3.60 (s, 2H), 6.64 (d, J = 8.8 Hz, 2H), 6.94 (t, J = 7.2 Hz, 1H), 7.00-7.06 (m, 4H), 7.28-7.34 (m, 3H), 7.45 (t, J = 7.6 Hz, 1H), 7.62 (d, J = 8.0 Hz, 1H), 8.51 (d, J = 8.4 Hz, 1H). ^{13}C NMR(100 MHz, C_6D_6, Me_4Si): δ -3.72, 18.89, 26.46, 54.56, 114.14, 121.98, 122.88, 124.53, 124.88, 125.28, 125.54, 126.29, 127.48, 128.85, 129.38, 131.23, 132.71, 132.97, 135.18, 139.15, 141.13, 158.72. IR (neat): 3459, 3377, 3076, 3024, 2999, 2951, 2929, 2890, 2854, 1741, 1605, 1513, 1495, 1464, 1437, 1370, 1305, 1284, 1251, 1172, 1146, 1072, 1033, 1005, 988, 926, 898, 876, 833, 784, 760, 703, 673, 654 cm^{-1}. HRMS (ESI) calcd for $C_{29}H_{34}NO_2Si$ $[M+H]^+$: 456.2353, found 456.2356.

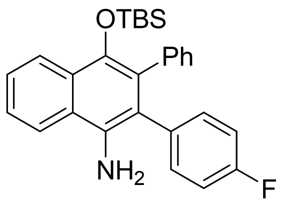

2v

4-((*tert*-Butyldimethylsilyl)oxy)-2-(4-fluorophenyl)-3-phenylnaphthalen-1-amine (2v). (0.3 mmol scale, Ni(acac)$_2$·2H$_2$O (0.03 mmol, 8.8 mg), P(*p*-CF$_3$C$_6$H$_4$)$_3$ (0.03 mmol, 14.0 mg), Cs$_2$CO$_3$ (0.06 mmol, 19.5 mg), phenylboronic acid (0.6 mmol, 73.2 mg), 1,4-dioxane (2.0 mL) and *o*-(cyano)phenyl propargyl ether **1d** (109.7 mg, 0.3 mmol) were stirred at 90 ^{o}C for 4 h. Purification of the crude product by column chromatography on silica gel (eluent: petroleum ether: ethyl acetate = 20:1) afforded the title product in 76% yield (101.5 mg) as a yellow solid. M.p. = 173-174 ^{o}C. ^{1}H NMR(400 MHz, C$_6$D$_6$, Me$_4$Si): δ -0.25 (s, 6H), 1.03 (s, 9H), 3.47 (bs, 2H), 6.67 (t, *J* = 8.4 Hz, 2H), 6.85-6.88 (m, 2H), 6.92-6.96 (m, 1H), 7.01 (t, *J* = 6.8 Hz, 2H), 7.16-7.18 (m, 2H), 7.32 (t, *J* = 8.4 Hz, 1H), 7.45 (td, *J* = 6.8, 1.2 Hz, 1H), 7.60 (d, *J* = 8.4 Hz, 1H), 8.50 (d, *J* = 8.4 Hz, 1H). ^{13}C NMR(100 MHz, C$_6$D$_6$, Me$_4$Si): δ -3.72, 18.87, 26.43, 115.49 (d, *J* = 21.3 Hz), 121.89, 121.90, 124.55, 124.79, 125.51, 125.68, 126.44, 127.51, 128.97, 129.01, 132.84, 133.29 (d, *J* = 7.6 Hz), 134.83 (d, *J* = 0.8 Hz), 135.08 (d, *J* = 3.5 Hz), 138.75, 141.18, 161.94 (d, *J* = 245.7 Hz). IR (neat): 3463, 3377, 3073, 2953, 2930, 2890, 2857, 1953, 1903, 1731, 1697, 1606, 1506, 1467, 1435, 1371, 1292, 1255, 1221, 1176, 1153, 1071, 1031, 941, 899, 840, 781, 759, 701, 672, 650 cm^{-1}. HRMS (ESI) calcd for C$_{28}$H$_{31}$FNOSi [M+H]$^{+}$: 444.2153, found 444.2159.

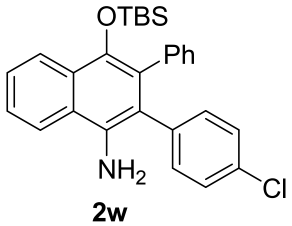

2w

4-((*tert*-Butyldimethylsilyl)oxy)-2-(4-chlorophenyl)-3-phenylnaphthalen-1-amine (2w). (0.3 mmol scale, Ni(acac)$_2$·2H$_2$O (0.03 mmol, 8.8 mg), P(*p*-CF$_3$C$_6$H$_4$)$_3$ (0.03 mmol, 14.0 mg), Cs$_2$CO$_3$ (0.06 mmol, 19.5 mg), phenylboronic acid (0.6 mmol, 73.2 mg), 1,4-dioxane (2.0 mL) and *o*-(cyano)phenyl propargyl ether **1e** (114.6 mg, 0.3 mmol) were stirred at 90 ^{o}C for 4 h. Purification of the crude product by column chromatography on silica gel which was treated with petroleum ether/Et$_3$N = 1:1 and then petroleum ether before loading the sample (eluent: petroleum ether: ethyl acetate = 20:1) afforded the title product in 61% yield (83.6 mg) as a light yellow solid. M.p. = 189-190 ^{o}C. ^{1}H NMR(400 MHz, C$_6$D$_6$, Me$_4$Si): δ -0.28 (s, 6H), 1.00 (s, 9H), 3.46 (bs, 2H), 6.80 (d, *J* = 8.0 Hz, 2H), 6.90 (t, *J* =

7.2 Hz, 1H), 6.94-6.99 (m, 4H), 7.14 (d, J = 7.2 Hz, 2H), 7.30 (t, J = 7.2 Hz, 1H), 7.43 (t, J = 7.2 Hz, 1H), 7.58 (d, J = 8.4 Hz, 1H), 8.46 (d, J = 8.4 Hz, 1H). ^{13}C NMR(100 MHz, C_6D_6, Me_4Si): δ -3.73, 18.86, 26.43, 121.61, 121.91, 124.53, 124.75, 125.59, 125.71, 126.55, 127.55, 128.74, 128.80, 128.99, 132.80, 132.82, 133.05, 134.68, 137.74, 138.55, 141.18. IR (neat): 3457, 3377, 3277, 3079, 3060, 3026, 2927, 2886, 2855, 1952, 1906, 1605, 1492, 1466, 1437, 1369, 1307, 1256, 1223, 1181, 1144, 1071, 1029, 1009, 896, 832, 786, 759, 699, 668 cm^{-1}. HRMS (ESI) calcd for $C_{28}H_{31}ClNOSi$ $[M+H]^+$: 460.1858, found 460.1862.

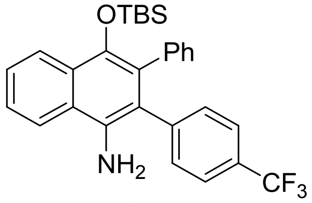

2x

4-((*tert*-Butyldimethylsilyl)oxy)-3-phenyl-2-(4-(trifluoromethyl)phenyl)naphthalen-1-amine (2x). (0.3 mmol scale, $Ni(acac)_2{\cdot}2H_2O$ (0.03 mmol, 8.8 mg), P(p-$CF_3C_6H_4)_3$ (0.03 mmol, 14.0 mg), Cs_2CO_3 (0.06 mmol, 19.5 mg), phenylboronic acid (0.6 mmol, 73.2 mg), 1,4-dioxane (2.0 mL) and o-(cyano)phenyl propargyl ether **1f** (124.7 mg, 0.3 mmol) were stirred at 90 °C for 5.5 h. Purification of the crude product by column chromatography on silica gel (eluent: petroleum ether: ethyl acetate = 20:1) afforded the title product in 74% yield (109.0 mg) as a yellow solid. M.p. = 129-130 °C. ^1H NMR(400 MHz, C_6D_6, Me_4Si): δ -0.28 (s, 6H), 1.01 (s, 9H), 3.38 (s, 2H), 6.87 (t, J = 7.2 Hz, 1H), 6.92-6.96 (m, 4H), 7.09 (d, J = 7.2 Hz, 2H), 7.18 (t, J = 8.0 Hz, 2H), 7.31 (t, J = 7.2 Hz, 1H), 7.44 (t, J = 7.2 Hz, 1H), 7.56 (d, J = 8.0 Hz, 1H), 8.48 (d, J = 8.4 Hz, 1H). ^{13}C NMR(100 MHz, C_6D_6, Me_4Si): δ -3.75, 18.86, 26.40, 121.43, 121.84, 124.58, 124.75, 124.93 (q, J = 272 Hz), 125.39 (q, J = 3.8 Hz), 125.78, 125.84, 126.68, 127.54, 128.50, 128.84 (q, J = 32.4 Hz), 129.15, 132.10, 132.75, 134.45, 138.30, 141.31, 143.36 (q, J = 1.1 Hz). IR (neat): 3459, 3376, 3066, 3035, 2952, 2931, 2893, 2858, 1931, 1711, 1613, 1583, 1496, 1467, 1441, 1374, 1322, 1255, 1158, 1125, 1067, 1016, 900, 839, 782, 758, 725, 701, 668, 637 cm^{-1}. HRMS (ESI) calcd for $C_{29}H_{31}F_3NOSi$ $[M+H]^+$: 494.2122, found 494.2121.

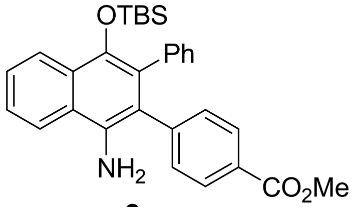

2y

Methyl 4-(1-amino-4-((*tert*-butyldimethylsilyl)oxy)-3-phenylnaphthalen-2-yl)benzoate (2y). (0.3 mmol scale, Ni(acac)$_2$·2H_2O (0.03 mmol, 8.8 mg), P(*p*-$CF_3C_6H_4$)$_3$ (0.03 mmol, 14.0 mg), Cs_2CO_3 (0.06 mmol, 19.5 mg), phenylboronic acid (0.6 mmol, 73.2 mg), 1,4-dioxane (2.0 mL) and *o*-(cyano)phenyl propargyl ether **1g** (121.7 mg, 0.3 mmol) were stirred at 90 ^oC for 4.5 h. Purification of the crude product by column chromatography on silica gel (eluent: petroleum ether: ethyl acetate = 10:1) afforded the title product in 62% yield (89.3 mg) as a yellow solid. M.p. = 189-190 ^oC. ^1H NMR(400 MHz, C_6D_6, Me_4Si): δ -0.27 (s, 6H), 1.01 (s, 9H), 3.43 (s, 3H), 3.51 (bs, 2H), 6.88 (t, *J* = 7.2 Hz, 1H), 6.96 (t, *J* = 6.8 Hz, 2H), 7.04 (d, *J* = 8.4 Hz, 2H), 7.15-7.17 (m, 2H), 7.32 (t, *J* = 8.4 Hz, 1H), 7.44 (t, *J* = 8.0 Hz, 1H), 7.61 (d, *J* = 8.0 Hz, 1H), 7.95 (d, *J* = 8.4 Hz, 2H), 8.48 (d, *J* = 8.4 Hz, 1H). ^{13}C NMR(100 MHz, C_6D_6, Me_4Si): δ -3.72, 18.86, 26.42, 51.53, 121.95, 122.02, 124.55, 124.82, 125.65, 125.74, 126.59, 127.55, 128.59, 128.99, 129.09, 129.92, 131.78, 132.80, 134.51, 138.53, 141.22, 144.44, 166.47. IR (neat): 3460, 3380, 3075, 3035, 2950, 2927, 2886, 2854, 1936, 1724, 1605, 1495, 1465, 1437, 1371, 1276, 1186, 1143, 1105, 1071, 1017, 900, 829, 783, 759, 698, 668 cm^{-1}. HRMS (ESI) calcd for $C_{30}H_{34}NO_3Si$ $[M+H]^+$: 484.2302, found 484.2302.

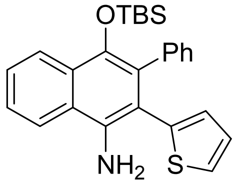

2z

4-((*tert*-Butyldimethylsilyl)oxy)-3-phenyl-2-(thiophen-2-yl)naphthalen-1-amine (2z). (0.3 mmol scale, Ni(acac)$_2$·2H_2O (0.03 mmol, 8.8 mg), P(*p*-$CF_3C_6H_4$)$_3$ (0.03 mmol, 14.0 mg), Cs_2CO_3 (0.06 mmol, 19.5 mg), phenylboronic acid (0.6 mmol, 73.2 mg), 1,4-dioxane (2.0 mL) and *o*-(cyano)phenyl propargyl ether **1h** (106.1 mg, 0.3 mmol) were stirred at 90 ^oC for 4 h. Purification of the crude product by column chromatography on silica gel

(eluent: petroleum ether: ethyl acetate = 25:1) afforded the title product in 80% yield (103.1 mg) as a brown solid. M.p. = 197-198 °C. ^{1}H NMR(400 MHz, C_6D_6, Me_4Si): δ -0.27 (s, 6H), 1.00 (s, 9H), 3.78 (s, 2H), 6.66 (dd, J = 5.2, 3.6 Hz,1H), 6.73- 6.74 (m, 1H), 6.85 (dd, J = 5.0, 0.8 Hz, 1H), 6.99 (t, J = 7.2 Hz, 1H), 7.08 (t, J = 7.2 Hz, 2H), 7.27 (td, J = 8.2, 1.6 Hz, 1H), 7.33 (d, J = 7.4 Hz, 2H), 7.42 (t, J = 7.4 Hz, 1H), 7.51 (d, J = 8.8 Hz, 1H), 8.45 (d, J = 8.4 Hz, 1H). ^{13}C NMR(100 MHz, C_6D_6, Me_4Si): δ -3.69, 18.86, 26.43, 114.74, 122.10, 124.52, 125.64, 125.86, 126.63, 126.89, 126.91, 127.52, 129.25, 129.48, 129.74, 132.46, 137.02, 138.95, 140.41, 140.95. IR (neat): 3446, 3367, 3103, 3075, 3024, 2949, 2928, 2888, 2855, 1608, 1495, 1466, 1435, 1372, 1256, 1224, 1207, 1171, 1132, 1068, 1029, 1005, 939, 884, 836, 785, 759, 732, 693 cm^{-1}. HRMS (ESI) calcd for $C_{26}H_{30}NOSSi$ $[M+H]^+$: 432.1812, found 432.1816.

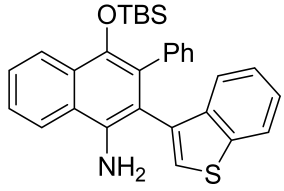

2za

2-(Benzo[*b*]thiophen-3-yl)-4-((*tert*-butyldimethylsilyl)oxy)-3-phenylnaphthalen-1-amine (2za). (0.3 mmol scale, $Ni(acac)_2 \cdot 2H_2O$ (0.03 mmol, 8.8 mg), P(*p*-$CF_3C_6H_4)_3$ (0.03 mmol, 14.0 mg), Cs_2CO_3 (0.06 mmol, 19.5 mg), phenylboronic acid (0.6 mmol, 73.2 mg), 1,4-dioxane (2.0 mL) and *o*-(cyano)phenyl propargyl ether **1i** (121.1 mg, 0.3 mmol) were stirred at 90 °C for 4 h. Purification of the crude product by column chromatography on silica gel (eluent: petroleum ether: ethyl acetate = 30:1) afforded the title product in 74% yield (107.3 mg) as a yellow solid. M.p. = 165-166 °C. ^{1}H NMR(400 MHz, C_6D_6, Me_4Si): δ -0.32 (s, 3H), -0.20 (s, 3H), 1.02 (s, 9H), 3.45 (s, 2H), 6.71 (s, 1H), 6.82 (t, J = 7.2 Hz, 2H), 7.00-7.09 (m, 4H), 7.30 (t, J = 7.8 Hz, 2H), 7.44-7.56 (m, 4H), 8.54 (d, J = 8.0 Hz, 1H). ^{13}C NMR(100 MHz, C_6D_6, Me_4Si): δ -3.85, -3.57, 18.88, 26.45, 115.92, 121.96, 122.88, 123.83, 124.49, 124.59, 124.64, 124.67, 125.62, 125.70, 126.53, 126.94, 127.36, 129.34, 129.94, 131.83 (br), 134.63, 136.33, 138.87, 139.48, 140.17, 141.01. IR (neat): 3456, 3370, 3107, 3061, 2955, 2928, 2888, 2855, 1729, 1613, 1581, 1495, 1465, 1432,

1373, 1313, 1282, 1253, 1215, 1169, 1113, 1070, 1029, 1010, 986, 939, 894, 833, 781, 756, 699, 668 cm^{-1}. HRMS (ESI) calcd for $C_{30}H_{32}NOSSi$ $[M+H]^+$: 482.1968, found 482.1970.

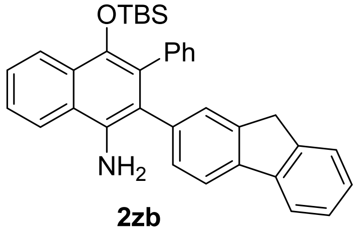

2zb

4-((*tert*-Butyldimethylsilyl)oxy)-2-(9*H*-fluoren-2-yl)-3-phenylnaphthalen-1-amine (2zb). (0.3 mmol scale, $Ni(acac)_2 \cdot 2H_2O$ (0.03 mmol, 8.8 mg), $P(p\text{-}CF_3C_6H_4)_3$ (0.03 mmol, 14.0 mg), Cs_2CO_3 (0.06 mmol, 19.5 mg), phenylboronic acid (0.6 mmol, 73.2 mg), 1,4-dioxane (2.0 mL) and *o*-(cyano)phenyl propargyl ether **1j** (130.7 mg, 0.3 mmol) were stirred at 90 °C for 4 h. Purification of the crude product by column chromatography on silica gel (eluent: petroleum ether: ethyl acetate = 30:1) afforded the title product in 70% yield (108.2 mg) as a yellow solid. M.p. = 230-231 °C. ^1H NMR(400 MHz, C_6D_6, Me_4Si): δ -0.23 (s, 6H), 1.04 (s, 9H), 3.37 (dd, J = 35.2, 22 Hz, 2H), 3.67 (bs, 2H), 6.87 (t, J = 7.2 Hz, 1H), 6.98 (t, J = 7.6 Hz, 2H), 7.10-7.16 (m, 2H), 7.18-7.22 (m, 3H), 7.32-7.36 (m, 3H), 7.45-7.49 (m, 2H), 7.54 (d, J = 7.2 Hz, 1H), 7.66 (d, J = 8.8 Hz, 1H), 8.54 (d, J = 8.4 Hz, 1H). ^{13}C NMR(100 MHz, C_6D_6, Me_4Si): δ -3.70, -3.68, 18.90, 26.48, 36.85, 120.11, 120.22, 122.01, 123.50, 124.60, 124.95, 125.26, 125.38, 125.63, 126.39, 126.91, 127.04, 127.47, 128.30, 128.94, 129.21, 130.35, 132.97, 134.98, 137.81, 139.05, 140.57, 141.24, 141.89, 143.70, 143.72. IR (neat): 3659, 3645, 3435, 3360, 3071, 3043, 2929, 2856, 1954, 1901, 1806, 1741, 1608, 1494, 1464, 1372, 1307, 1254, 1192, 1133, 1070, 1030, 1004, 983, 931, 836, 785, 759, 738, 697, 637 cm^{-1}. HRMS (ESI) calcd for $C_{35}H_{36}NOSi$ $[M+H]^+$: 514.2561, found 514.2557.

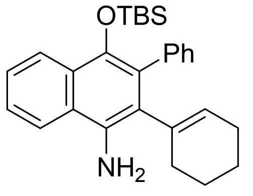

2zc

4-(*tert*-Butyldimethylsilyloxy)-2-cyclohexenyl-3-phenylnaphthalen-1-amine (2zc). (0.3 mmol scale, $Ni(acac)_2 \cdot 2H_2O$ (0.03 mmol, 8.8 mg), $P(p\text{-}CF_3C_6H_4)_3$ (0.03 mmol, 14.0 mg), Cs_2CO_3 (0.06 mmol, 19.5 mg), phenylboronic acid (0.6 mmol, 73.2 mg), 1,4-dioxane (2.0 mL) and *o*-(cyano)phenyl propargyl ether **1k** (105.5 mg, 0.3 mmol) were stirred at 90 °C for 8 h. Purification of the crude product by thin layer chromatography on silica gel which was embellished with triethylamine first (eluent: petroleum ether: ethyl acetate = 25:1) afforded the title product in 35% yield (45.3 mg) as a light yellow solid. M.p. = 91-92 °C. ^{1}H NMR(400 MHz, C_6D_6, Me_4Si): δ -0.39 (s, 3H), -0.08 (s, 3H), 1.01 (s, 9H), 1.12-1.18 (m, 1H), 1.32-1.40 (m, 3H), 1.50-1.55 (m, 1H), 1.85-1.97 (m, 3H), 3.75 (bs, 2H), 5.76 (s, 1H), 7.12 (t, J = 7.6 Hz, 1H), 7.21 (t, J = 7.6 Hz, 2H), 7.33 (t, J = 8.0 Hz, 1H), 7.42 (t, J = 7.6 Hz, 1H), 7.51 (d, J = 7.2 Hz, 2H), 7.70 (d, J = 8.4 Hz, 1H), 8.45 (d, J = 8.4 Hz, 1H). ^{13}C NMR(100 MHz, C_6D_6, Me_4Si): δ -3.94, -3.41, 18.87, 22.37, 23.26, 25.80, 26.44, 29.47, 122.14, 124.44, 124.96, 125.11, 125.33, 125.49, 126.68, 127.26 (br), 130.05, 132.87 (br), 133.57, 136.95, 139.09, 141.06. IR (film): 3439, 3358, 3059, 3031, 2926, 2892, 2855, 1946, 1600, 1494, 1465, 1436, 1412, 1374, 1309, 1252, 1220, 1171, 1137, 1069, 1032, 1006, 957, 924, 893, 843, 785, 759, 695, 669 cm^{-1}. HRMS (ESI) calcd for $C_{28}H_{36}NOSi$ [M+H]$^+$: 430.2561, found 430.2562.

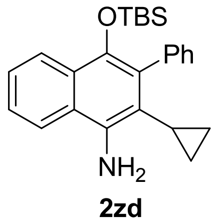

2zd

4-((*tert*-Butyldimethylsilyl)oxy)-2-cyclopropyl-3-phenylnaphthalen-1-amine (2zd). (0.3 mmol scale, $Ni(acac)_2 \cdot 2H_2O$ (0.03 mmol, 8.8 mg), $P(p\text{-}CF_3C_6H_4)_3$ (0.03 mmol, 14.0 mg), Cs_2CO_3 (0.06 mmol, 19.5 mg), phenylboronic acid (0.6 mmol, 73.2 mg), 1,4-dioxane (2.0 mL) and *o*-(cyano)phenyl propargyl ether **1l** (93.4 mg, 0.3 mmol) were stirred at 90 °C for 6 h. Purification of the crude product by preparative TLC on silica gel which was treated with Et_3N before loading the sample (eluent: petroleum ether: ethyl acetate = 10:1) afforded the title product in 23% yield (26.4 mg) as a light yellow solid. M.p. = 155-156 °C. ^{1}H NMR(400 MHz, C_6D_6, Me_4Si): δ -0.29 (s, 6H), 0.06-0.10 (m, 2H), 0.43-0.45 (m, 2H),

1.04 (s, 9H), 1.42-1.49 (m, 1H), 4.00 (bs, 2H), 7.16-7.18 (m, 1H), 7.26 (t, J = 7.2 Hz, 2H), 7.32 (t, J = 7.2 Hz, 1H), 7.39-7.44 (m, 3H), 7.65 (d, J = 8.4 Hz, 1H), 8.43 (d, J = 8.4 Hz, 1H). ^{13}C NMR(100 MHz, C_6D_6, Me_4Si): δ -3.74, 9.57, 11.80, 18.87, 26.49, 119.56, 121.26, 124.50, 124.53, 124.83, 125.21, 126.49, 127.61, 128.35, 130.79, 132.60, 137.83, 139.61, 140.56. IR (neat): 3451, 3375, 3075, 3037, 2995, 2952, 2928, 2888, 2855, 1949, 1805, 1717, 1607, 1496, 1466, 1375, 1311, 1255, 1220, 1193, 1166, 1125, 1075, 1029, 1006, 954, 906, 874, 827, 782, 755, 698, 655 cm^{-1}. HRMS (ESI) calcd for $C_{25}H_{32}NOSi$ [M+H]$^+$: 390.2248, found 390.2249.

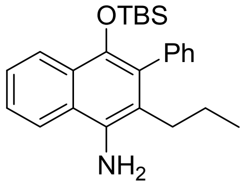

2ze

4-((*tert*-Butyldimethylsilyl)oxy)-3-phenyl-2-propylnaphthalen-1-amine (2ze). (0.3 mmol scale, Ni(acac)$_2$·2H_2O (0.03 mmol, 8.8 mg), P(p-$CF_3C_6H_4$)$_3$ (0.03 mmol, 14.0 mg), Cs_2CO_3 (0.06 mmol, 19.5 mg), phenylboronic acid (0.6 mmol, 73.2 mg), 1,4-dioxane (2.0 mL) and *o*-(cyano)phenyl propargyl ether **1m** (94.1 mg, 0.3 mmol) were stirred at 90 ^{o}C for 3 h. Purification of the crude product by preparative TLC on silica gel which was treated with Et_3N before loading the sample (eluent: petroleum ether: ethyl acetate = 8:1) afforded the title product in 29% yield (34.6 mg) as a yellow oil. ^{1}H NMR(400 MHz, C_6D_6, Me_4Si): δ -0.21 (s, 6H), 0.70 (t, J = 7.6 Hz, 3H), 0.97 (s, 9H), 1.41-1.51 (m, 2H), 2.43-2.47 (m, 2H), 3.13 (bs, 2H), 7.13-7.16 (m, 1H), 7.23 (t, J = 7.2 Hz, 2H), 7.30-7.34 (m, 1H), 7.36-7.41 (m, 3H), 7.65 (d, J = 8.0 Hz, 1H), 8.42 (d, J = 8.4 Hz, 1H). ^{13}C NMR(100 MHz, C_6D_6, Me_4Si): δ -3.41, 14.52, 18.91, 22.40, 26.43, 31.58, 121.24, 121.31, 124.49, 124.51, 125.03, 125.27, 127.01, 127.70, 127.97, 130.19, 132.26, 134.56, 139.50, 141.29. IR (film): 3466, 3381, 3062, 2955, 2862, 1714, 1620, 1459, 1375, 1256, 1217, 1167, 1090, 1022, 952, 836, 769, 702 cm^{-1}. HRMS (ESI) calcd for $C_{25}H_{34}NOSi$ [M+H]$^+$: 392.2404, found 392.2407.

Mechanistic studies.

Formation of IPrNi(acac) 3 using ^{t}BuOK as the base.

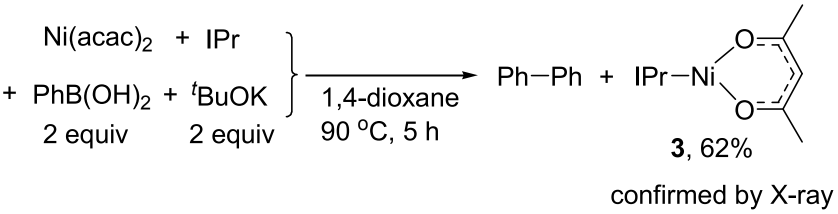

In a nitrogen-filled glovebox, $Ni(acac)_2$ (0.4 mmol, 102.8 mg), IPr (0.4 mmol, 155.4 mg), *t*-KOBu (0.8 mmol, 89.8 mg) and phenylboronic acid (0.8 mmol, 97.5 mg) were added to an oven-dried sealable tube, then 1,4-dioxane (10.0 mL) was added. The tube cap was then securely fitted and sealed with electrical tape before it was removed from the glovebox. Then the sealed tube was taken outside the glovebox and stirred at 90 °C in an oil-bath for 5 h. After the reaction mixture was cooled down to room temperature, then it was taken into the glovebox, filtered through a celite pad and washed with minimum amount of toluene. The solvent was evaporated under the reduced pressure to afford a dark red solid. The crude product was further purified by recrystallization from hexane at -30 °C to give the complex **3** in 62% yield (136.3 mg) as a red crystal.

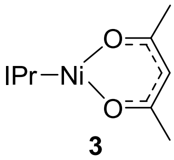

3

IPrNi(acac) **3.** ^{1}H NMR(400 MHz, C_6D_6, Me_4Si): δ -16.06 (br), -10.61 (br), 1.21 (d), 1.34 (d), 1.91 (br), 5.03 (br), 6.31 (br), 14.37 (br). HRMS (MALDI-FT-DHB) calcd for $C_{32}H_{43}O_2N_2Ni$ $[M]^+$: 545.2673, found 545.2683. Anal. calcd for $C_{32}H_{43}O_2N_2Ni$: C 70.34%, H 7.93%, N 5.13%; Found: C 70.30%, H 7.63%, N 5.38%.

In another experiment, biphenyl was isolated in 30% yield. The procedure was shown as follows:

In a nitrogen-filled glovebox, $Ni(acac)_2$ (0.3 mmol, 77.1 mg), IPr (0.3 mmol, 116.6 mg), *t*-KOBu (0.6 mmol, 67.3 mg) and phenylboronic acid (0.6 mmol, 73.2 mg) were added to an oven-dried sealable tube, then 1,4-dioxane (10.0 mL) was added. The tube cap

was then securely fitted and sealed with electrical tape before it was removed from the glovebox. Then the sealed tube was taken outside the glovebox and stirred at 90 °C in an oil-bath for 5 h. After the reaction mixture was cooled down to room temperature, it was filtered through a short silica gel column and washed with ethyl acetate. The solvent was evaporated under the reduced pressure and the residue was purified by preparative TLC on silica gel (eluent: petroleum ether: ethyl acetate = 50:1) to afford the biphenyl in 30% yield (13.7 mg) as a white solid.

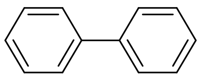

1,1'-Biphenyl. ^{1}H NMR(400 MHz, $CDCl_3$, Me_4Si): δ 7.35-7.38 (m, 2H), 7.46 (t, *J* = 7.6 Hz, 4H), 7.62 (d, *J* = 7.2 Hz, 4H) . ^{13}C NMR(100 MHz, $CDCl_3$, Me_4Si): δ 127.15, 127.23, 128.73, 141.21. The NMR data is in agreement with that previously reported. [2]

Formation of IPrNi(acac) 3 using Cs_2CO_3 as the base.

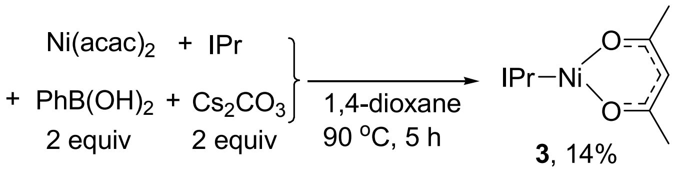

In a nitrogen-filled glovebox, $Ni(acac)_2$ (0.3 mmol, 77.1 mg), IPr (0.3 mmol, 116.6 mg), Cs_2CO_3 (0.6 mmol, 195.5 mg) and phenylboronic acid (0.6 mmol, 73.2 mg) were added to an oven-dried sealable tube, then 1,4-dioxane (10.0 mL) was added. The tube cap was then securely fitted and sealed with electrical tape before it was removed from the glovebox. Then the sealed tube was taken outside the glovebox and stirred at 90 °C in an oil-bath for 5 h. In this case, a large amount of precipitate could be observed during the reaction process. After the reaction mixture was cooled down to room temperature, the tube was taken into the glovebox, filtered through a celite pad and washed with minimum amount of toluene. The solvent was evaporated under the reduced pressure to afford an orange red solid. The crude product was further purified by recrystallization from hexane at -30 °C to give the complex **3** in 14% yield (23.0 mg) as a red crystal. The ^{1}H NMR of this compound is consistent with that reported by the above procedure using ^{t}BuOK as the

base. Furthermore, the structure of the complex **3** obtained by this method was aslo confirmed by X-ray crystallographic analysis.

Formation of IPrNi(acac) 3 through comproportionation reaction.

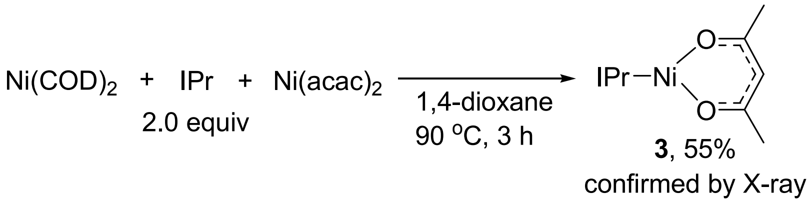

In a nitrogen-filled glovebox, $Ni(cod)_2$ (0.2 mmol, 55.0 mg), $Ni(acac)_2$ (0.2 mmol, 51.4 mg) and IPr (0.4 mmol, 155.4 mg) were added to an oven-dried sealable tube, then 1,4-dioxane (10.0 mL) was added. The tube cap was then securely fitted and sealed with electrical tape before it was removed from the glovebox. Then the sealed tube was taken outside the glovebox and stirred at 90 ^{o}C in an oil-bath for 3 h. After the reaction mixture was cooled down to room temperature, it was taken into the glovebox, filtered through a celite pad and washed with minimum amount of toluene. The solvent was evaporated under the reduced pressure to afford a dark red solid. The crude product was further purified by recrystallization from hexane at -30 ^{o}C to give the complex **3** in 55% yield (119.9 mg) as a red crystal. The ^{1}H NMR of this compound is consistent with that reported by the above procedure using $^{t}BuOK$ as the base. Furthermore, the structure of the complex **3** obtained by this method was aslo confirmed by X-ray crystallographic analysis.

Detection of biphenyl derivatives in catalytic reaction with P(*p*-$CF_3C_6H_4)_3$ as the ligand .

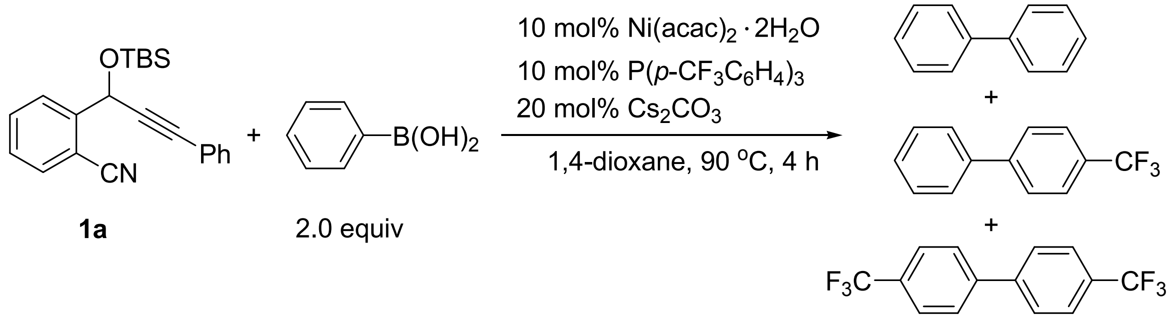

In a nitrogen-filled glovebox, $Ni(acac)_2·2H_2O$ (0.1 mmol, 29.3 mg), P(*p*-$CF_3C_6H_4)_3$ (0.1 mmol, 46.6 mg), Cs_2CO_3 (0.2 mmol, 65.2 mg) and phenylboronic acid (2.0 mmol,

243.9 mg) were added to an oven-dried sealable tube, then 1,4-dioxane (6.0 mL) and *o*-(cyano)phenyl propargyl ether **1a** (347.5 mg, 1.0 mmol) were added. The tube cap was then securely fitted and sealed with electrical tape before it was removed from the glovebox. Then the sealed tube was taken outside the glovebox and stirred at 90 °C in an oil-bath for 4 h. After the mixture was cooled down to room temperature, it was filtered through a short silica gel column and washed with ethyl acetate. The solvent was evaporated under the reduced pressure and the residue was purified by preparative TLC on silica gel (eluent: *n*-pentane) to afford the biphenyl in 16% yield (2.4 mg), and a mixture of 4-(trifluoromethyl)biphenyl and 4,4'-bis(trifluoromethyl)biphenyl (4.9 mg) as a white solid.

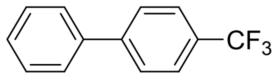

4-(Trifluoromethyl)biphenyl. ^{1}H NMR(400 MHz, $CDCl_3$, Me_4Si): δ 7.40-7.44 (m, 1H), 7.47-7.51 (m, 2H), 7.59-7.62 (m, 2H), 7.70 (br, 4H). HRMS (EI) calcd for $C_{13}H_9F_3$ $[M]^+$: 222.0656, found 222.0652. The ^{1}H NMR data is in agreement with that previously reported.[3]

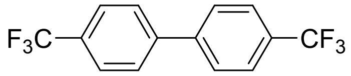

4,4'-Bis(trifluoromethyl)biphenyl. ^{1}H NMR(400 MHz, $CDCl_3$, Me_4Si): δ 7.71 (d, *J* = 8.4 Hz, 4H), 7.75 (d, *J* = 8.8 Hz, 4H). HRMS (EI) calcd for $C_{14}H_8F_6$ $[M]^+$: 290.0530, found 290.0522. The ^{1}H NMR data is in agreement with that previously reported.[4]

Without the ligand of P(*p*-$CF_3C_6H_4$)$_3$, only biphenyl was observed in 48% yield (7.4 mg) as a white solid.

Transformation of 1a to 2a using IPrNi(acac) 3 as the catalyst.

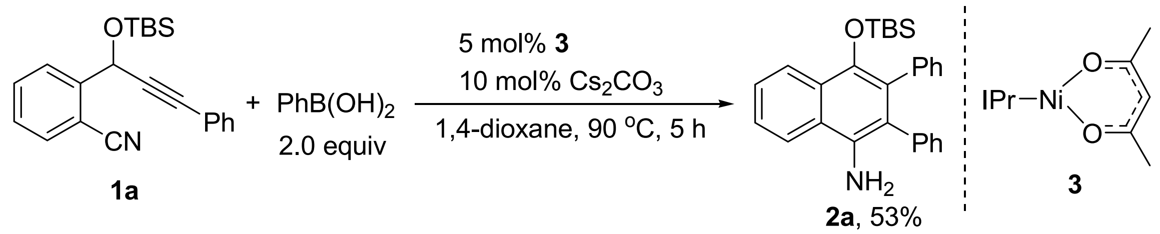

In a nitrogen-filled glovebox, IPrNi(acac) **3** (0.015 mmol, 8.2 mg), Cs_2CO_3 (0.03 mmol, 9.8 mg) and phenylboronic acid (0.6 mmol, 73.2 mg) were added to an oven-dried screw-cap vial (volume: 4.0 mL), then 1,4-dioxane (2.0 mL) and *o*-(cyano)phenyl propargyl ether **1a** (104.3 mg, 0.3 mmol) were added. The vial cap was then securely fitted and sealed with electrical tape before the vial was removed from the glovebox. Then the vial was taken outside the glovebox and stirred at 90 ^{o}C in an oil-bath for 5 h. After the mixture was cooled down to room temperature, it was filtered through a short silica gel column and washed with ethyl acetate. The solvent was evaporated under the reduced pressure and the residue was purified by column chromatography on silica gel which was treated with petroleum ether/Et_3N = 1:1 and then petroleum ether before loading the sample (eluent: petroleum ether: ethyl acetate = 15:1) to afford **2a** in 53% yield (67.4 mg) as a light yellow solid.

Exclude the possibility of allene 4 as the intermediate.

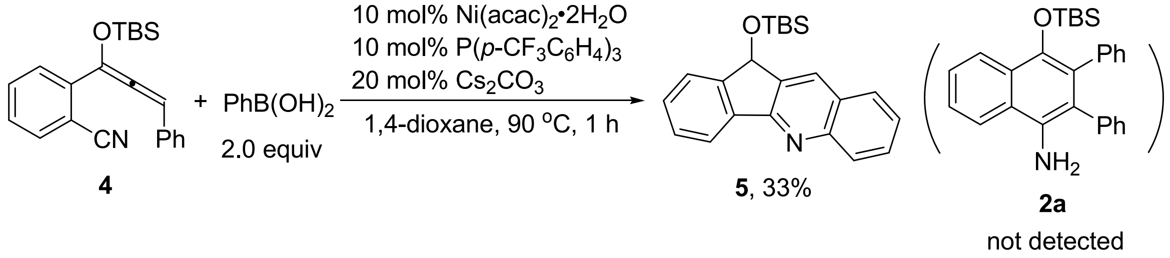

In a nitrogen-filled glovebox, $Ni(acac)_2 \cdot 2H_2O$ (0.03 mmol, 8.8 mg), P(*p*-$CF_3C_6H_4)_3$ (0.03 mmol, 14.0 mg), Cs_2CO_3 (0.06 mmol, 19.5 mg) and phenylboronic acid (0.6 mmol, 73.2 mg) were added to an oven-dried screw-cap vial (volume: 4.0 mL), then 1,4-dioxane (2.0 mL) and allene **4**[1] (104.3 mg, 0.3 mmol) were added. The vial cap was then securely fitted and sealed with electrical tape before the vial was removed from the glovebox. Then the vial was taken outside the glovebox and stirred at 90 ^{o}C in an oil-bath for 1 h. After the mixture was cooled down to room temperature, it was filtered through a short silica gel

column and washed with ethyl acetate. The solvent was evaporated under the reduced pressure and the residue was purified by column chromatography on silica gel (eluent: petroleum ether: ethyl acetate = 25:1) to afford indeno[1,2-*b*]quinolone **5** in 33% yield (34.5 mg) as a light yellow solid, while the desired **2a** was not observed.

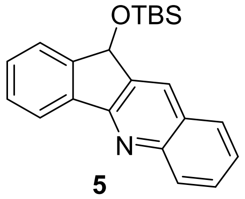

5

11-((*tert*-Butyldimethylsilyl)oxy)-11*H*-indeno[1,2-*b*]quinoline (5). ^{1}H NMR(400 MHz, $CDCl_3$, Me_4Si): δ 0.18 (s, 3H), 0.20 (s, 3H), 0.98 (s, 9H), 5.88 (s, 1H), 7.48-7.55 (m, 3H), 7.63-7.65 (m, 1H), 7.71 (td, *J* = 6.8, 1.6 Hz, 1H), 7.84 (dd, *J* = 8.2, 0.8 Hz, 1H), 8.15-8.19 (m, 3H). ^{13}C NMR(100 MHz, $CDCl_3$, Me_4Si): δ -3.71, -3.65, 18.12, 25.81, 73.09, 121.79, 125.34, 126.00, 127.56, 128.27, 129.14, 129.33, 129.45, 130.59, 131.68, 137.93, 139.04, 148.07, 148.83, 160.07. The NMR data is in agreement with that previously reported.[1]

Synthesis of 2-(3-phenylprop-2-ynyl)benzonitrile (6).

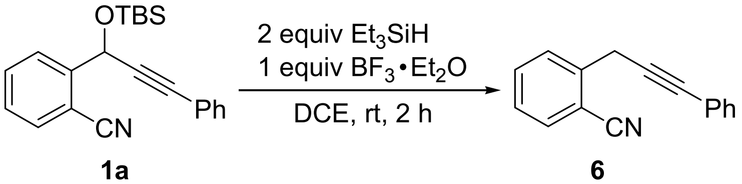

To a Schlenk tube were added **1a** (5.0 mmol, 1.74 g), dichloroethane (20.0 mL) and Et_3SiH (10.0 mmol, 1.6 mL). Then $BF_3 \cdot Et_2O$ was added dropwise at room temperature, and the reaction mixture was stirred at the same temperature for 2 h. The resulting mixture was quenched with water, and extracted with dichloromethane. The combined organic extracts were washed with water and brine, and dried over Na_2SO_4. The solvent was evaporated under the reduced pressure, and the residue was purified by column chromatography on silica gel (eluent: petroleum ether: ethyl acetate = 30:1) to afford **6** in 46% yield (494.5 mg) as a yellow oil. ^{1}H NMR(400 MHz, $CDCl_3$, Me_4Si): δ 4.06 (s, 2H), 7.30-7.34 (m, 3H), 7.37 (t, *J* = 7.6 Hz, 1H), 7.46-7.49 (m, 2H), 7.61 (dt, *J* = 1.6, 7.6 Hz,

1H), 7.66 (dd, J = 0.8, 7.6 Hz, 1H), 7.77 (d, J = 8.0 Hz, 1H). ^{13}C NMR(100 MHz, $CDCl_3$, Me_4Si): δ 24.62, 83.96, 84.82, 111.87, 117.36, 122.99, 127.30, 128.16, 128.25, 129.02, 131.64, 132.70, 133.10, 140.45. IR (film): 3062, 3031, 2224, 1599, 1489, 1450, 1442, 1412, 1330, 1207, 1093, 1070, 1029, 915, 754, 690 cm^{-1}. HRMS (EI) calcd for $C_{16}H_{11}N$ $[M]^+$: 217.0891, found 217.0884.

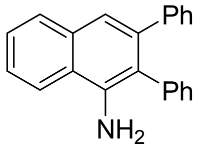

7

2,3-Diphenylnaphthalen-1-amine (7). Compound **7** was synthesized according to the typical procedure described for **2c**. (0.3 mmol scale, $Ni(acac)_2{\cdot}2H_2O$ (0.03 mmol, 8.8 mg), $P(p\text{-}CF_3C_6H_4)_3$ (0.03 mmol, 14.0 mg), Cs_2CO_3 (0.06 mmol, 19.5 mg), phenylboronic acid (0.6 mmol, 73.2 mg), 1,4-dioxane (2.0 mL) and 2-(3-phenylprop-2-ynyl)benzonitrile (**6**) (65.2 mg, 0.3 mmol) were stirred at 90 °C for 5 h. Purification of the crude product by column chromatography on silica gel (eluent: petroleum ether: ethyl acetate = 20:1) followed by twice preparative TLC on silica gel (which was treated with Et_3N before loading the sample) (eluent: petroleum ether: ethyl acetate = 20:1) afforded the title product in 10% yield (9.3 mg) as a yellow oil. A small amount of an unidentified byproduct (15.2 mg) was also obtained. ^{1}H NMR(400 MHz, C_6D_6, Me_4Si): δ 3.75 (s, 2H), 6.94-6.99 (m, 2H), 7.02-7.07 (m, 4H), 7.16-7.17 (m, 2H), 7.21-7.24 (m, 2H), 7.25-7.28 (m, 1H), 7.32 (dt, J = 0.8, 6.8 Hz, 1H), 7.41 (s, 1H), 7.48 (d, J = 8.4 Hz, 1H), 7.71 (d, J = 7.6 Hz, 1H). ^{13}C NMR(100 MHz, C_6D_6, Me_4Si): δ 119.92, 121.58, 121.68, 123.38, 125.13, 126.38, 126.44, 127.03, 127.86, 128.81, 128.95, 130.28, 131.76, 134.16, 139.07, 140.10, 140.87, 142.92. IR (film): 3467, 3383, 3054, 2924, 1710, 1613, 1564, 1493, 1436, 1398, 1371, 1104, 1072, 1027, 912, 868, 839, 789, 766, 743, 700 cm^{-1}. HRMS (ESI) calcd for $C_{22}H_{18}N$ $[M+H]^+$: 296.1434, found 296.1436.

Reaction of 1a in the presence of TEMPO.

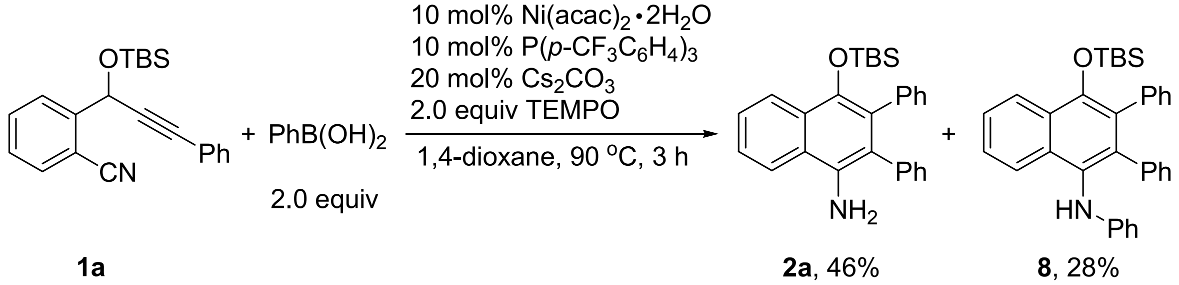

In a nitrogen-filled glovebox, $Ni(acac)_2 \cdot 2H_2O$ (0.03 mmol, 8.8 mg), $P(p\text{-}CF_3C_6H_4)_3$ (0.03 mmol, 14.0 mg), Cs_2CO_3 (0.06 mmol, 19.5 mg), phenylboronic acid (0.6 mmol, 73.2 mg) and TEMPO (0.6 mmol, 93.8 mg) were added to an oven-dried screw-cap vial (volume: 4.0 mL), then 1,4-dioxane (2.0 mL) and *o*-(cyano)phenyl propargyl ether **1a** (104.3 mg, 0.3 mmol) were added. The vial cap was then securely fitted and sealed with electrical tape before the vial was removed from the glovebox. Then the vial was taken outside the glovebox and stirred at 90 °C in an oil-bath for 3 h. After the mixture was cooled down to room temperature, it was filtered through a short silica gel column and washed with ethyl acetate. The solvent was evaporated under the reduced pressure and the residue was purified by column chromatography on silica gel (eluent: petroleum ether: ethyl acetate = 100:1 to 15:1 grdient) to afford **8** in 28% yield (41.7 mg) as a brown viscous oil and **2a** in 46% yield (58.5 mg) as a light yellow solid.

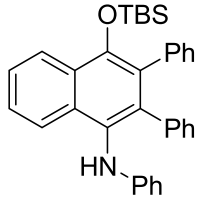

8

4-(*tert*-Butyldimethylsilyloxy)-*N*,2,3-triphenylnaphthalen-1-amine (8). ^{1}H NMR(400 MHz, C_6D_6, Me_4Si): δ -0.24 (s, 6H), 1.03 (s, 9H), 5.14 (s, 1H), 6.44 (d, *J* = 7.6 Hz, 2H), 6.70 (t, *J* = 7.6 Hz, 1H), 6.85-7.02 (m, 10H), 7.21-7.28 (m, 3H), 7.39-7.43 (m, 1H), 8.23 (d, *J* = 8.0 Hz, 1H), 8.53 (d, *J* = 8.4 Hz, 1H). ^{13}C NMR(100 MHz, C_6D_6, Me_4Si): δ -3.70, 18.91, 26.38, 114.95, 119.04, 124.27, 125.89, 126.07, 126.57, 126.94, 126.96, 127.61, 128.06, 129.18, 129.24, 129.46, 130.54, 130.69, 131.92, 132.87, 136.90, 138.40, 138.73, 146.92, 148.82. IR (film): 3393, 3053, 2955, 2928, 2857, 2360, 2342, 1716, 1601, 1568,

1496, 1472, 1447, 1436, 1370, 1304, 1259, 1219, 1168, 1085, 1072, 1030, 927, 907, 827, 813, 781, 765, 748, 697, 678, 669, 661 cm^{-1}. HRMS (ESI) calcd for $C_{34}H_{36}NOSi$ $[M+H]^+$: 502.2561, found 502.2561.

Reaction of 2a in the presence of TEMPO.

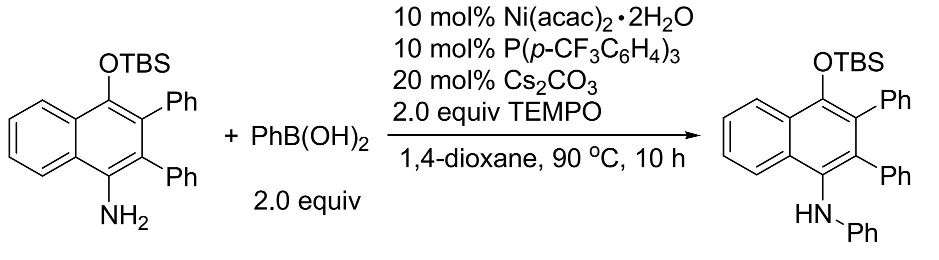

2a **8**, 43%

In a nitrogen-filled glovebox, $Ni(acac)_2 \cdot 2H_2O$ (0.03 mmol, 8.8 mg), $P(p\text{-}CF_3C_6H_4)_3$ (0.03 mmol, 14.0 mg), Cs_2CO_3 (0.06 mmol, 19.5 mg), phenylboronic acid (0.6 mmol, 73.2 mg), TEMPO (0.6 mmol, 93.8 mg) and 4-((*tert*-Butyldimethylsilyl)oxy)-2,3-diphenylnaphthalen-1-amine **2a** (0.3 mmol, 127.7 mg) were added to an oven-dried screw-cap vial (volume: 4.0 mL), then 1,4-dioxane (2.0 mL) were added. The vial cap was then securely fitted and sealed with electrical tape before the vial was removed from the glovebox. Then the vial was taken outside the glovebox and stirred at 90 °C in an oil-bath for 10 h. After the mixture was cooled down to room temperature, it was filtered through a short silica gel column and washed with ethyl acetate. The solvent was evaporated under the reduced pressure and the residue was purified by column chromatography on silica gel (eluent: petroleum ether: ethyl acetate = 100:1) to afford **8** in 43% yield (64.7 mg) as a brown viscous oil.

References:

[1] X. You, X. Xie, H. Chen, Y. Li, Y. Liu, *Chem. Eur. J.* **2015**, *21*, 18699.

[2] Y. N. Cheng, X. Y. Gu, P. X. Li, *Org. Lett.* **2013**, *15*, 2664.

[3] Y. Li, L. Wu, H. Neumann, M. Beller, *Chem. Commun.* **2013**, *49*, 2628.

[4] Q. Zhao, L. Chen, H. Lang, S. Wu, L. Wang, *Chin. J. Chem.* **2015**, *33*, 535.

X-ray crystal structures

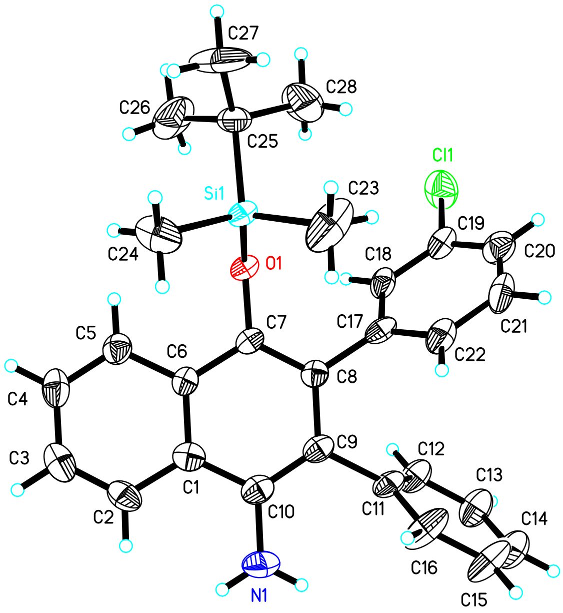

Figure 1. X-ray crystal structure of compound **2o**

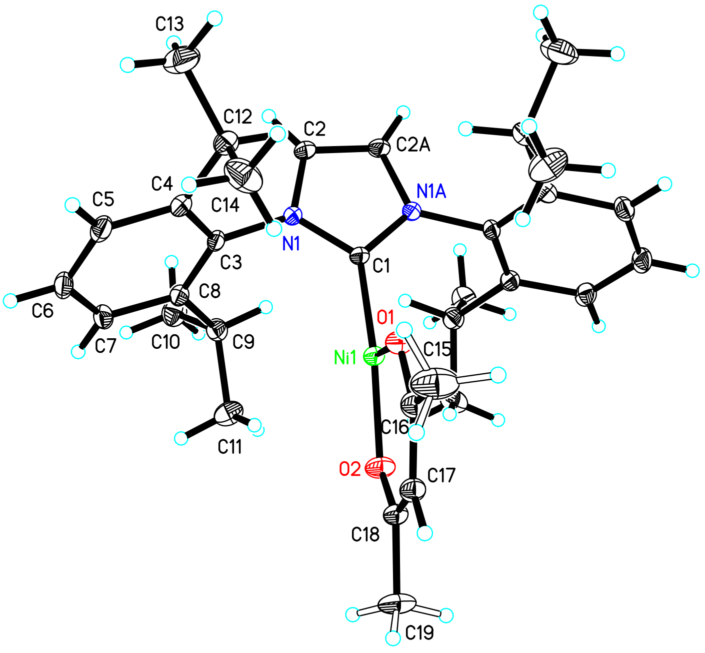

Figure 2. X-ray crystal structure of compound **3**

^{1}H NMR(400 MHz, $CDCl_3$, Me_4Si)

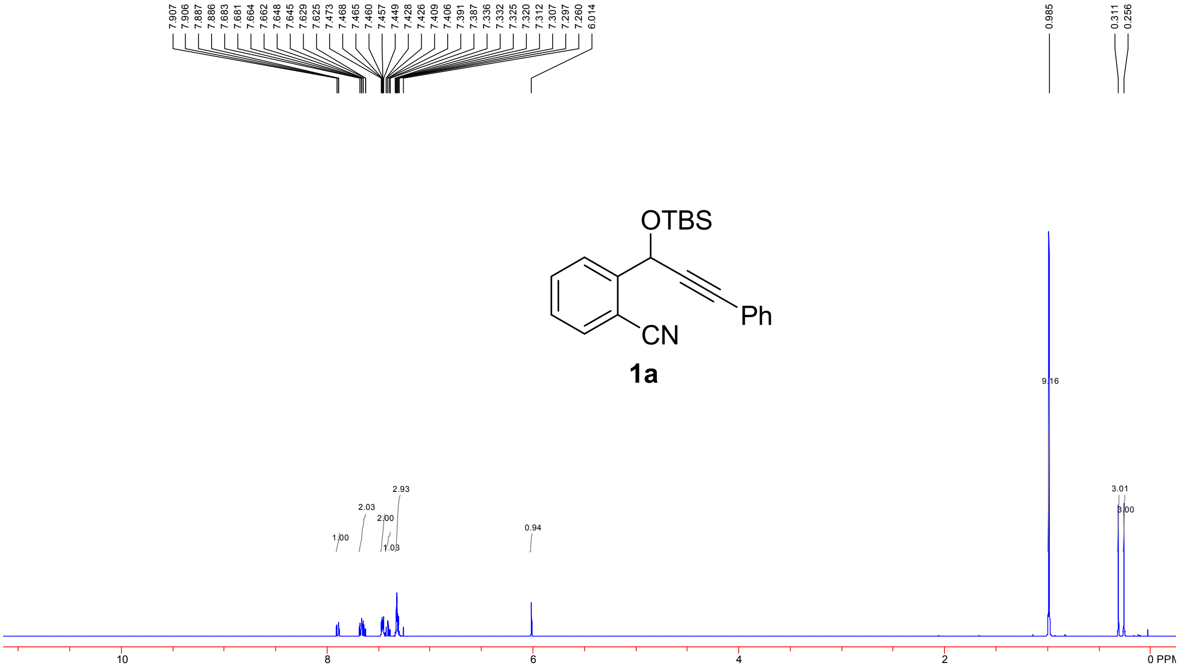

^{13}C NMR(100 MHz, $CDCl_3$, Me_4Si)

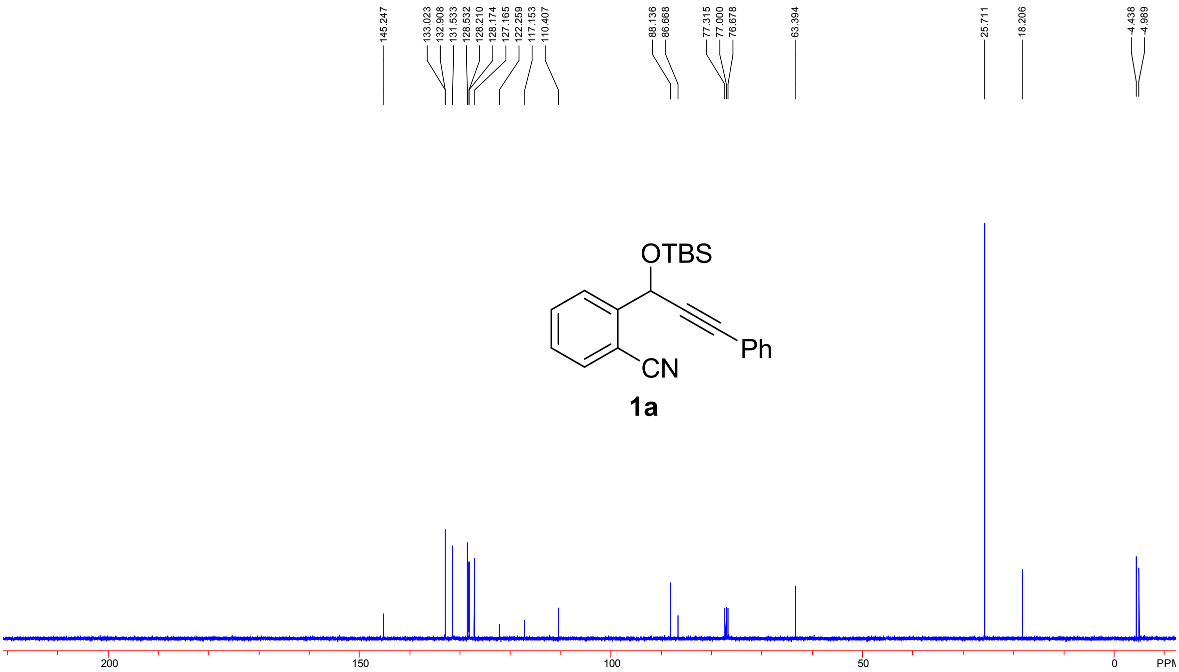

^{1}H NMR(400 MHz, $CDCl_3$, Me_4Si)

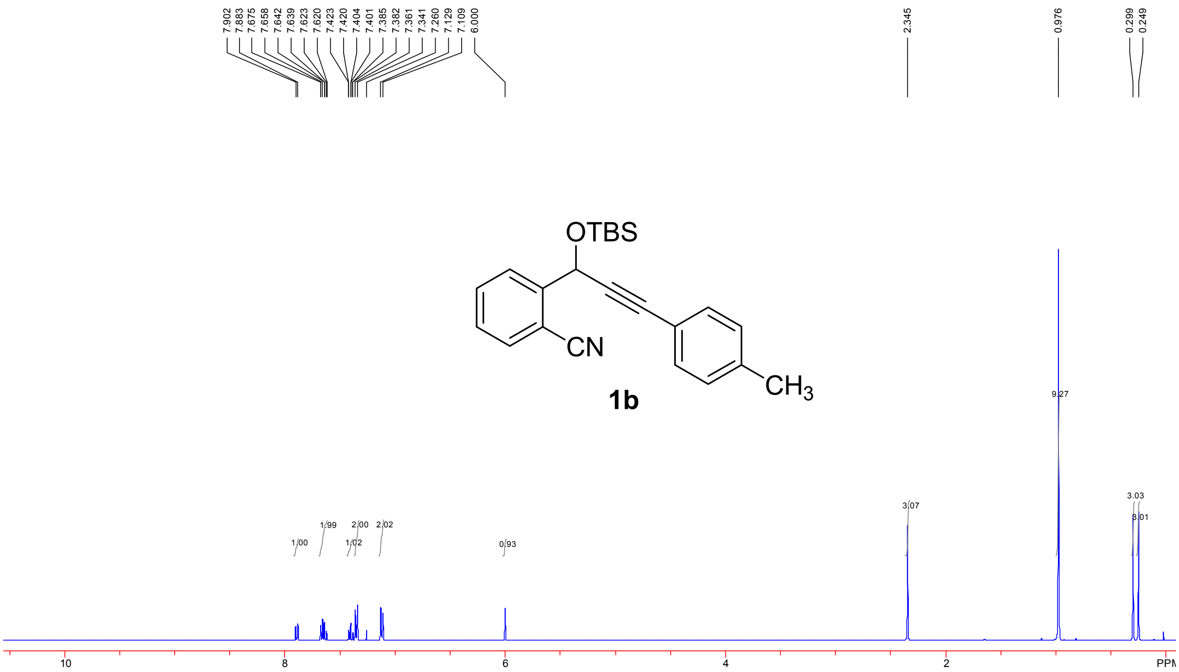

^{13}C NMR(100 MHz, $CDCl_3$, Me_4Si)

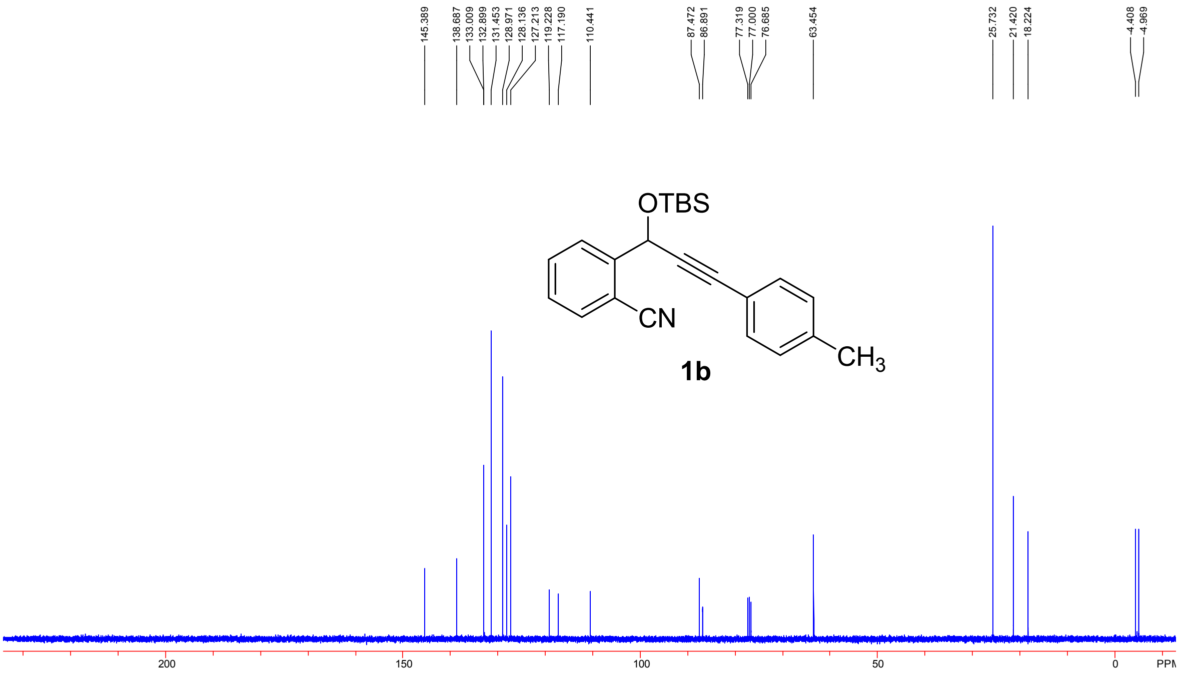

^{1}H NMR(400 MHz, $CDCl_3$, Me_4Si)

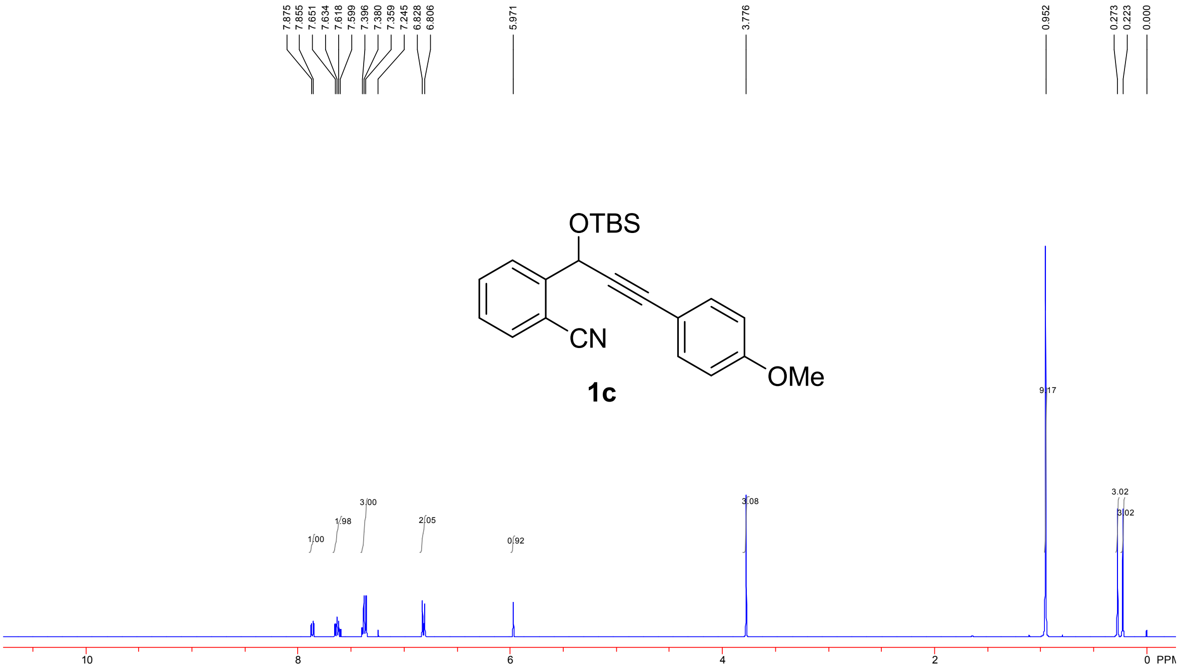

^{13}C NMR(100 MHz, $CDCl_3$, Me_4Si)

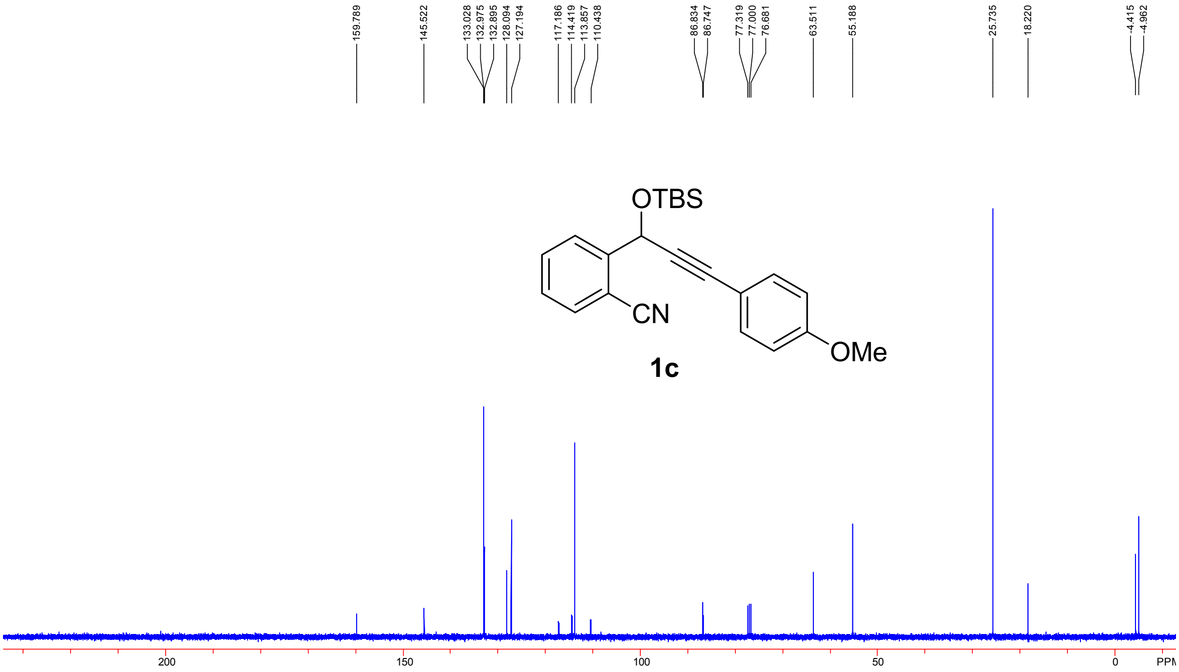

^{1}H NMR(400 MHz, $CDCl_3$, Me_4Si)

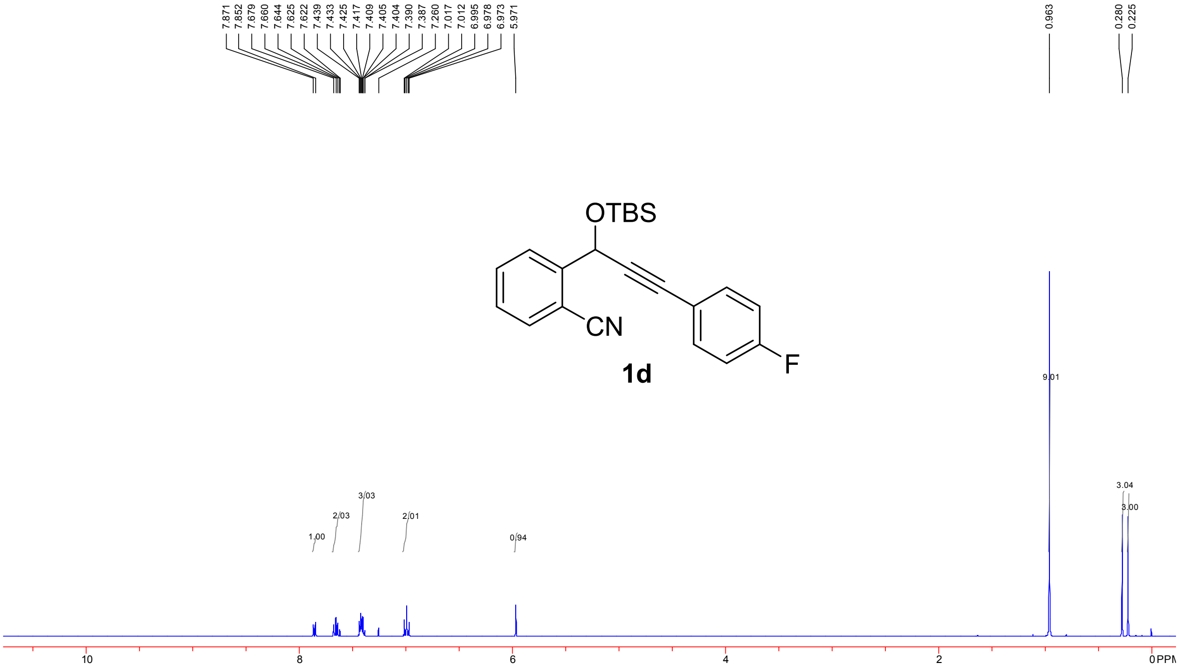

^{13}C NMR(100 MHz, $CDCl_3$, Me_4Si)

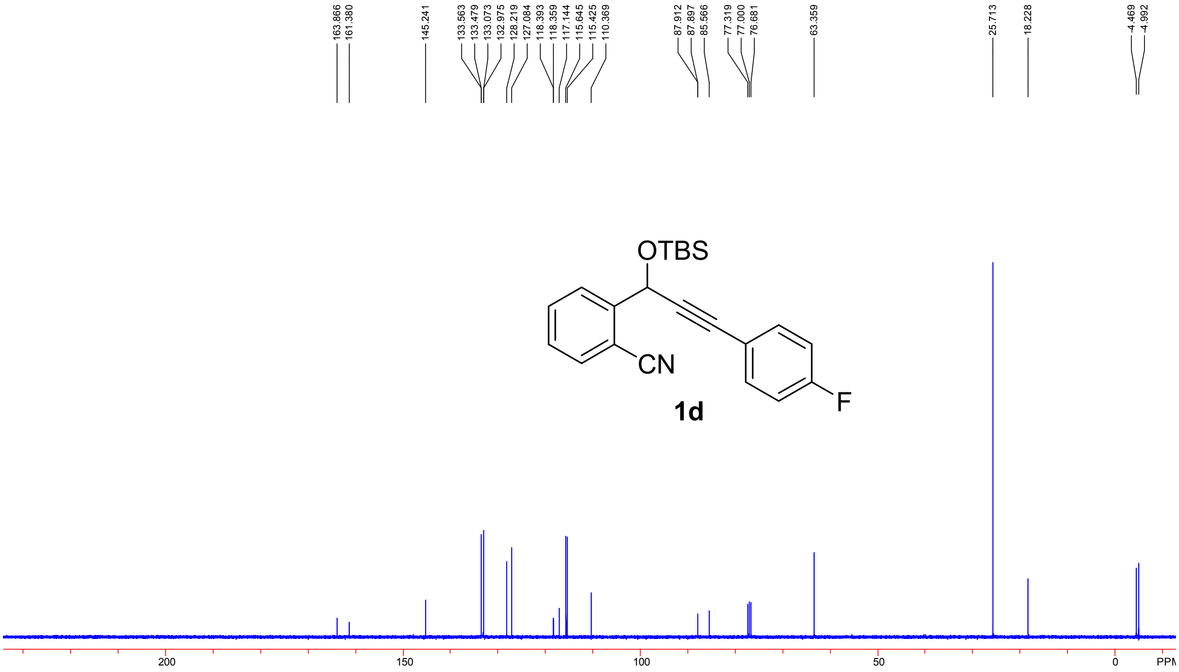

^{1}H NMR(400 MHz, $CDCl_3$, Me_4Si)

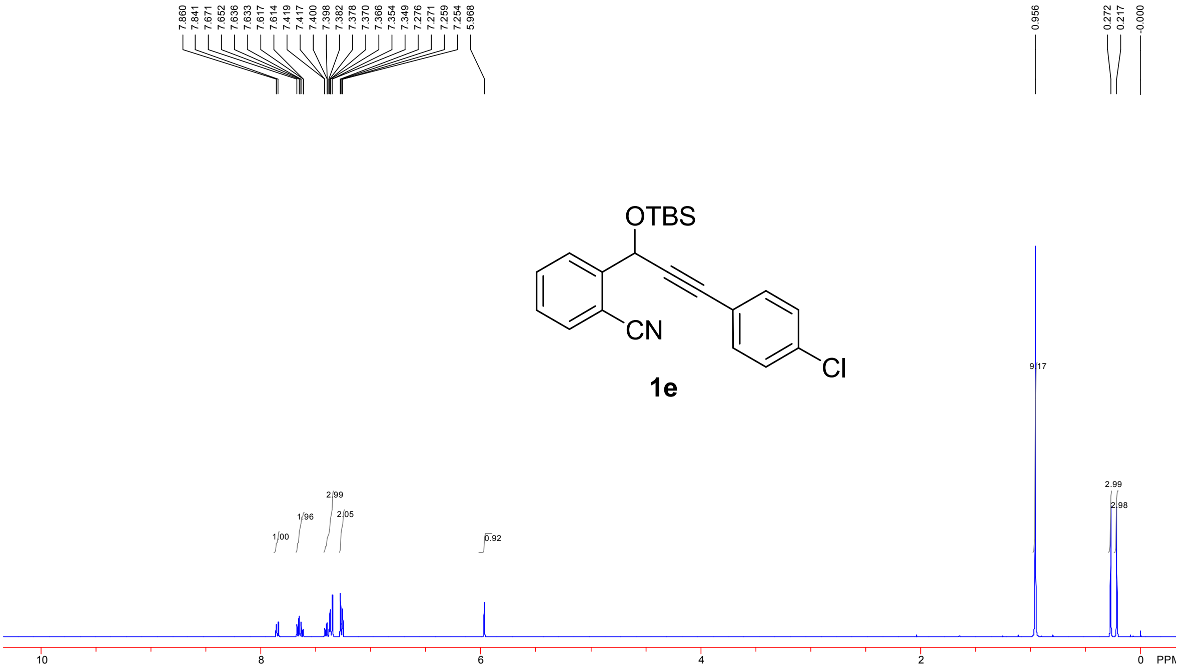

^{13}C NMR(100 MHz, $CDCl_3$, Me_4Si)

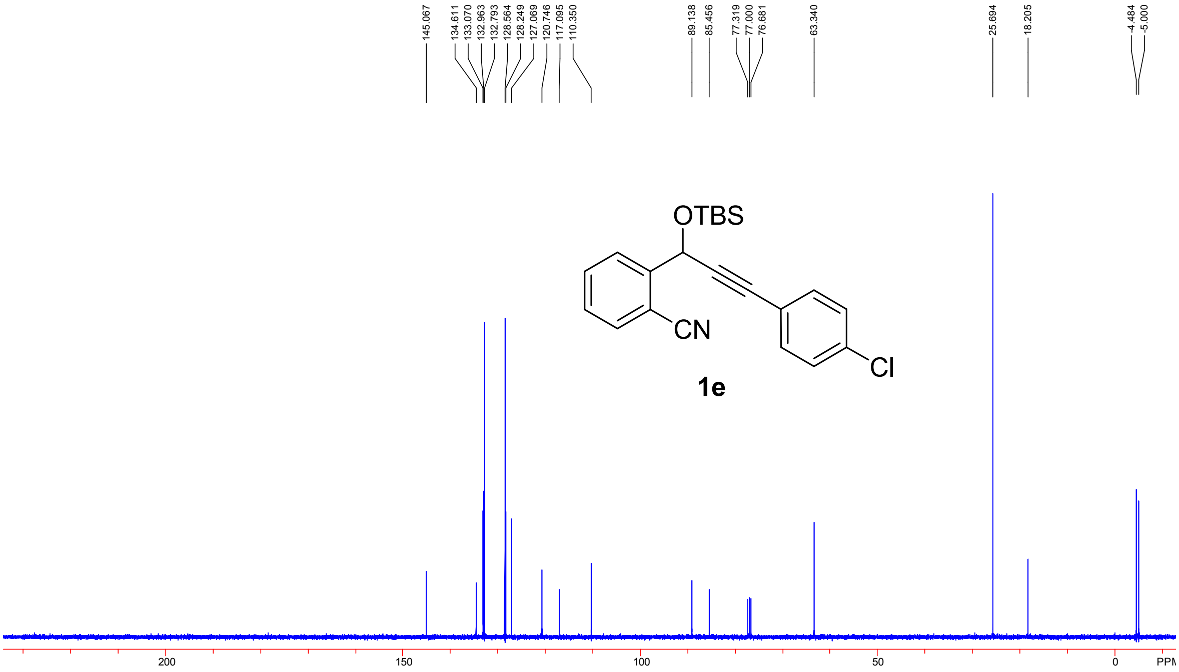

^{1}H NMR(400 MHz, $CDCl_3$, Me_4Si)

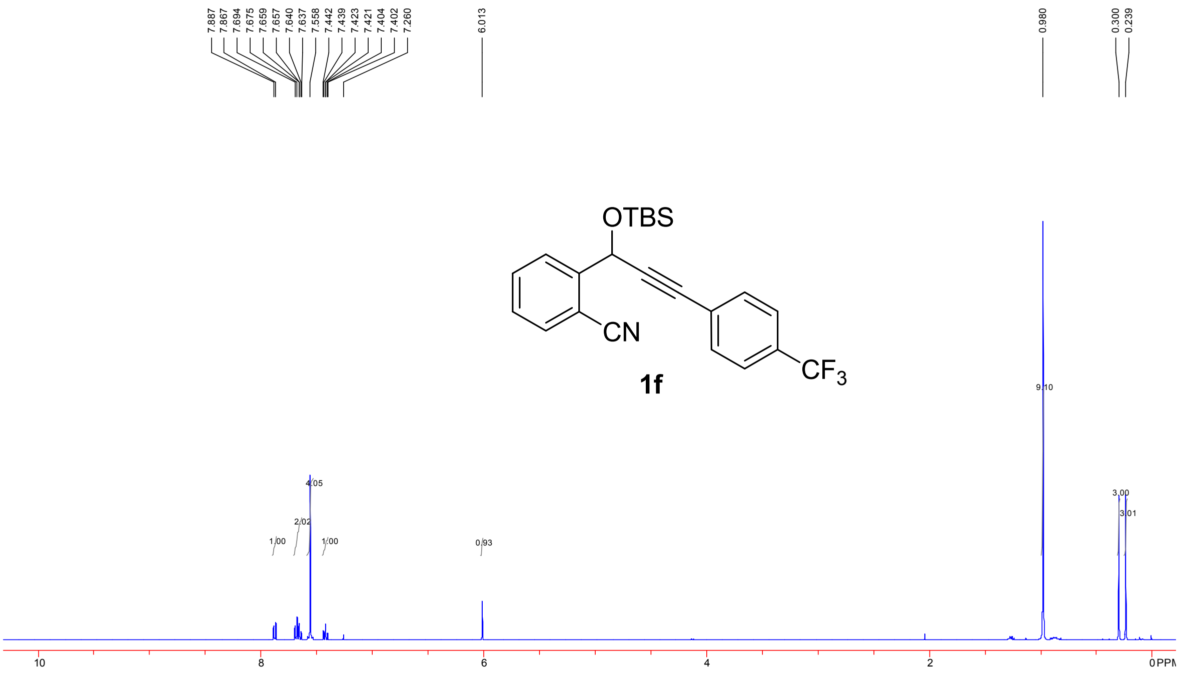

^{13}C NMR(100 MHz, $CDCl_3$, Me_4Si)

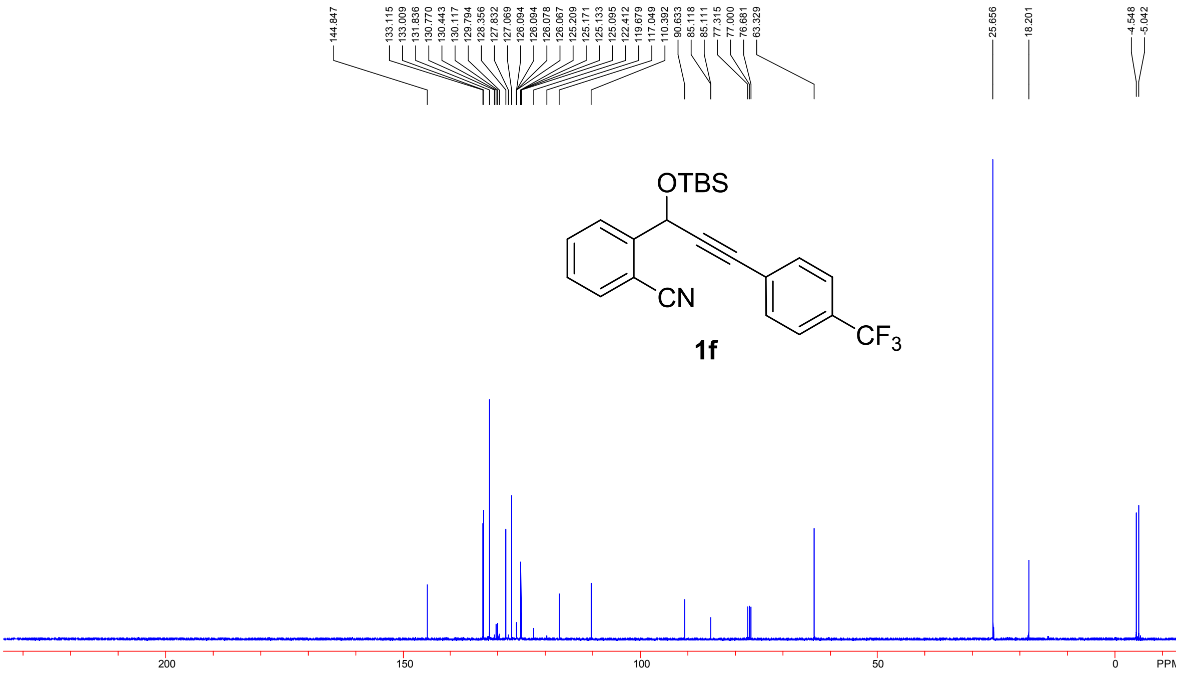

^{1}H NMR(400 MHz, $CDCl_3$, Me_4Si)

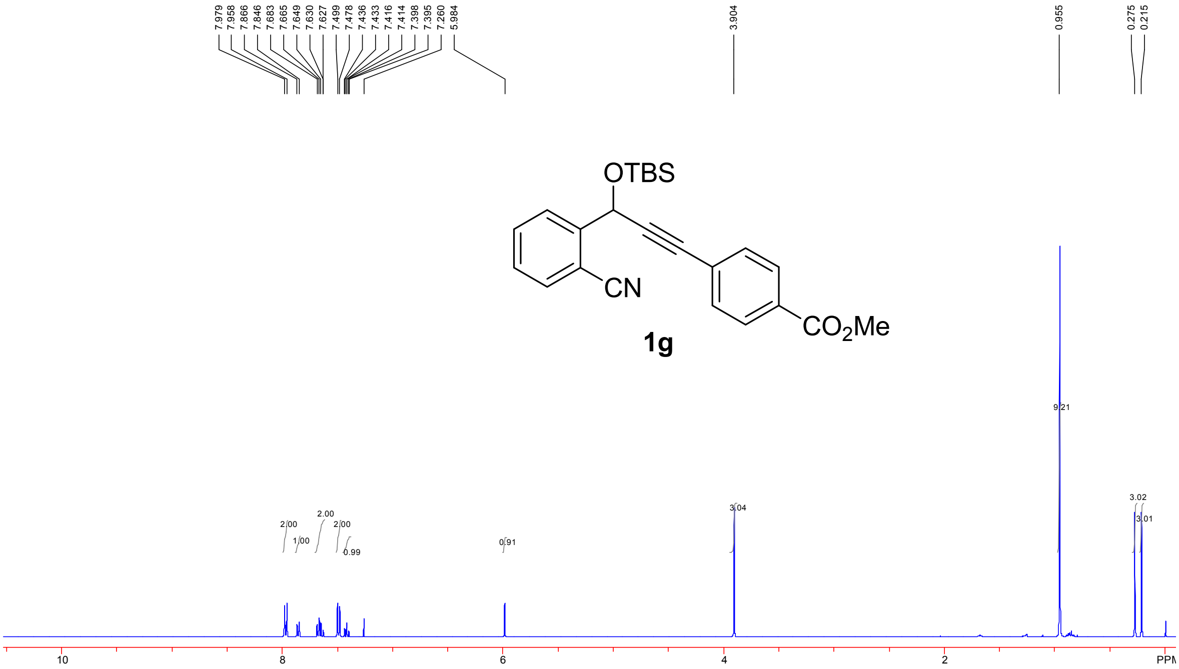

^{13}C NMR(100 MHz, $CDCl_3$, Me_4Si)

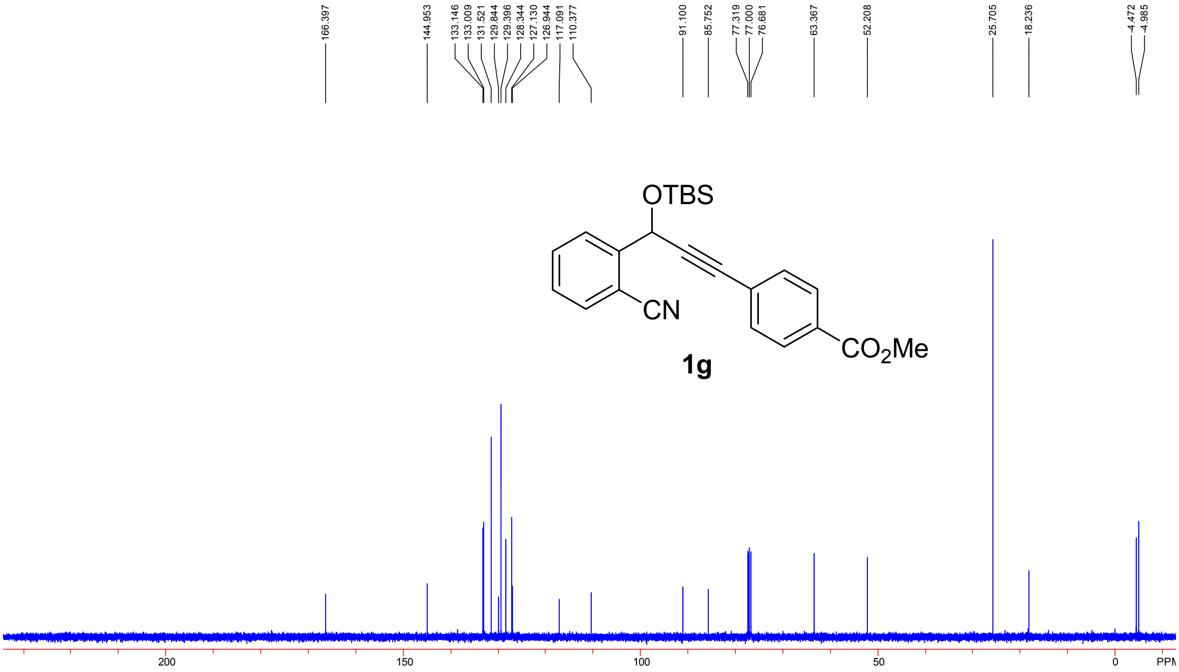

^{1}H NMR(400 MHz, $CDCl_3$, Me_4Si)

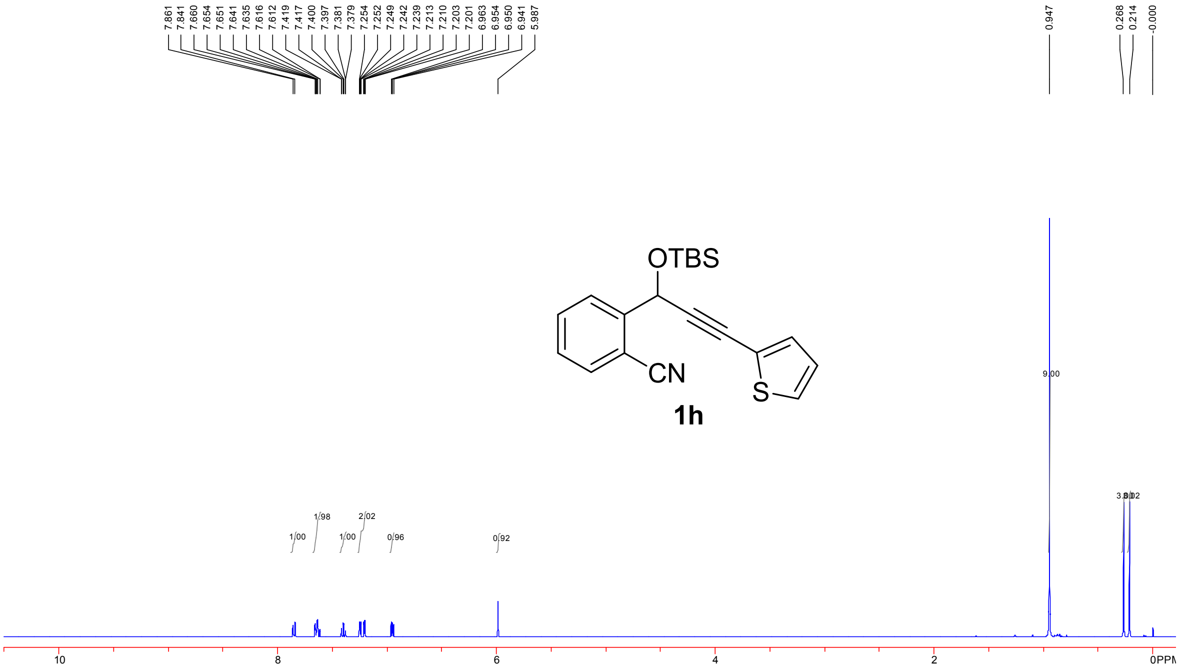

^{13}C NMR(100 MHz, $CDCl_3$, Me_4Si)

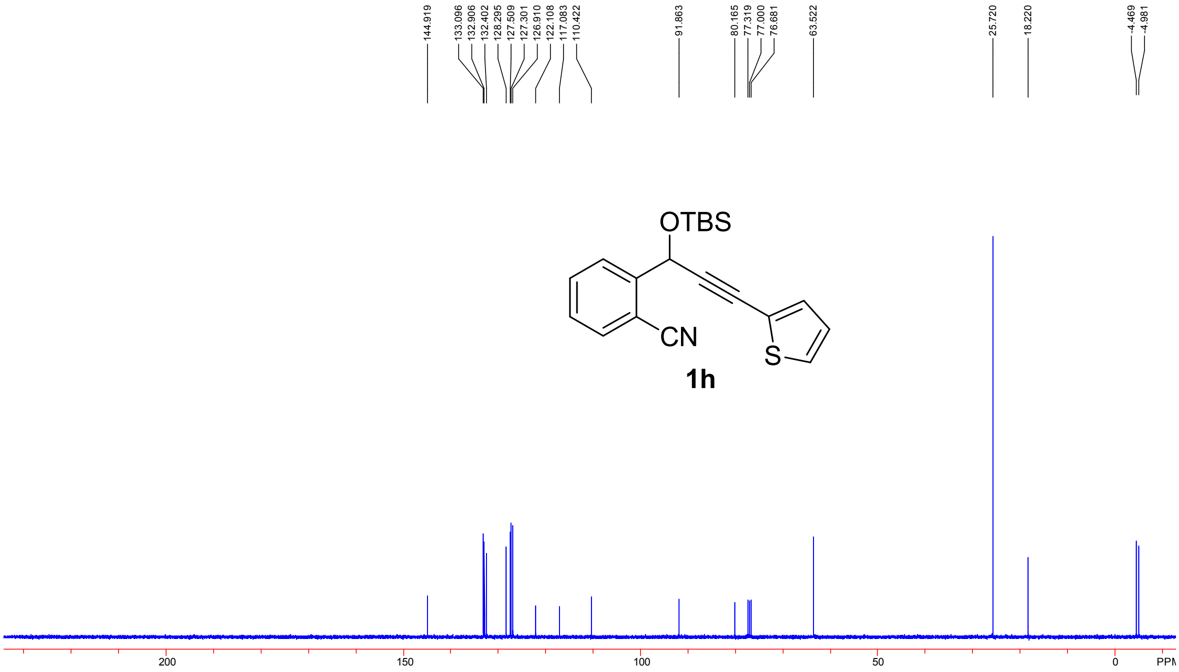

^{1}H NMR(400 MHz, $CDCl_3$, Me_4Si)

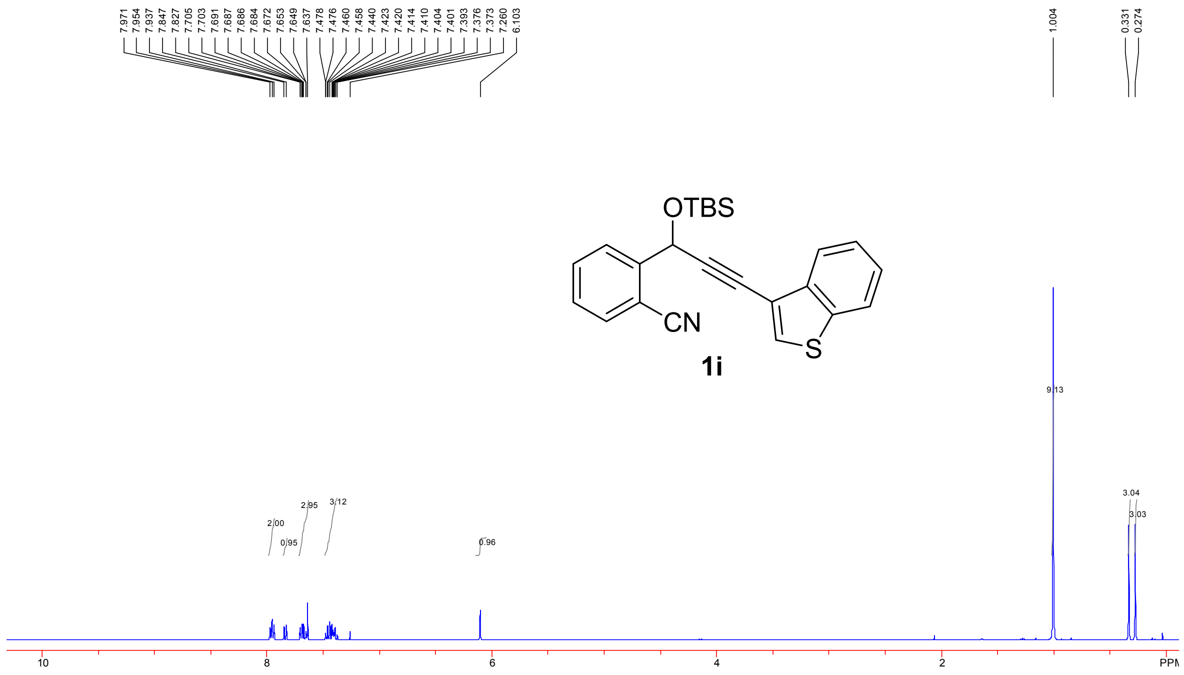

^{13}C NMR(100 MHz, $CDCl_3$, Me_4Si)

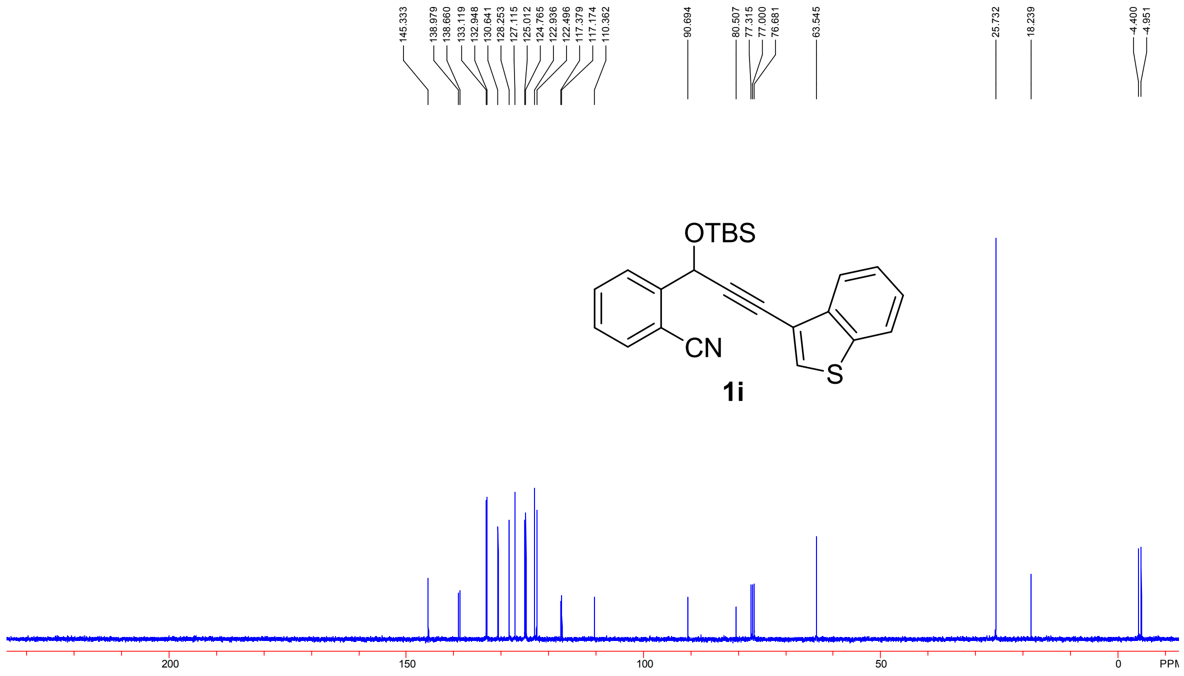

^{1}H NMR(400 MHz, $CDCl_3$, Me_4Si)

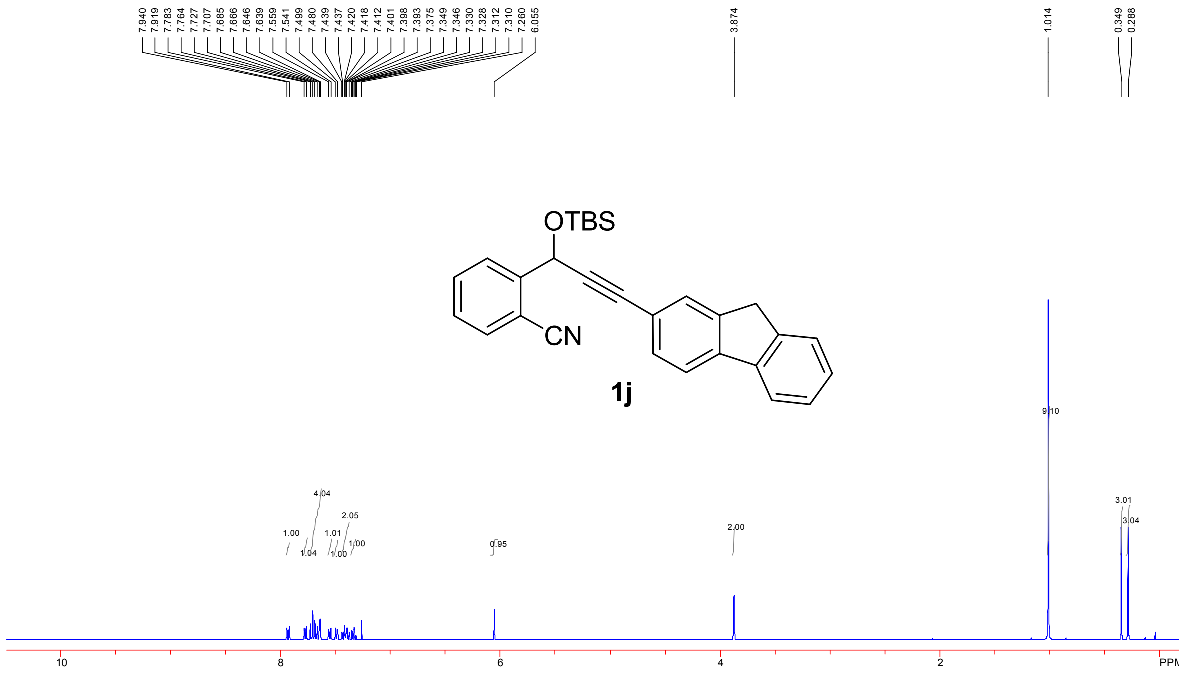

^{13}C NMR(100 MHz, $CDCl_3$, Me_4Si)

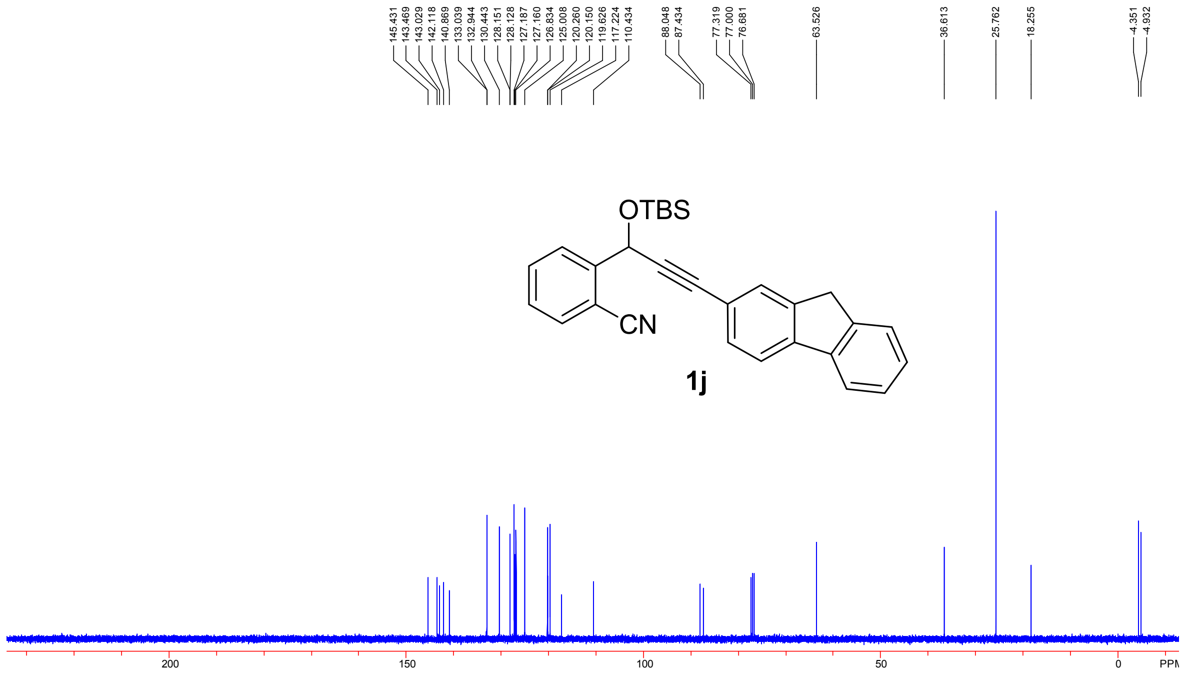

^{1}H NMR(400 MHz, $CDCl_3$, Me_4Si)

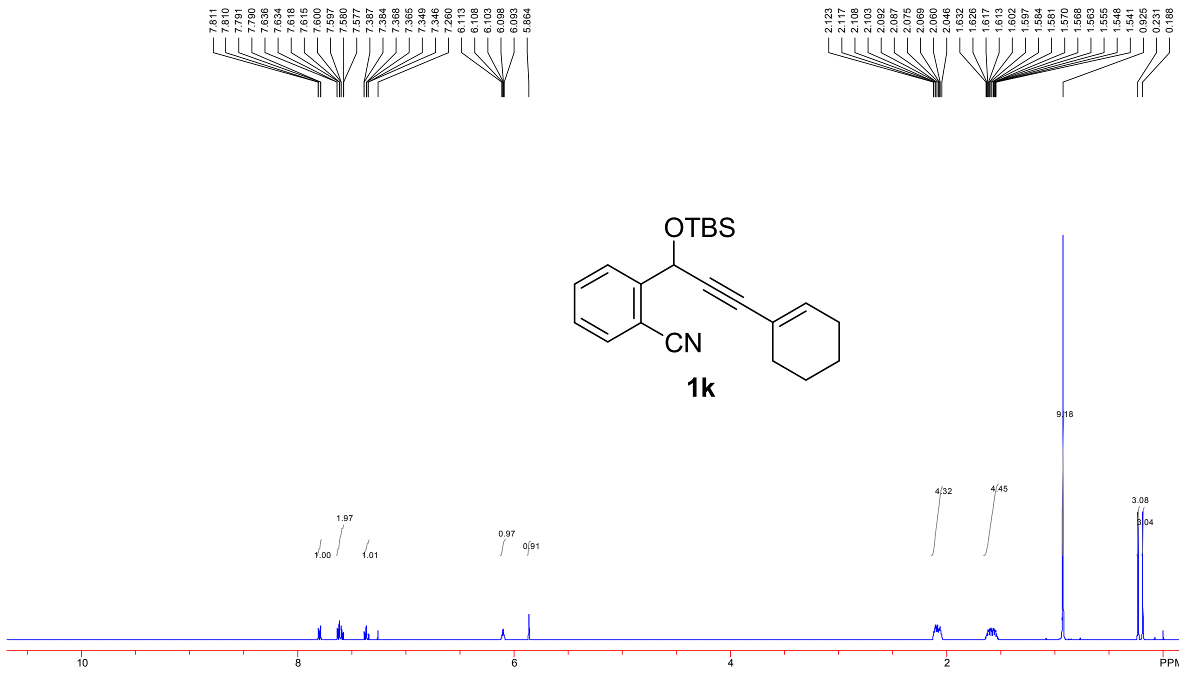

^{13}C NMR(100 MHz, $CDCl_3$, Me_4Si)

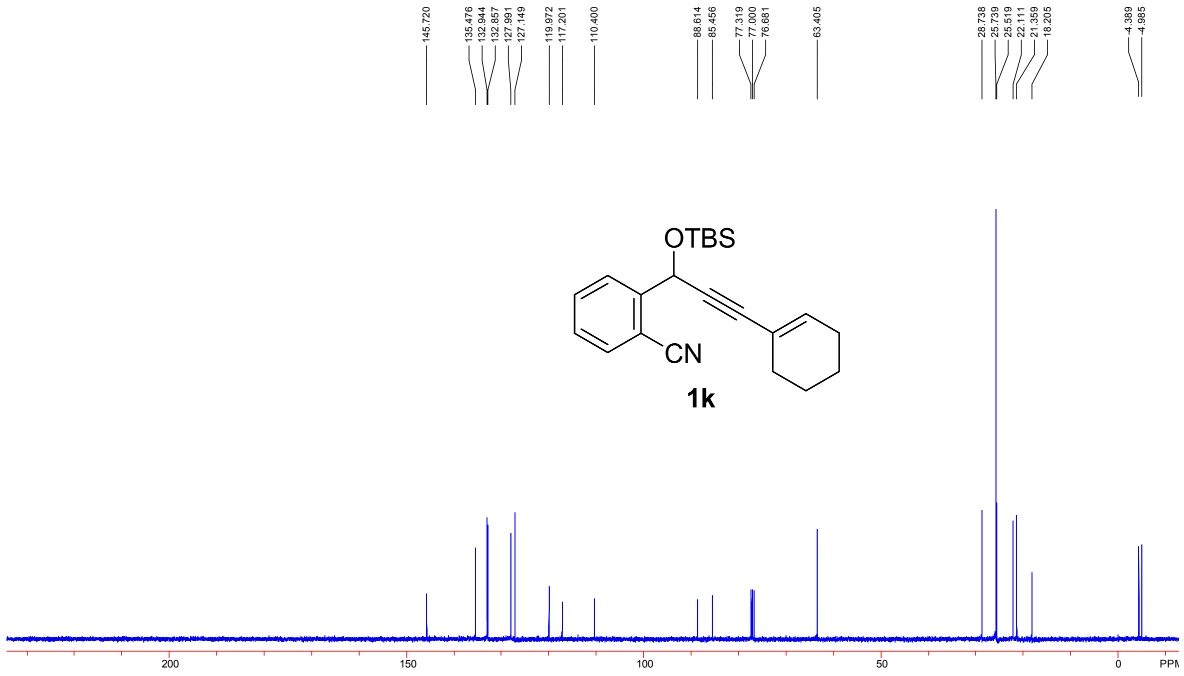

^{1}H NMR(400 MHz, $CDCl_3$, Me_4Si)

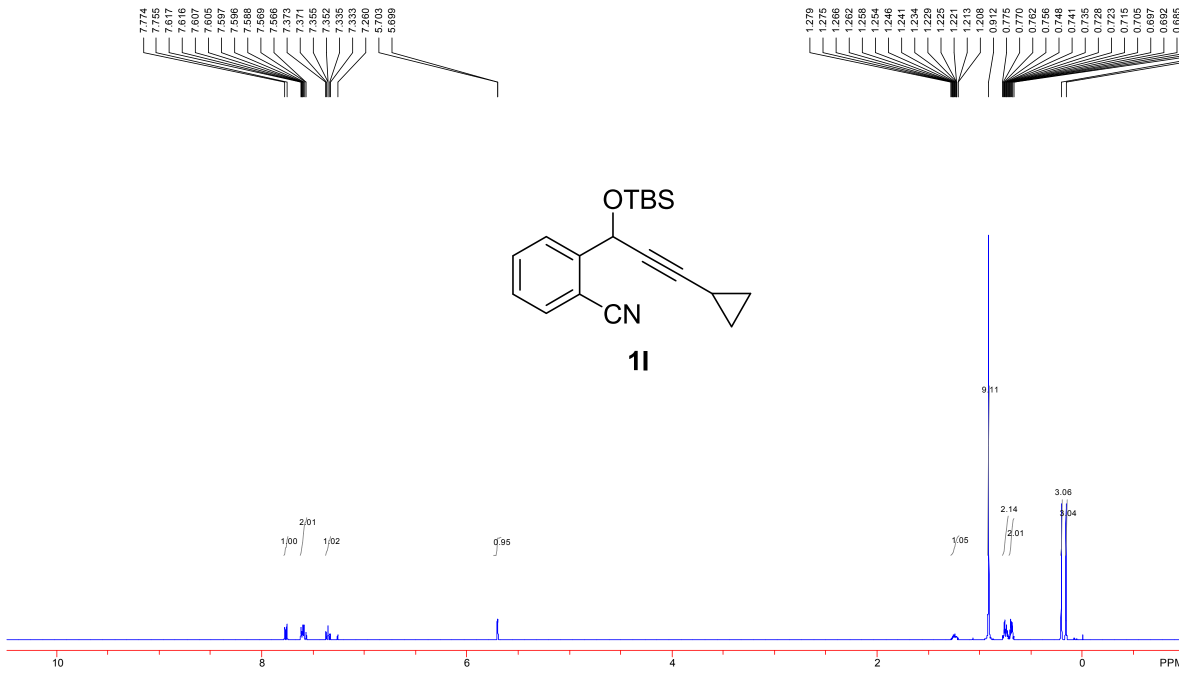

^{13}C NMR(100 MHz, $CDCl_3$, Me_4Si)

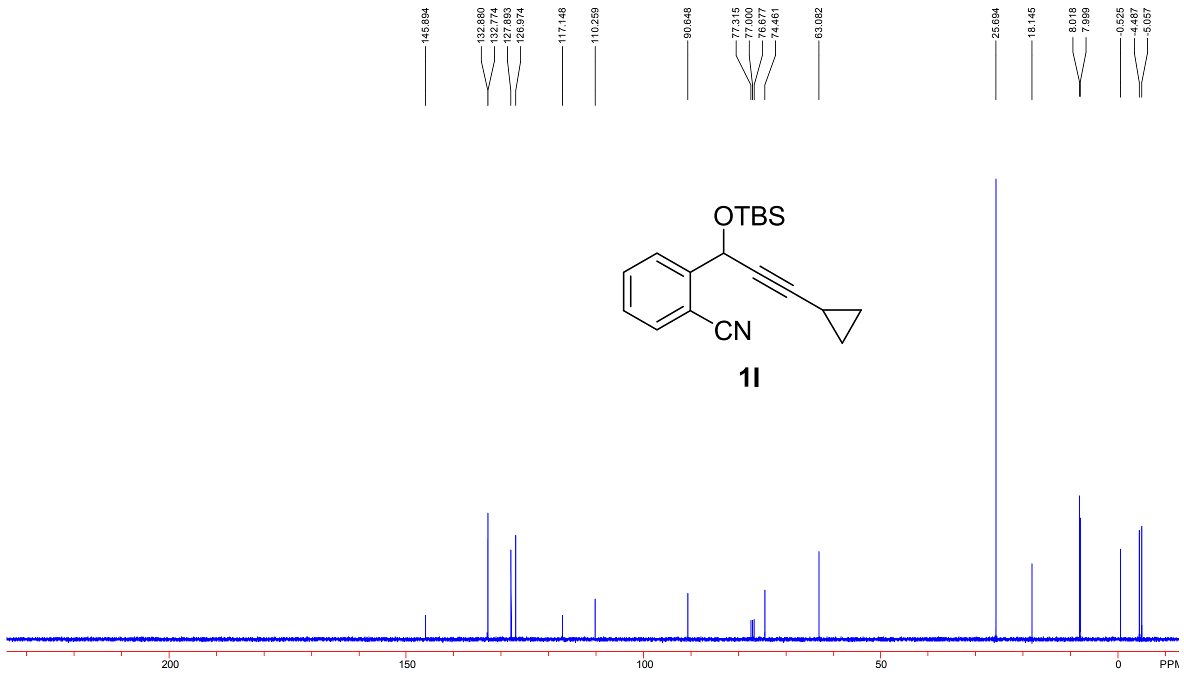

^{1}H NMR(400 MHz, $CDCl_3$, Me_4Si)

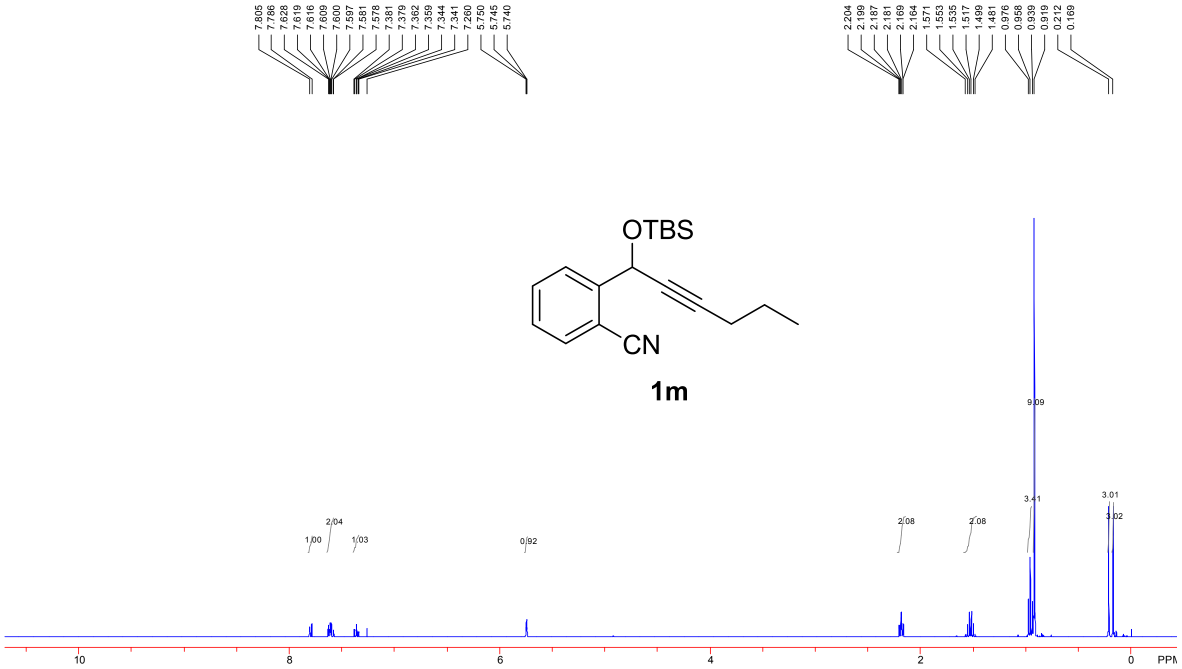

^{13}C NMR(100 MHz, $CDCl_3$, Me_4Si)

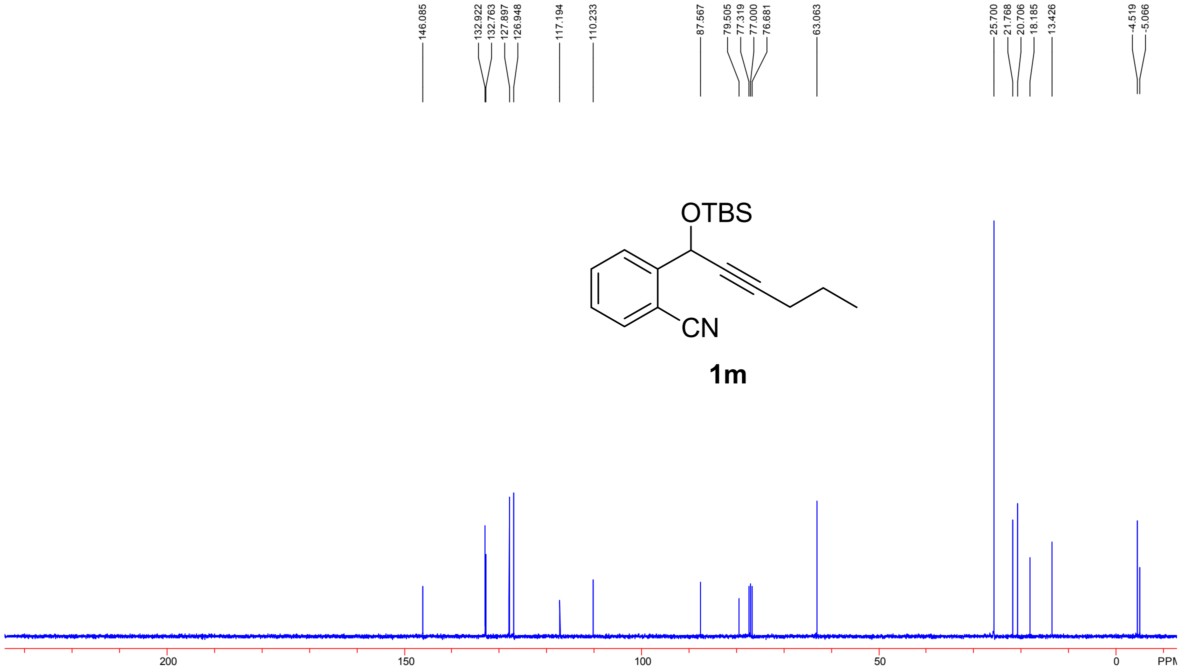

^{1}H NMR(400 MHz, C_6D_6, Me_4Si)

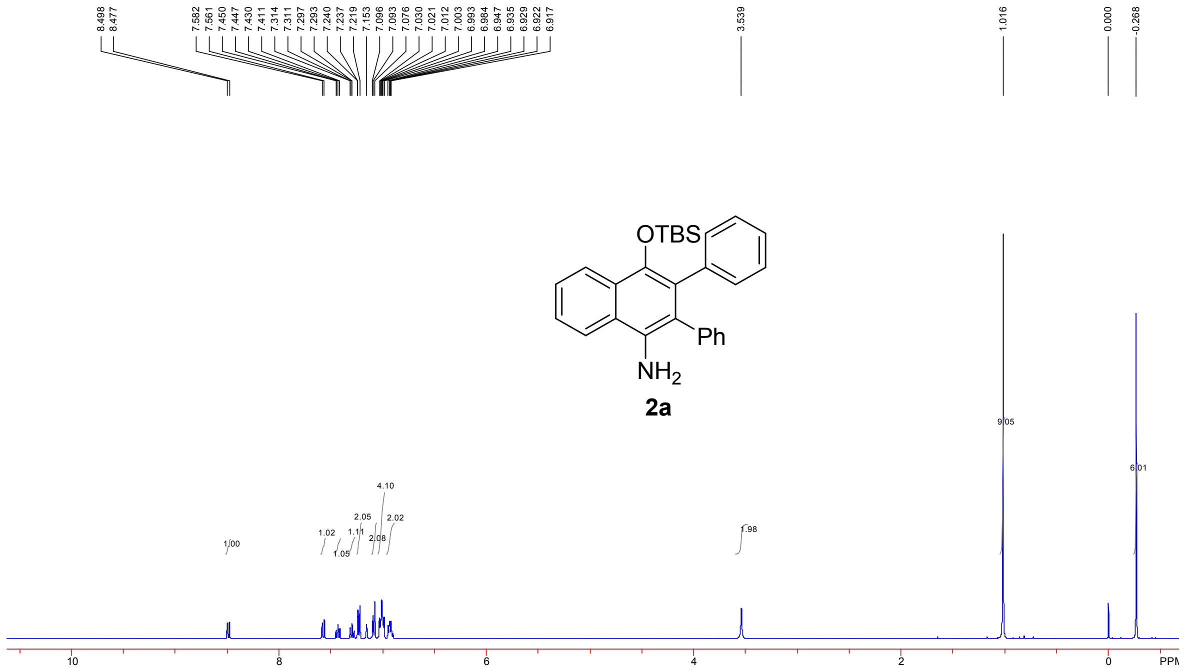

^{13}C NMR(100 MHz, C_6D_6, Me_4Si)

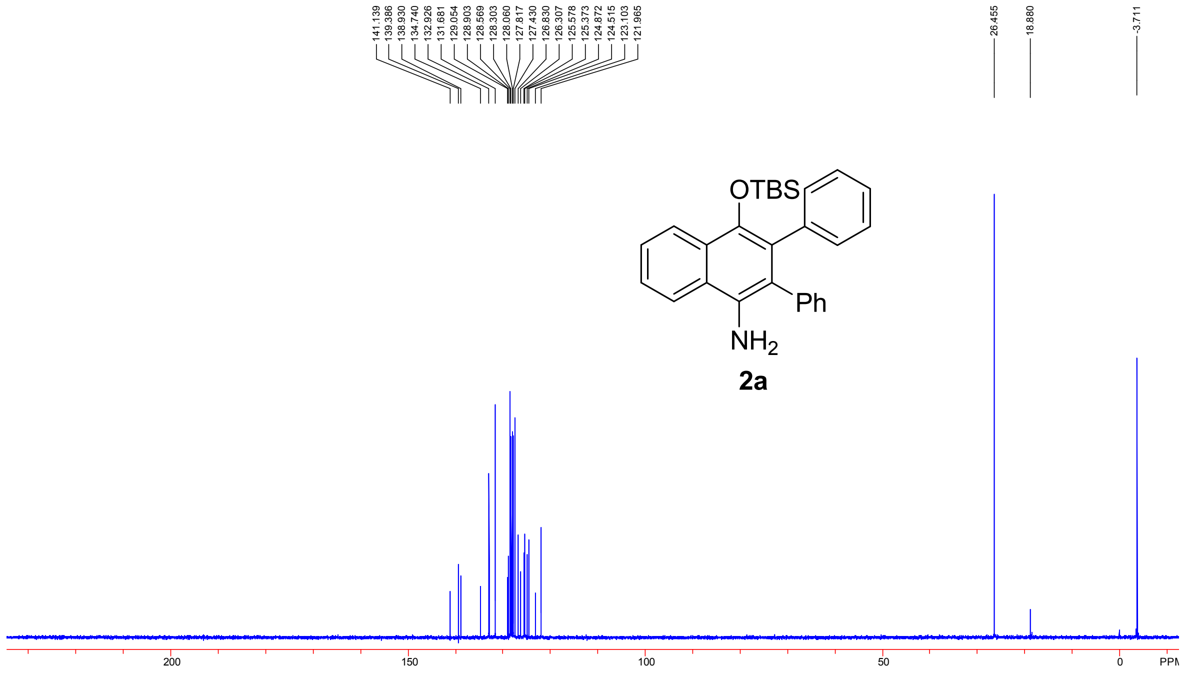

^{1}H NMR(400 MHz, C_6D_6, Me_4Si)

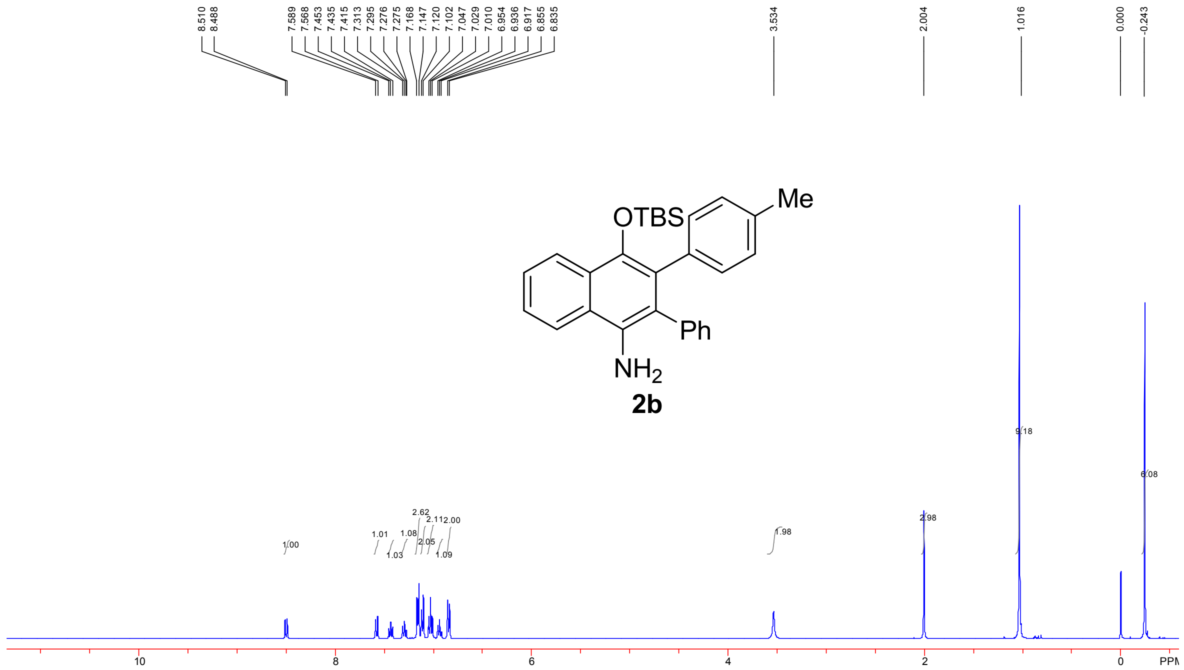

^{13}C NMR(100 MHz, C_6D_6, Me_4Si)

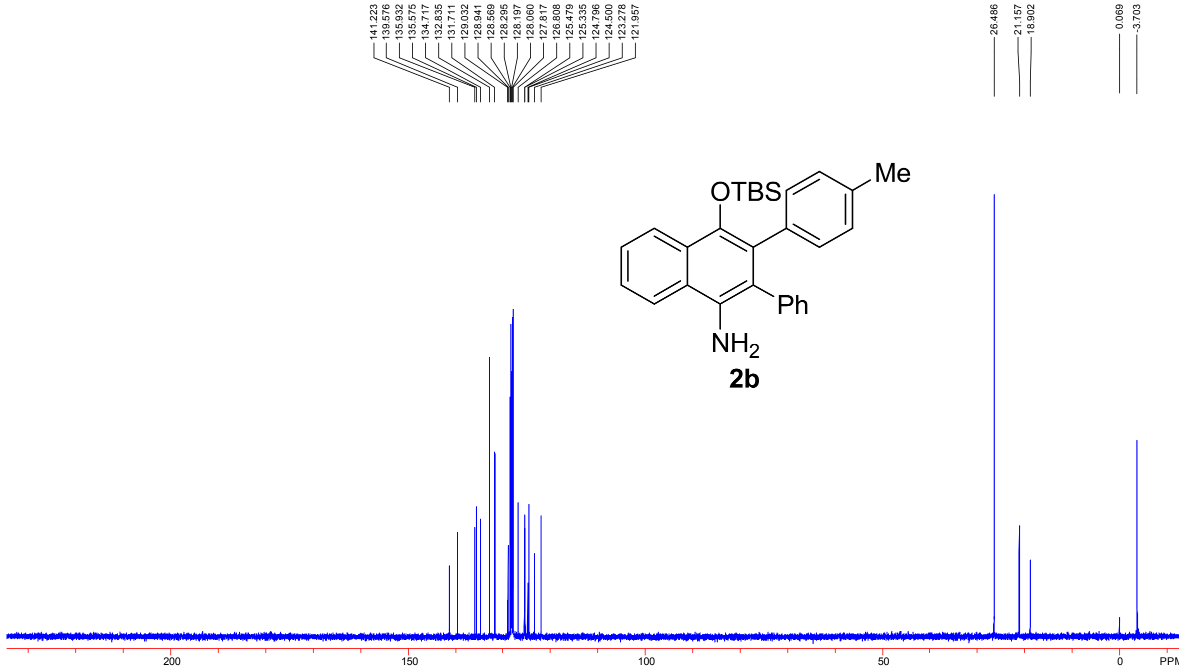

^{1}H NMR(400 MHz, C_6D_6, Me_4Si)

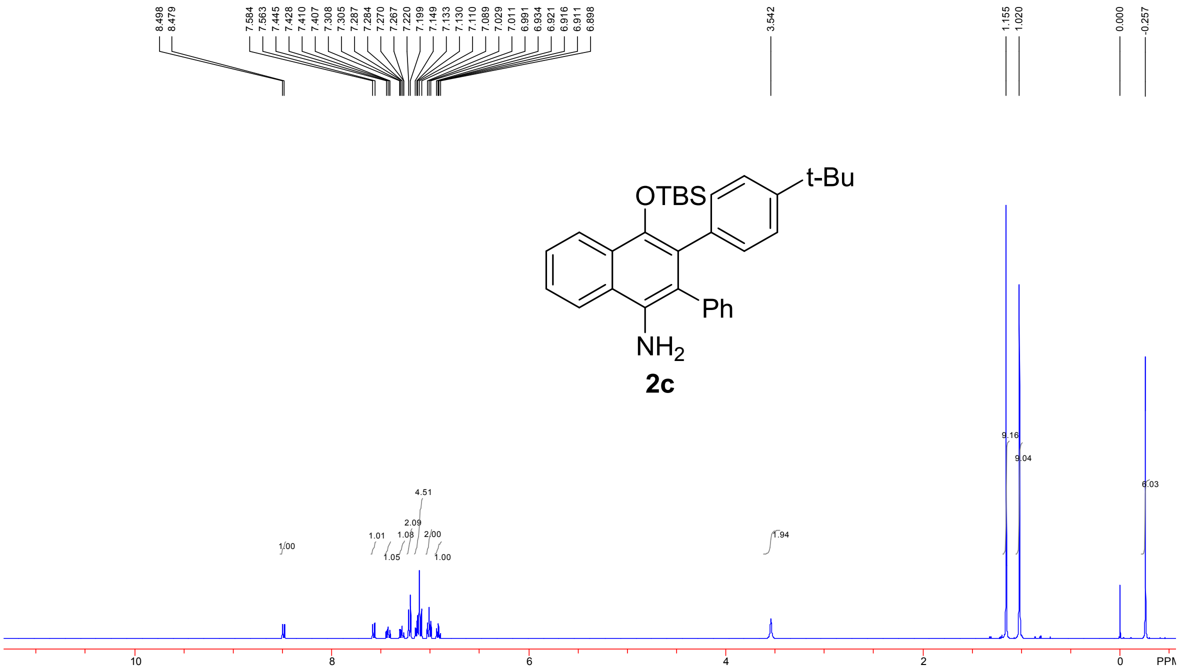

^{13}C NMR(100 MHz, C_6D_6, Me_4Si)

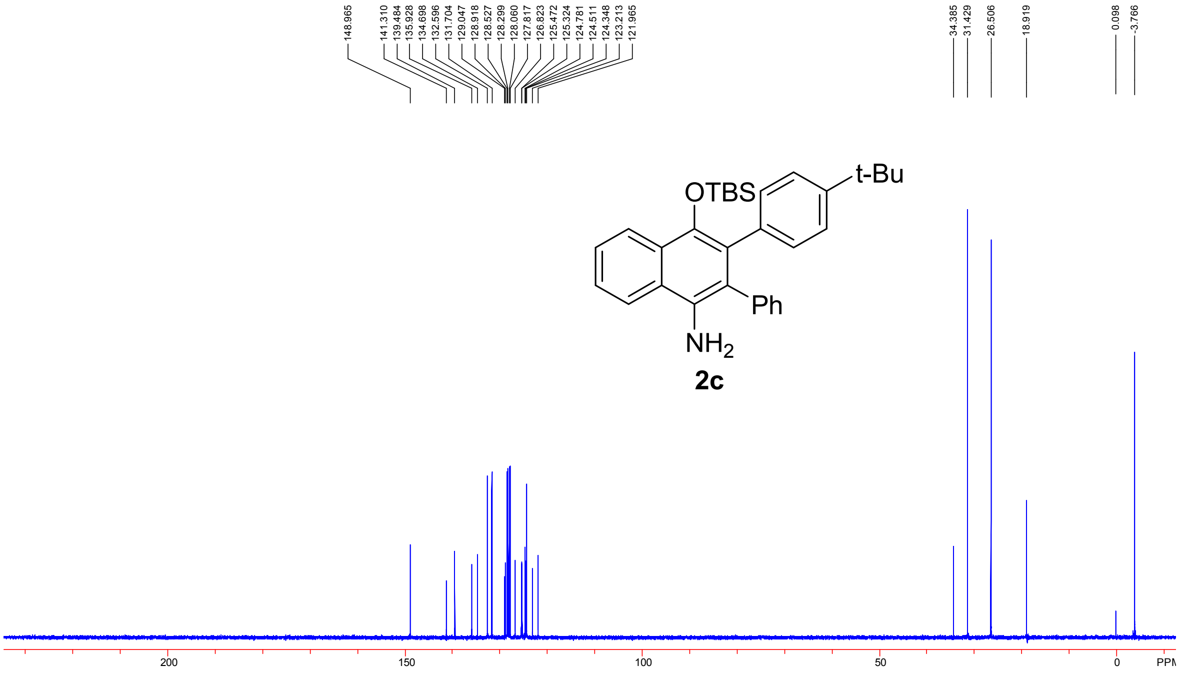

^{1}H NMR(400 MHz, C_6D_6, Me_4Si)

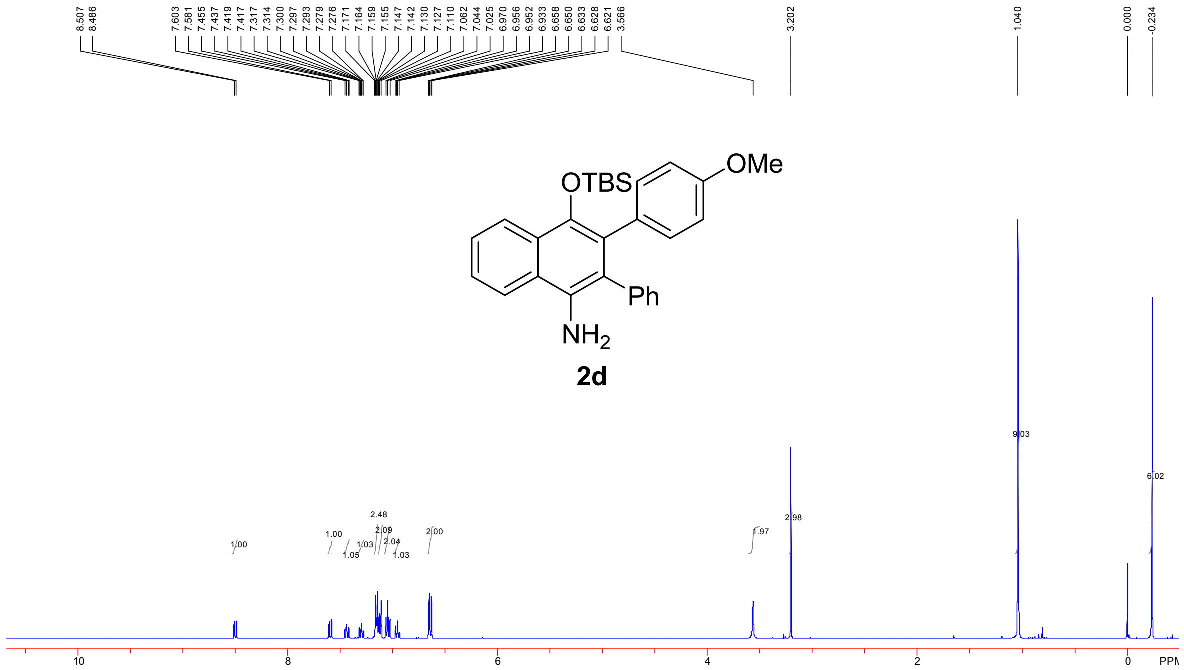

^{13}C NMR(100 MHz, C_6D_6, Me_4Si)

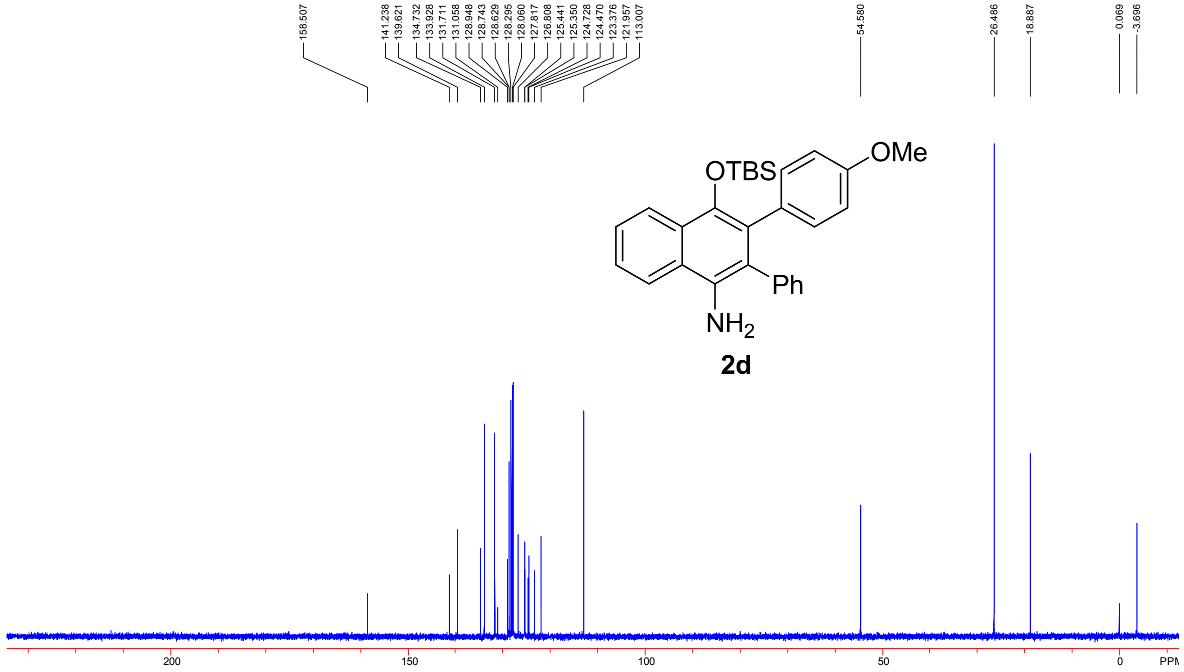

^{1}H NMR(400 MHz, C_6D_6, Me_4Si)

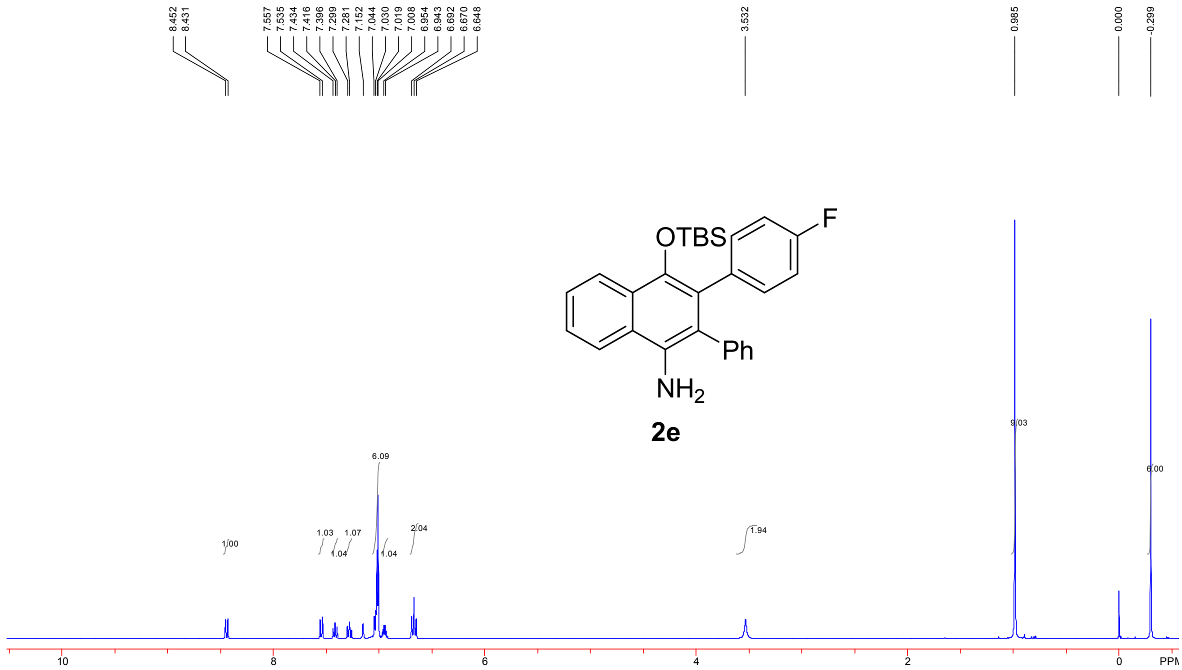

^{13}C NMR(100 MHz, C_6D_6, Me_4Si)

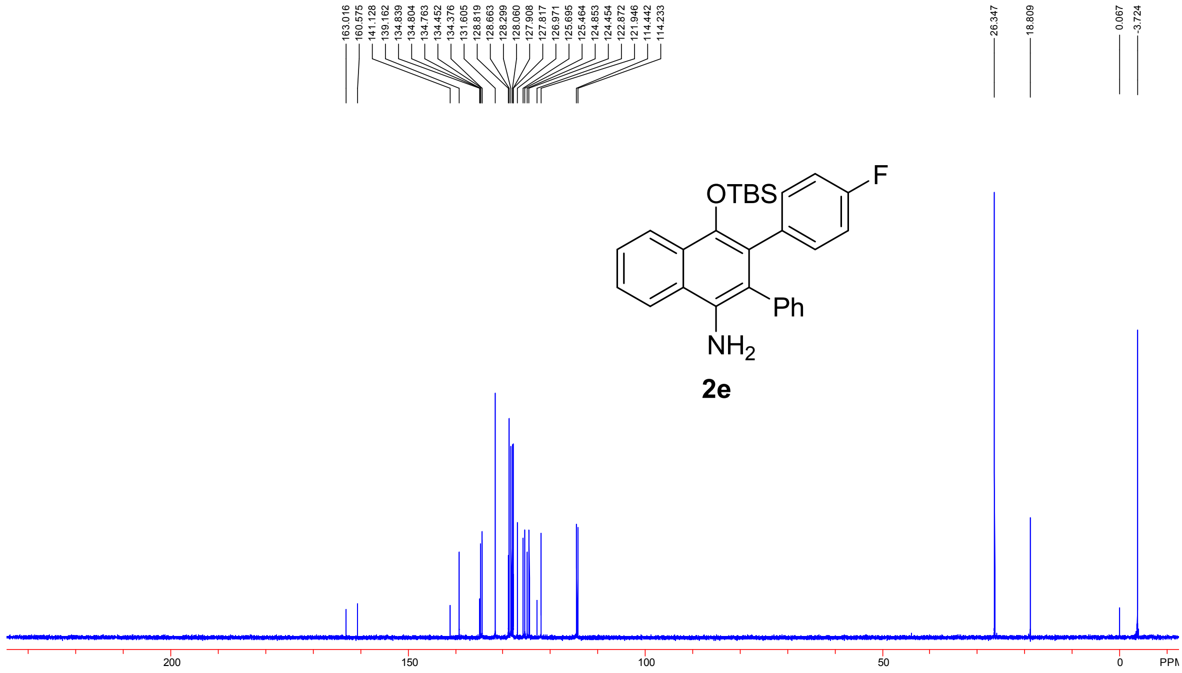

^{1}H NMR(400 MHz, C_6D_6, Me_4Si)

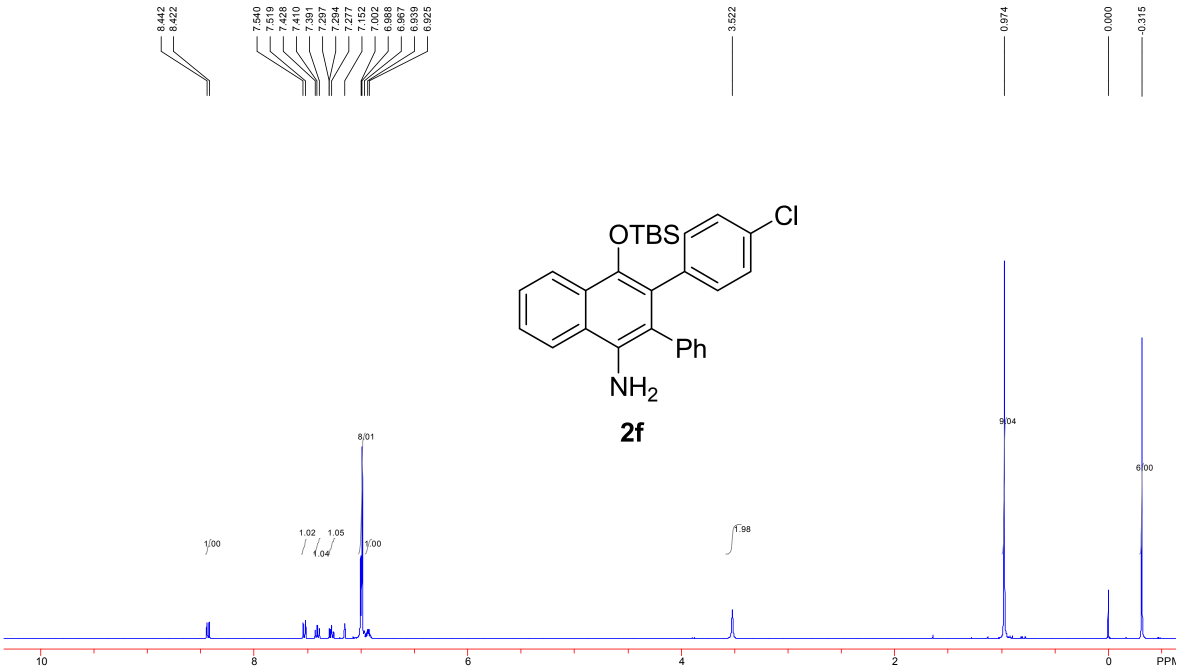

2f

^{13}C NMR(100 MHz, C_6D_6, Me_4Si)

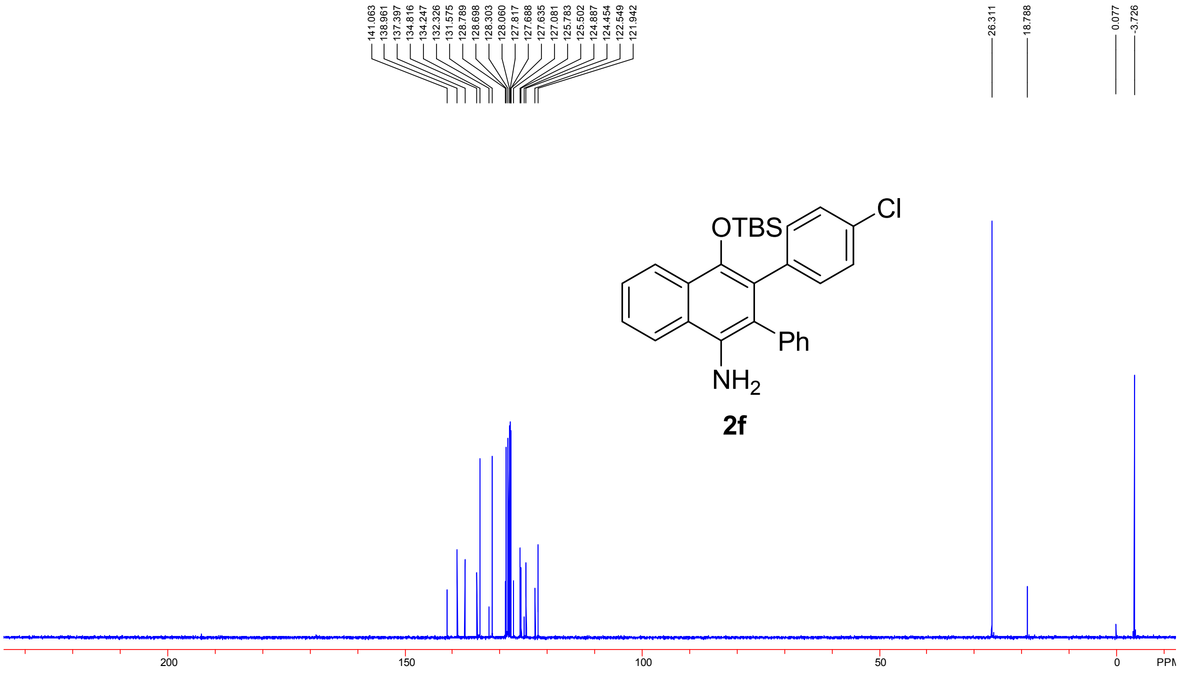

2f

^{1}H NMR(400 MHz, C_6D_6, Me_4Si)

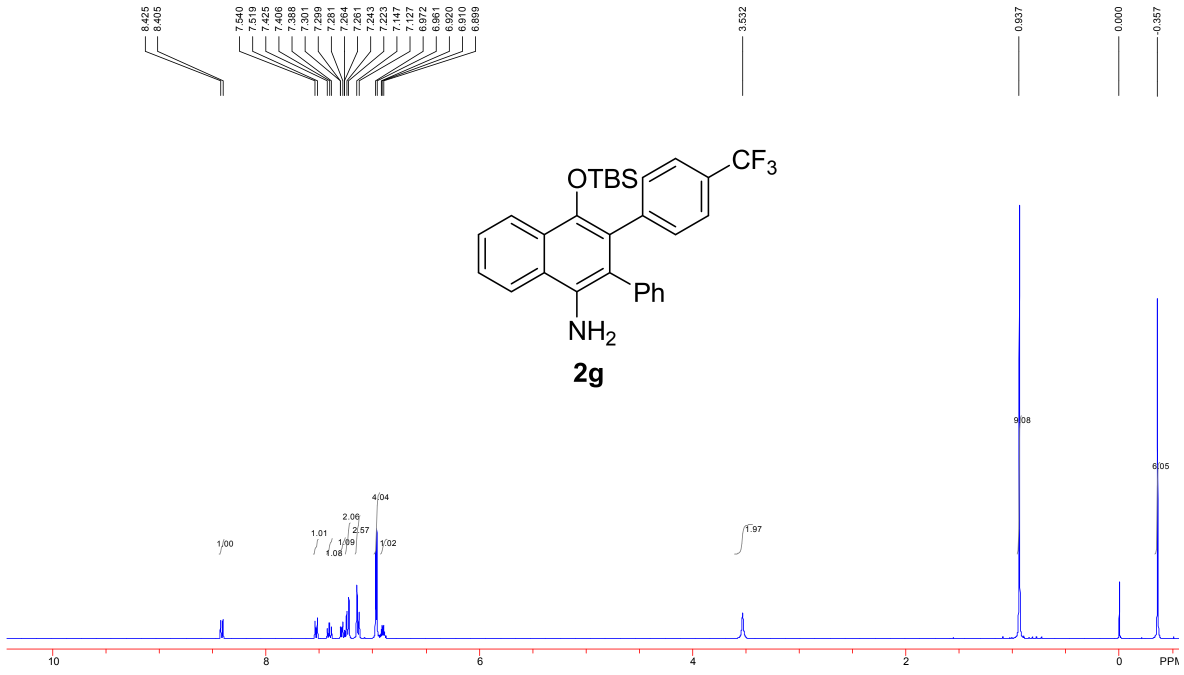

^{13}C NMR(100 MHz, C_6D_6, Me_4Si)

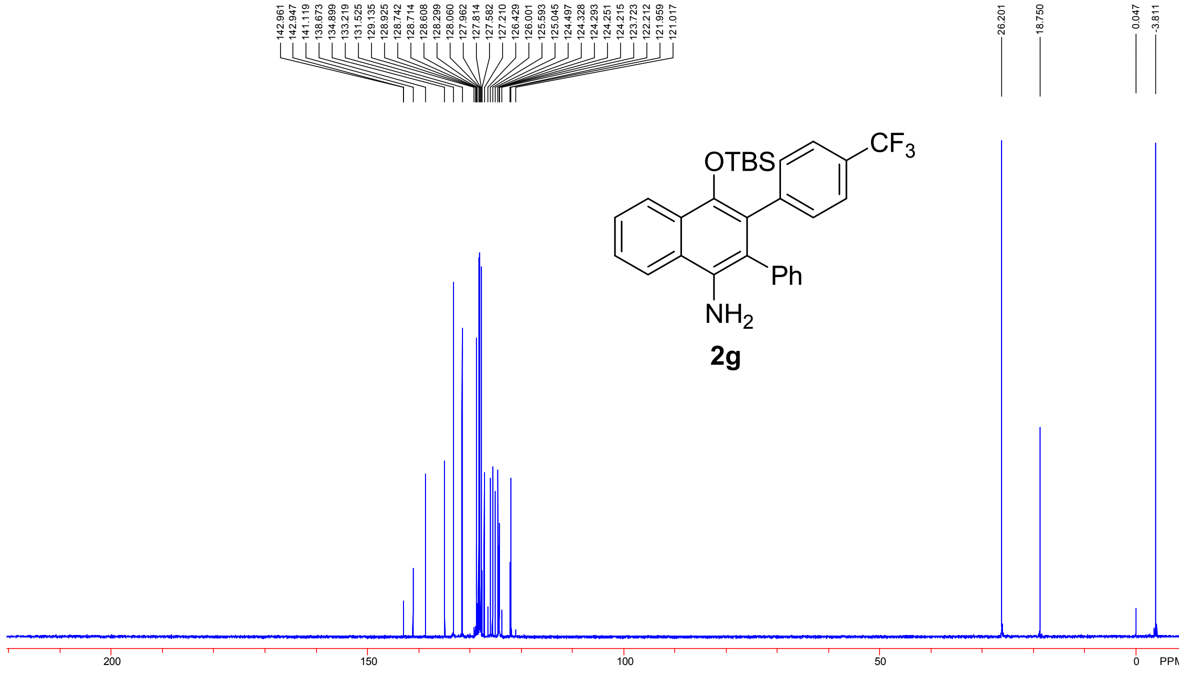

^{1}H NMR(400 MHz, C_6D_6, Me_4Si)

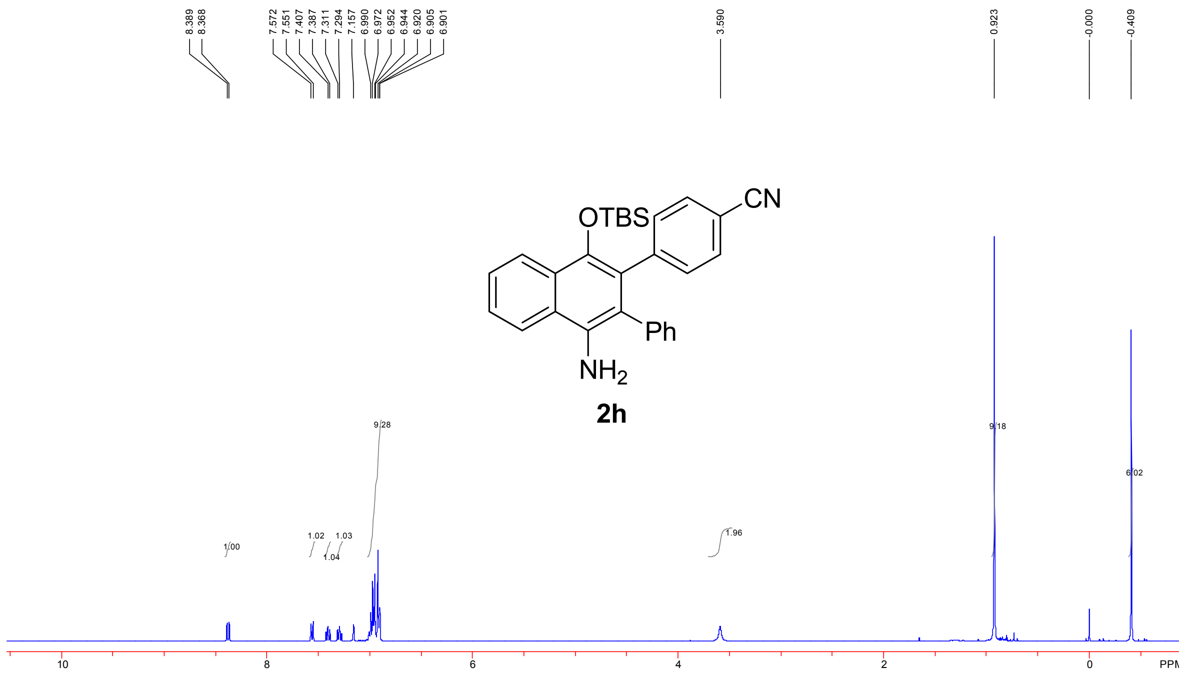

^{13}C NMR(100 MHz, C_6D_6, Me_4Si)

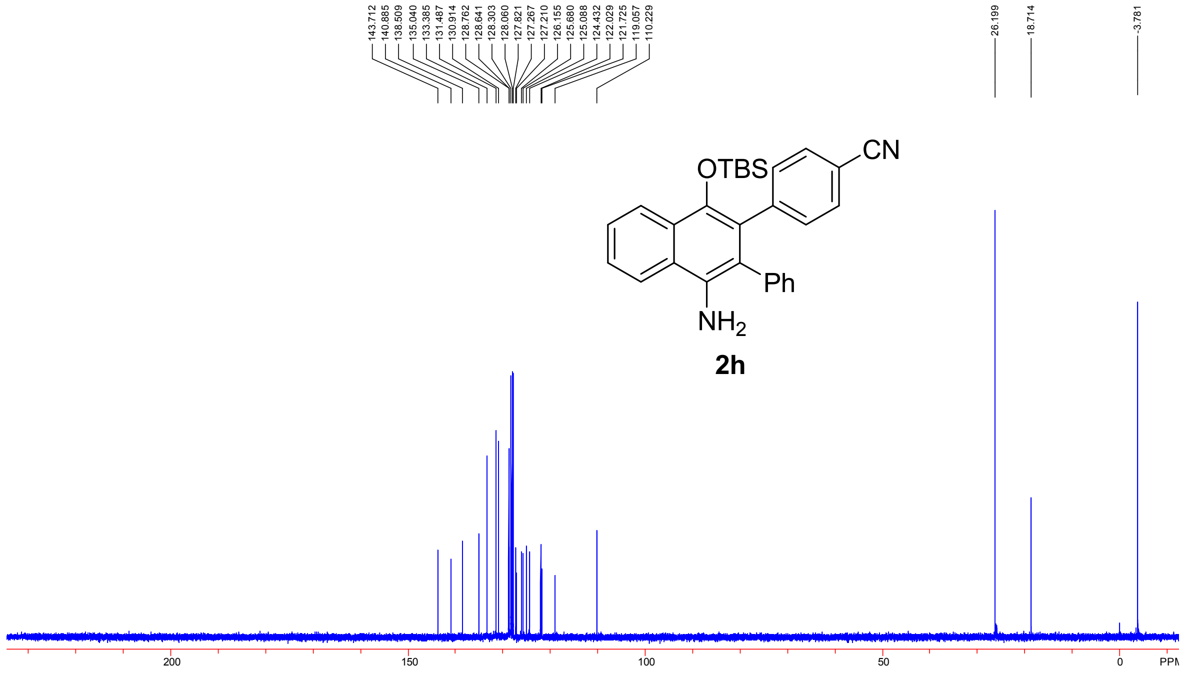

^{1}H NMR(400 MHz, C_6D_6, Me_4Si)

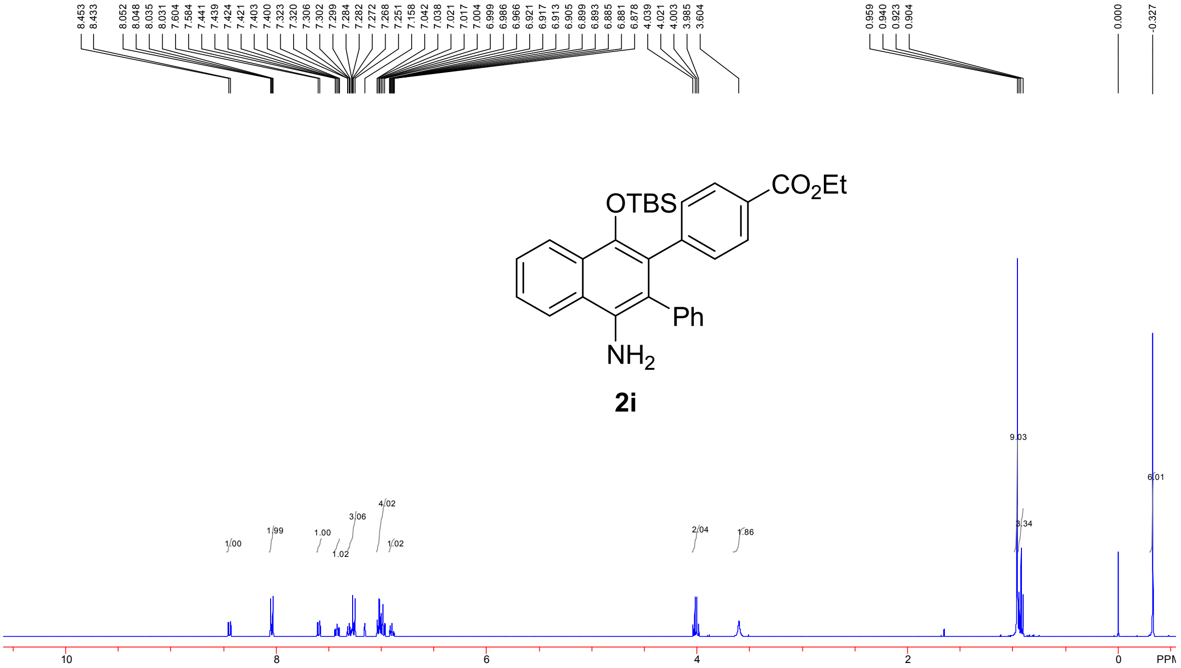

^{13}C NMR(100 MHz, C_6D_6, Me_4Si)

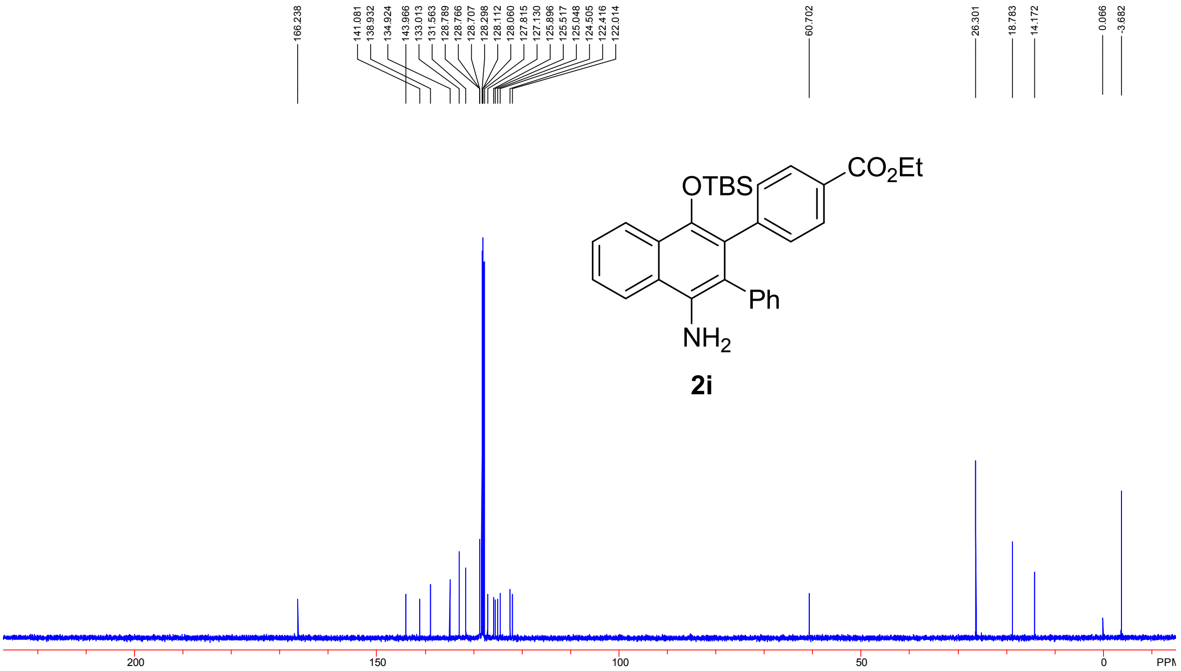

^{1}H NMR(400 MHz, C_6D_6, Me_4Si)

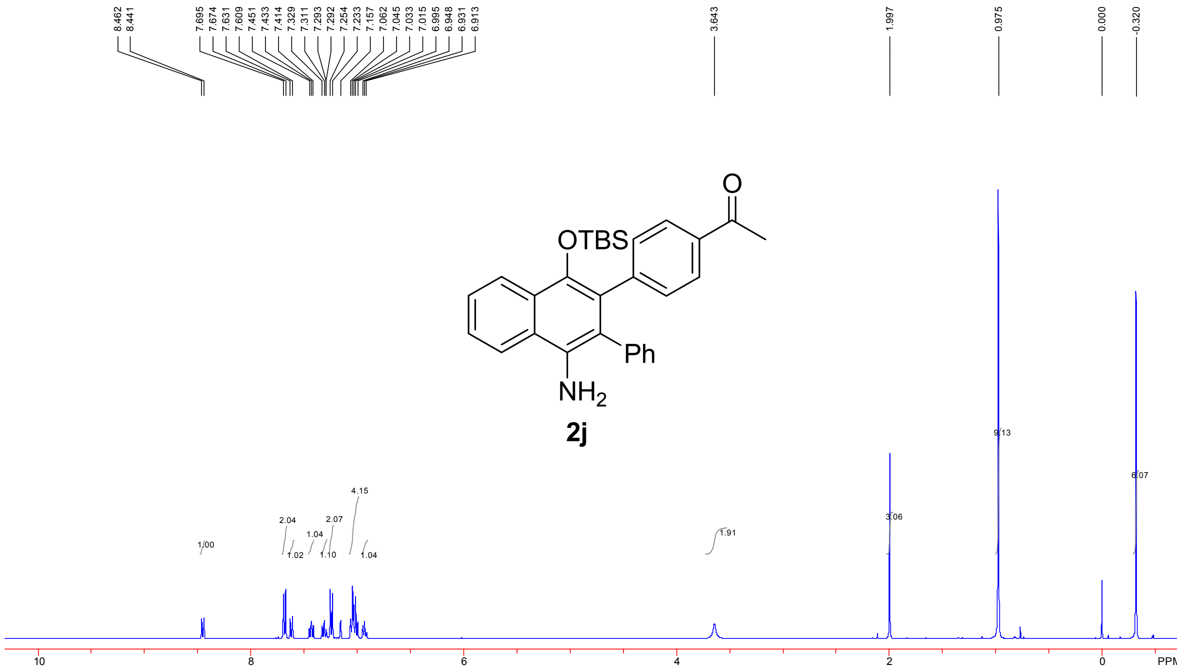

^{13}C NMR(100 MHz, C_6D_6, Me_4Si)

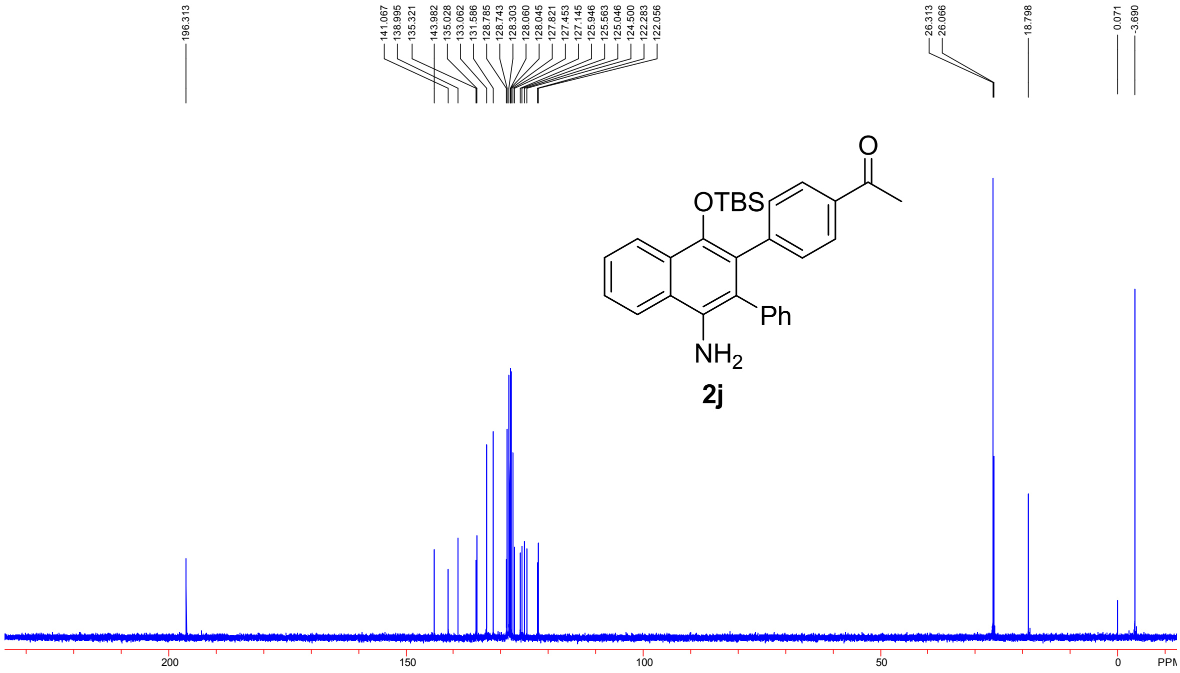

^{1}H NMR(400 MHz, C_6D_6, Me_4Si)

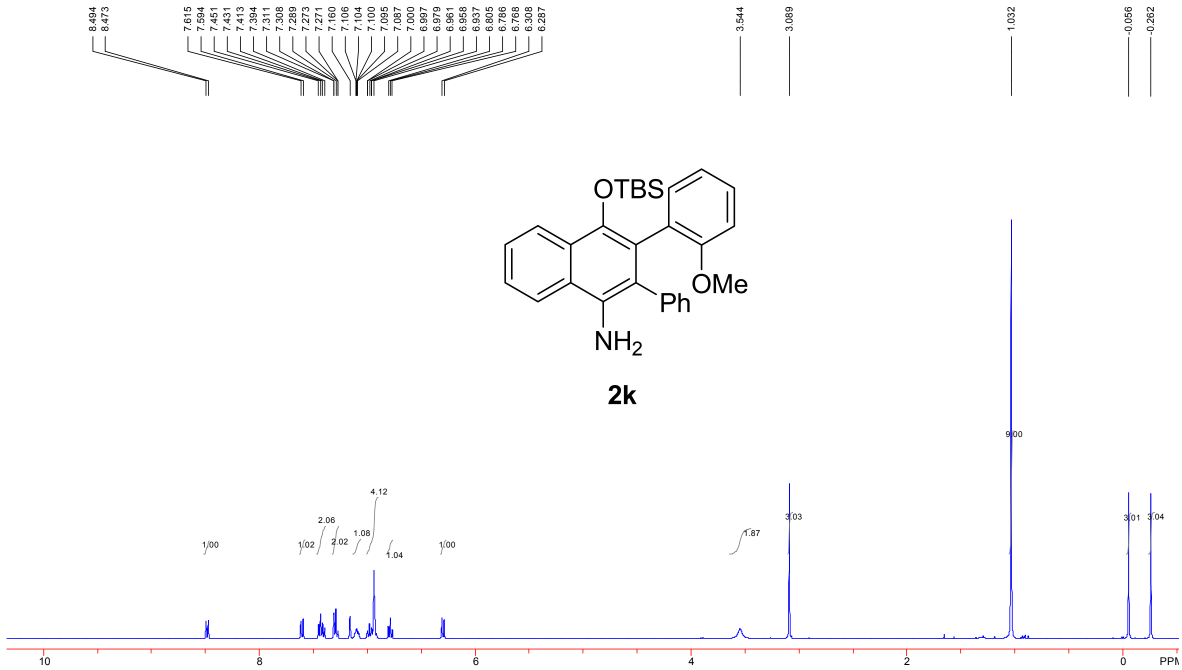

^{13}C NMR(100 MHz, C_6D_6, Me_4Si)

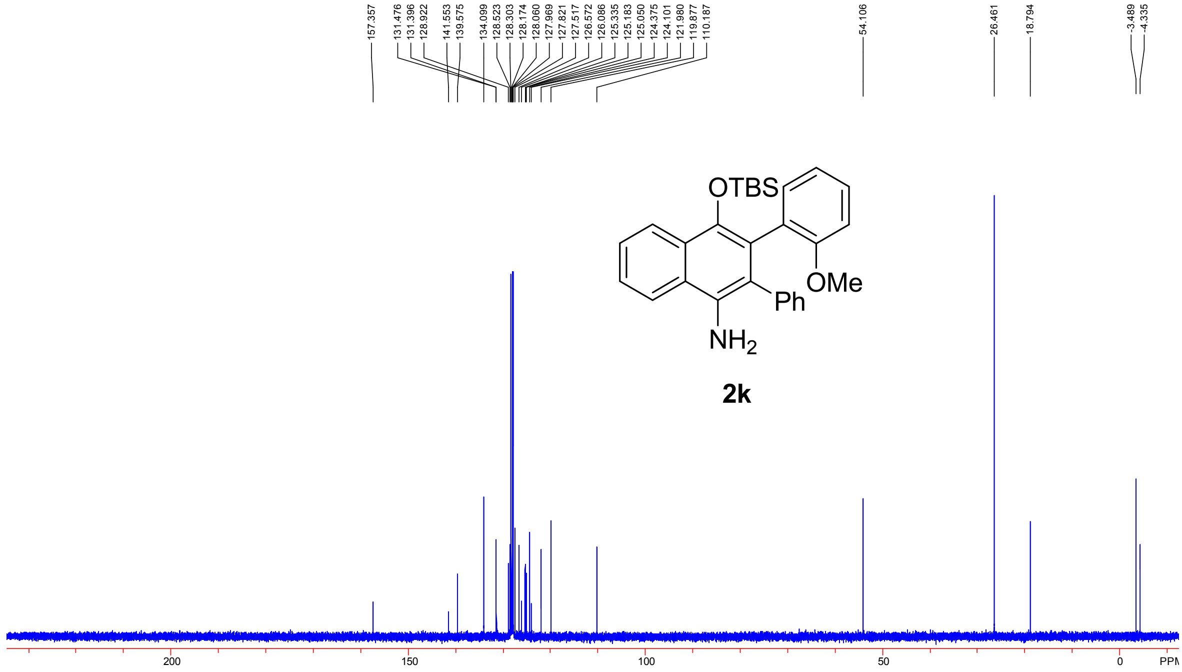

^{1}H NMR(400 MHz, C_6D_6, Me_4Si)

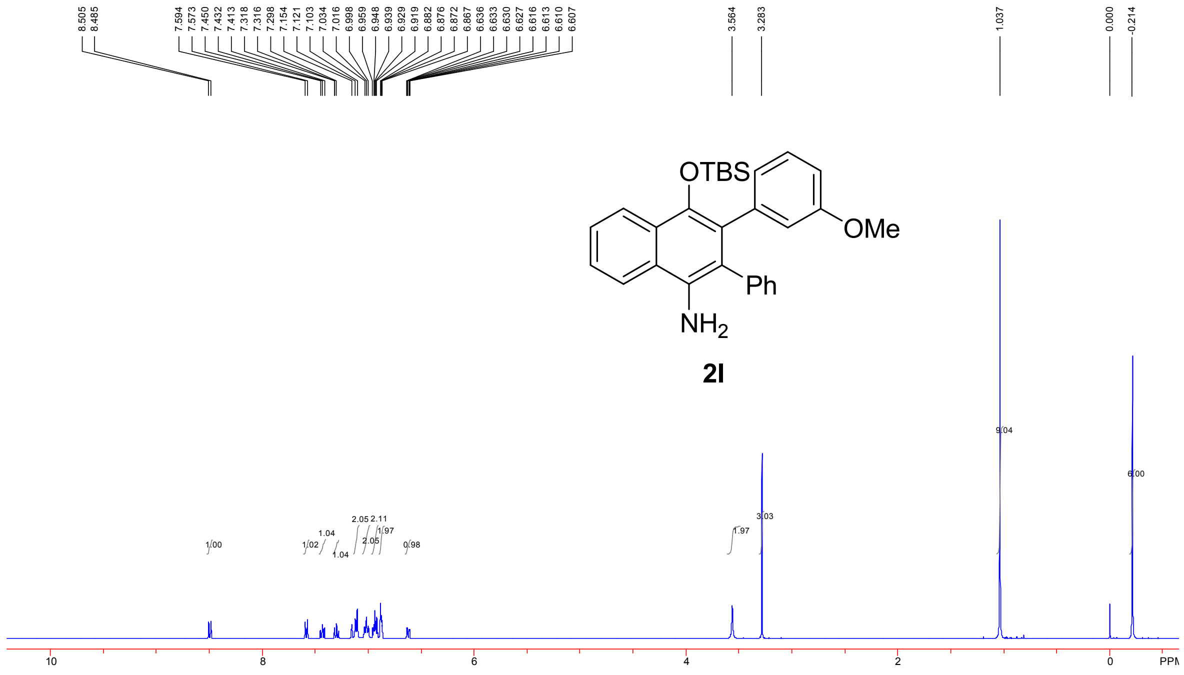

^{13}C NMR(100 MHz, C_6D_6, Me_4Si)

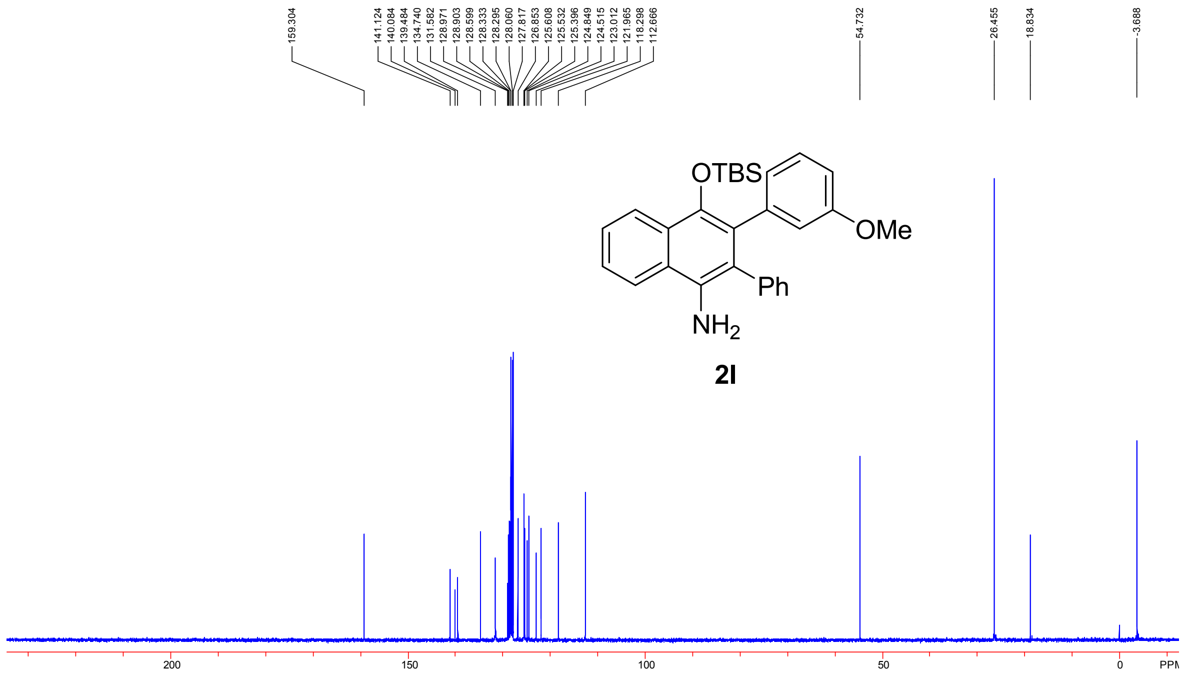

^{1}H NMR(400 MHz, C_6D_6, Me_4Si)

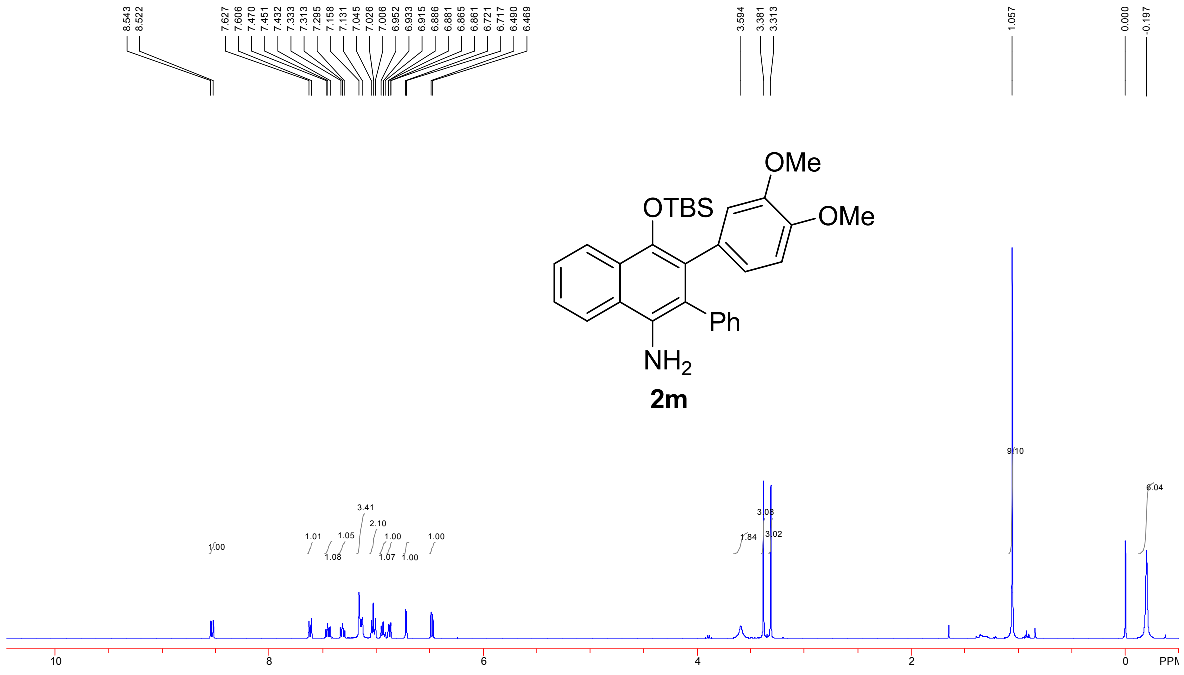

^{13}C NMR(100 MHz, C_6D_6, Me_4Si)

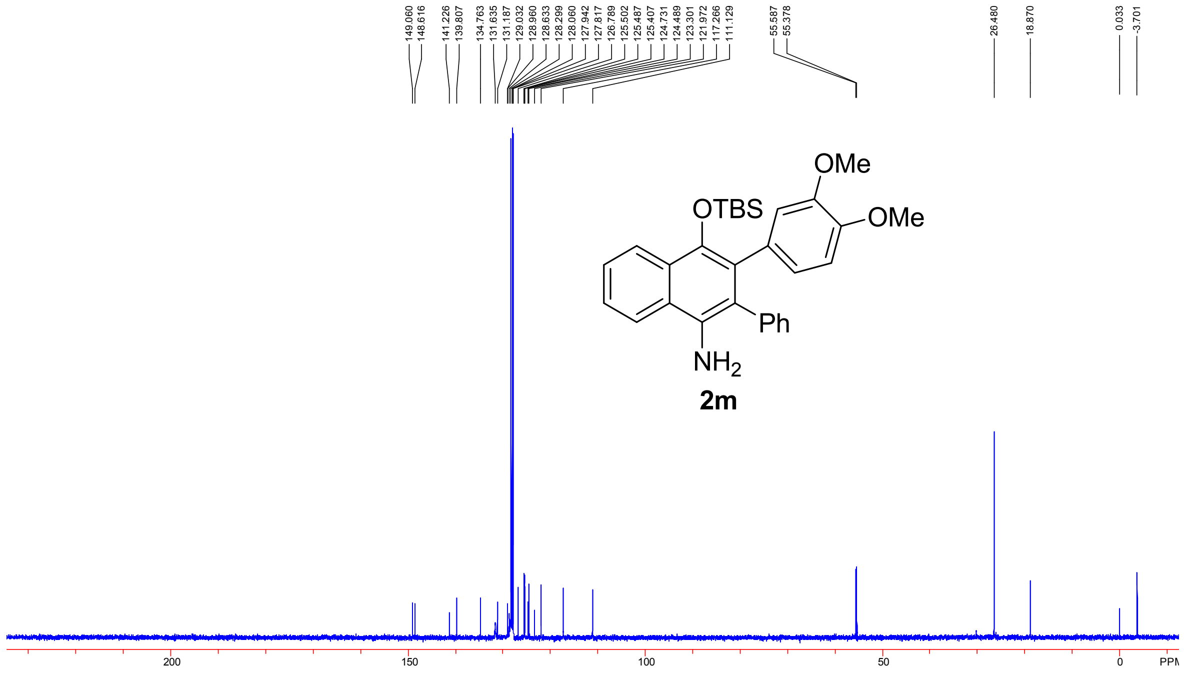

^{1}H NMR(400 MHz, C_6D_6, Me_4Si)

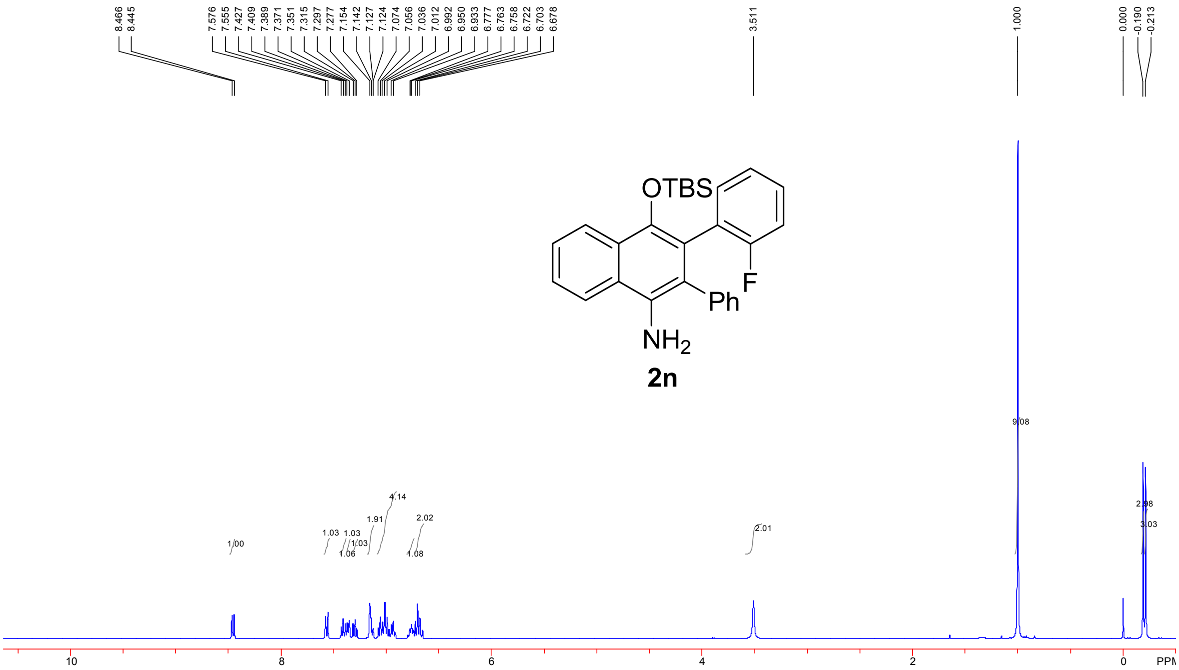

^{13}C NMR(100 MHz, C_6D_6, Me_4Si)

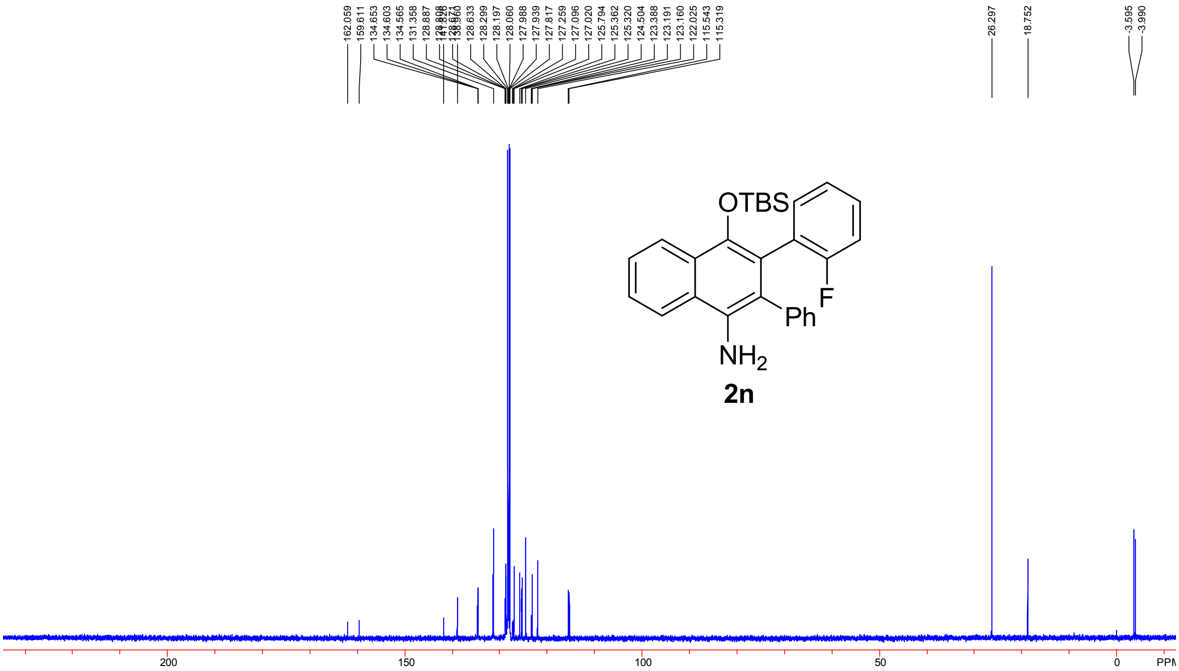

^{1}H NMR(400 MHz, C_6D_6, Me_4Si)

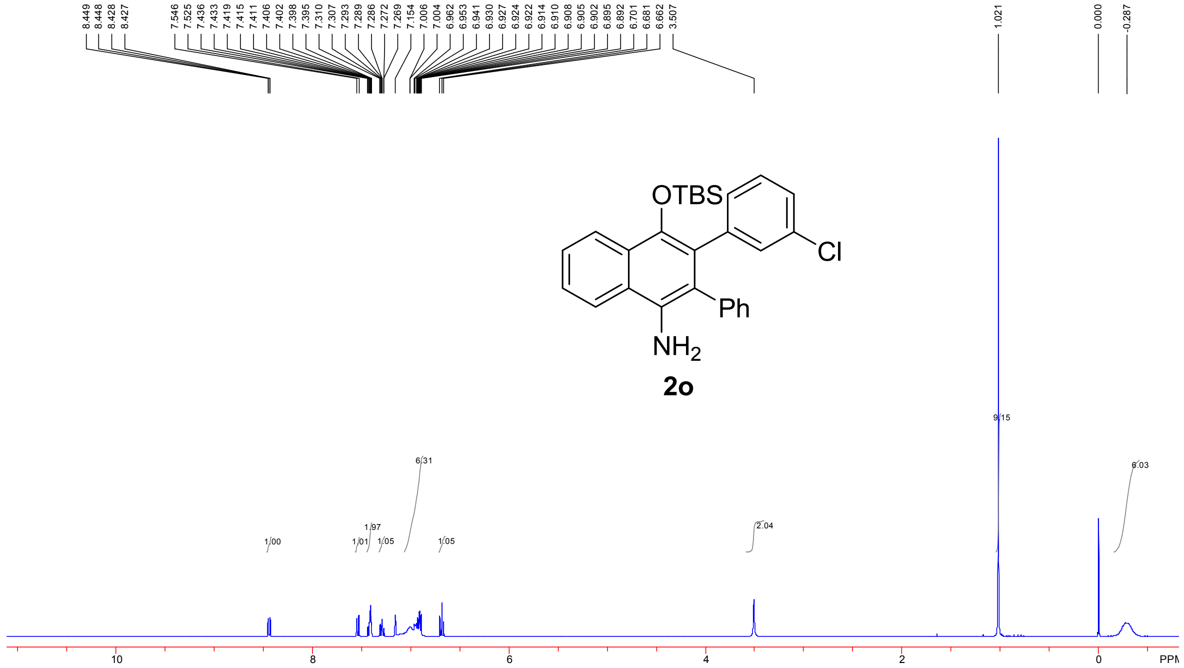

^{13}C NMR(100 MHz, C_6D_6, Me_4Si)

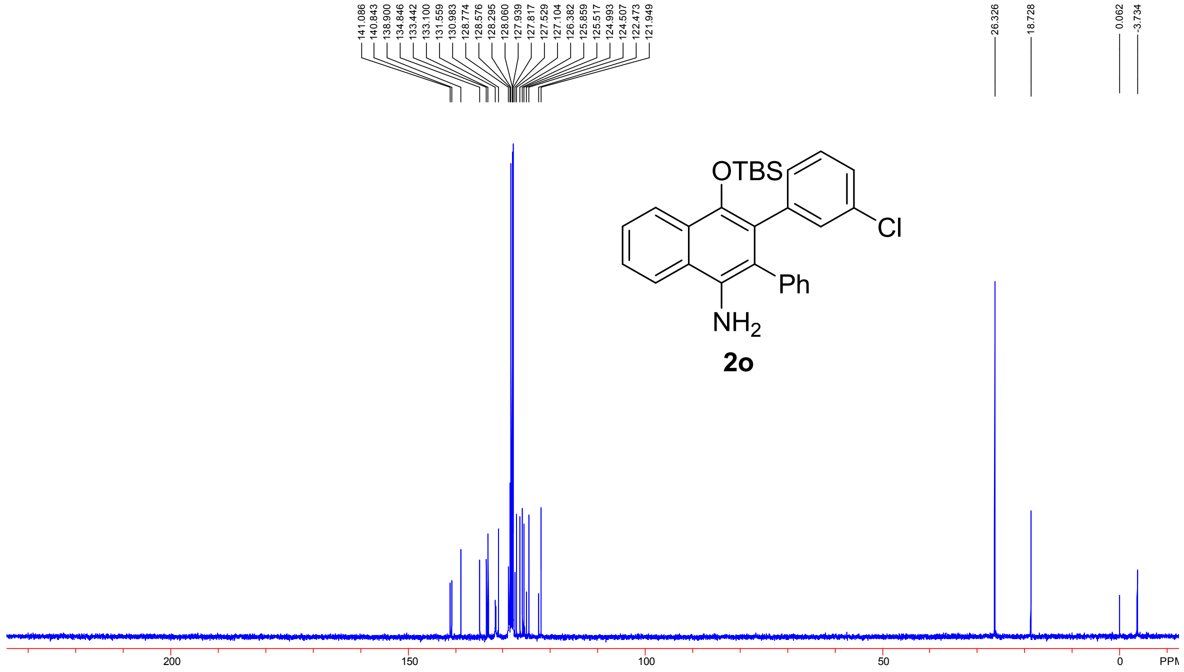

^{1}H NMR(400 MHz, C_6D_6, Me_4Si)

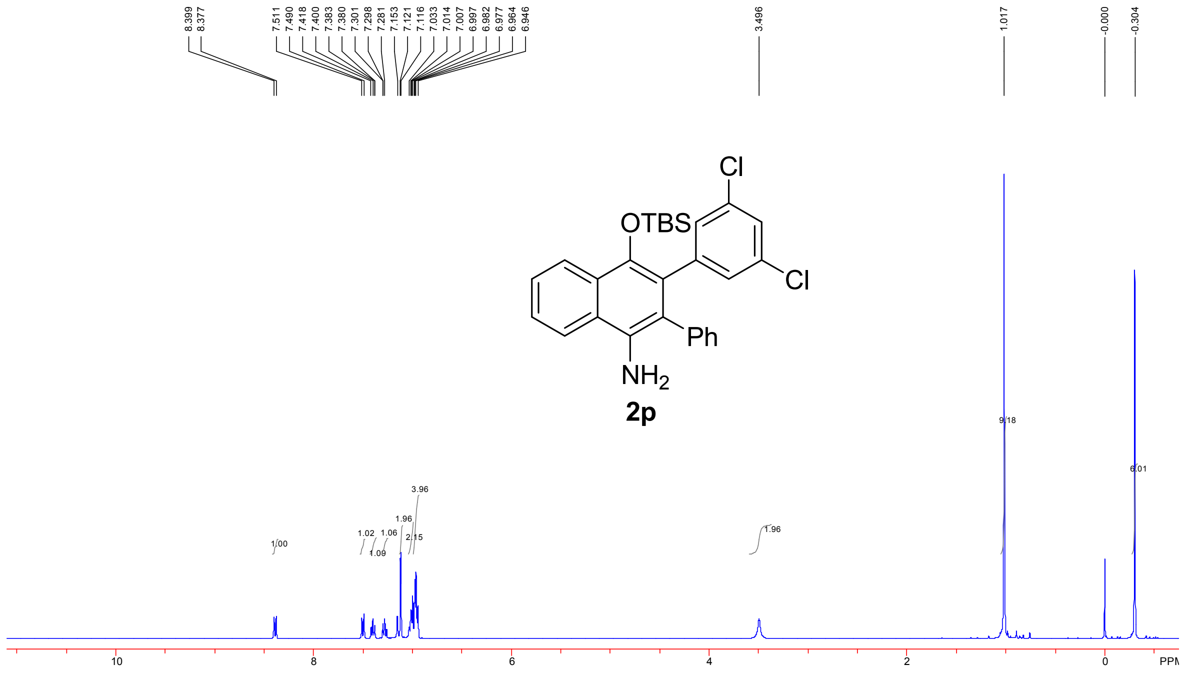

^{13}C NMR(100 MHz, C_6D_6, Me_4Si)

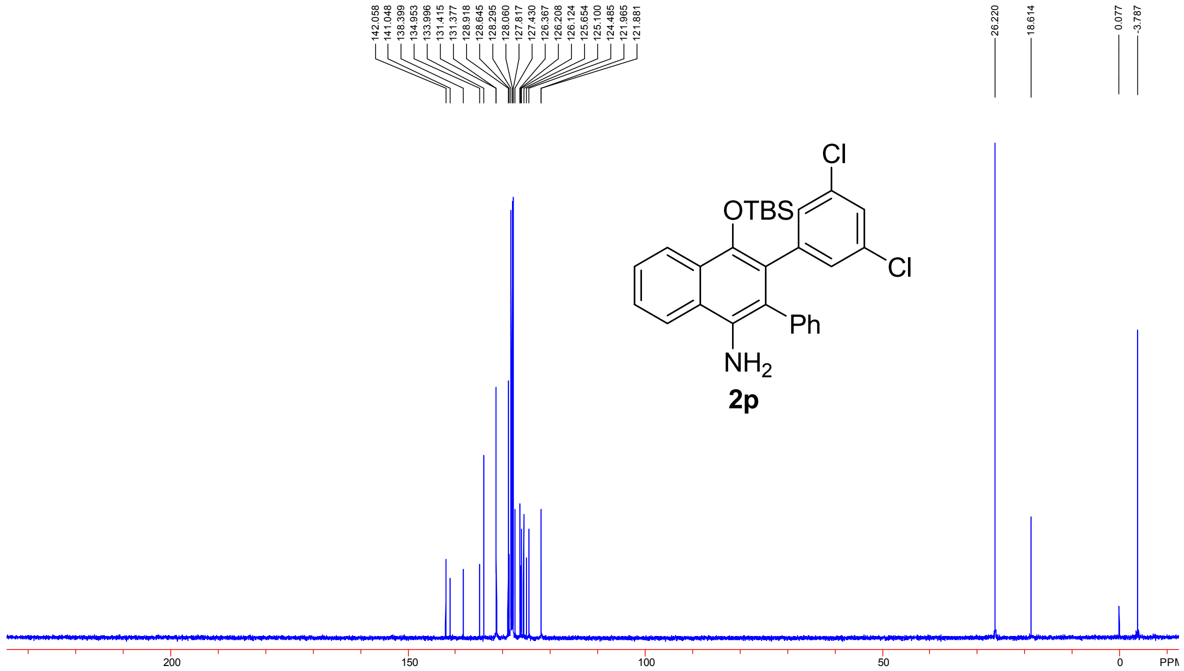

^{1}H NMR(400 MHz, C_6D_6, Me_4Si)

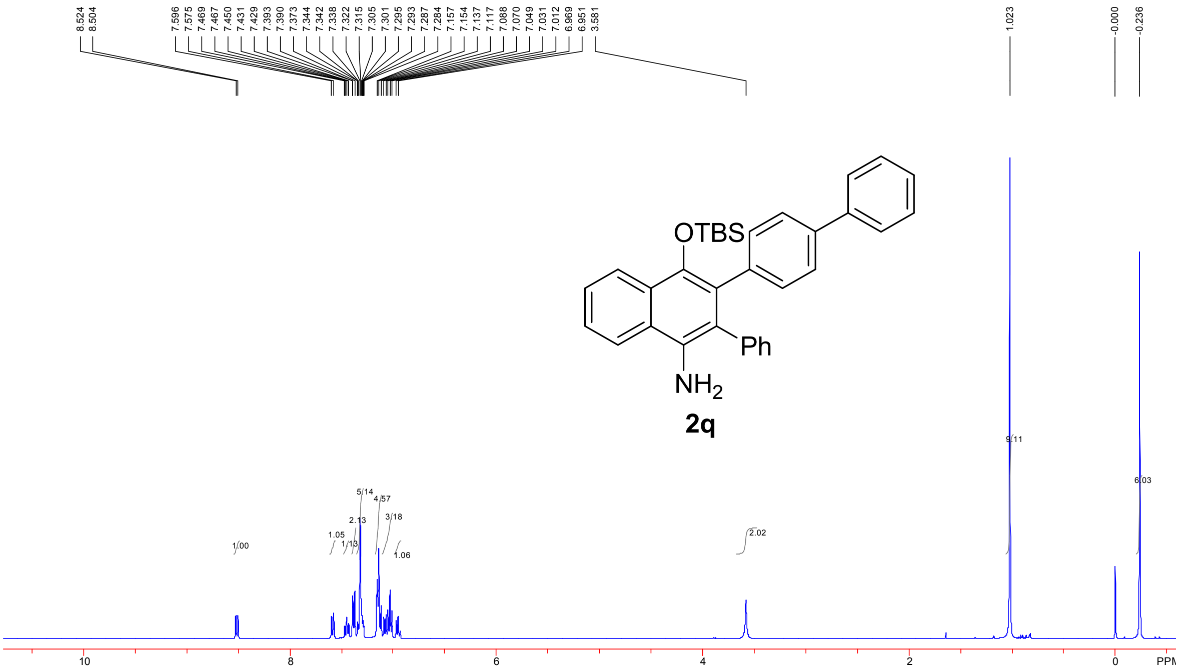

^{13}C NMR(100 MHz, C_6D_6, Me_4Si)

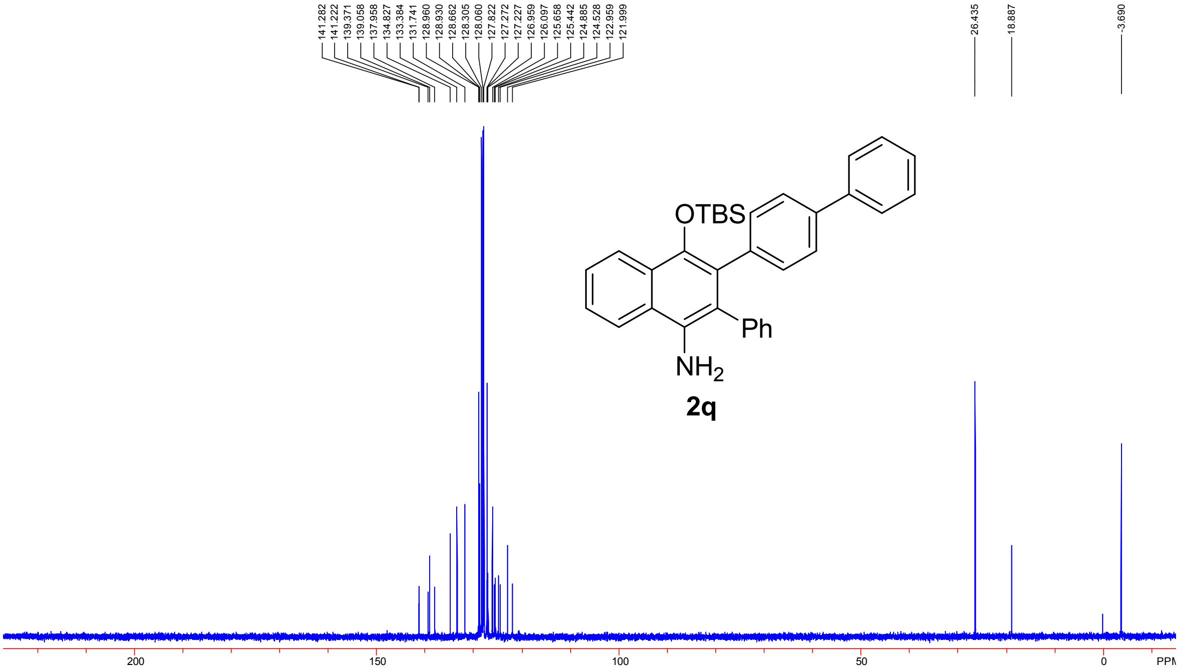

^{1}H NMR(400 MHz, C_6D_6, Me_4Si)

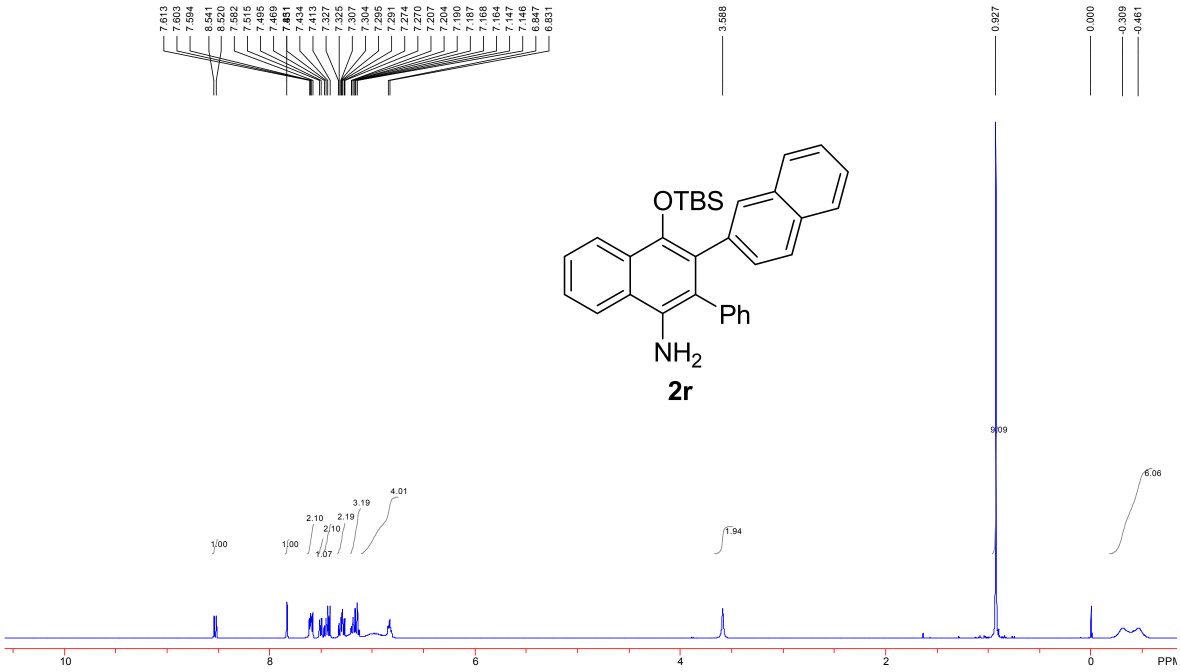

^{13}C NMR(100 MHz, C_6D_6, Me_4Si)

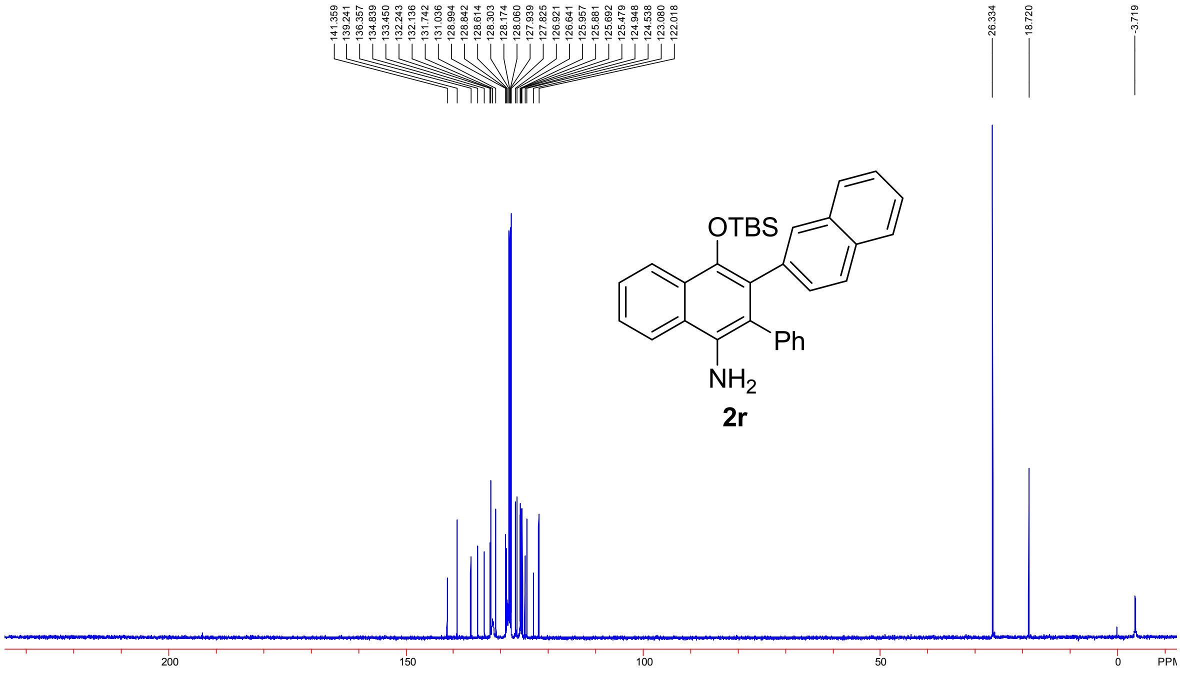

^{1}H NMR(400 MHz, $CDCl_3$, Me_4Si)

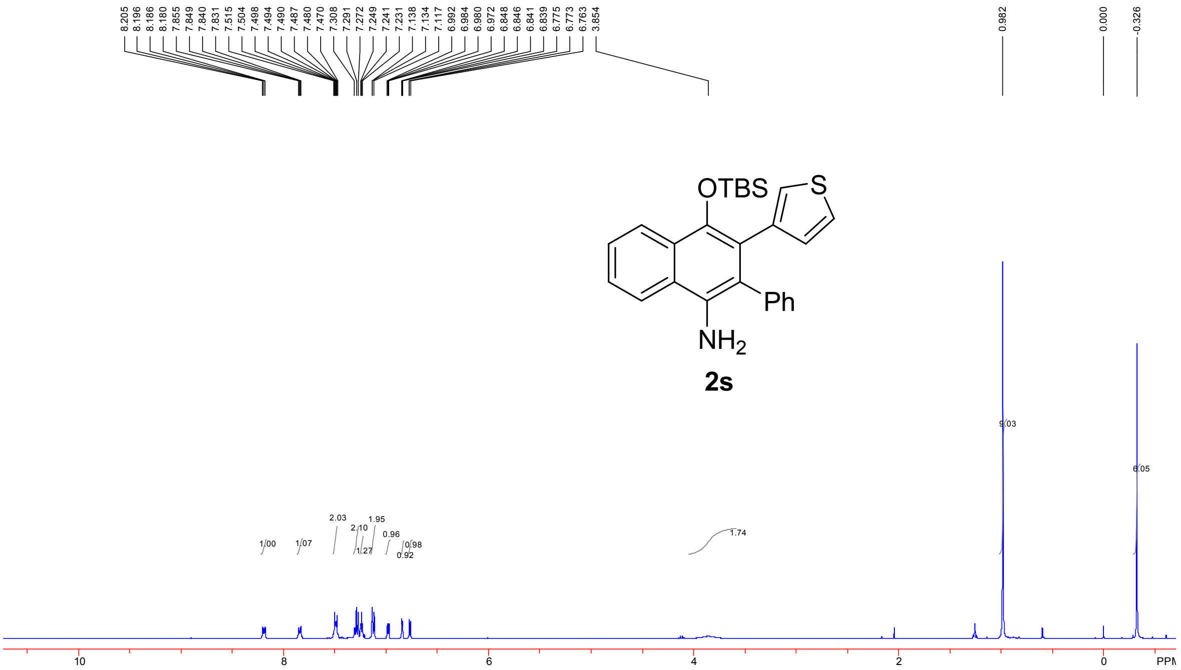

^{13}C NMR(100 MHz, $CDCl_3$, Me_4Si)

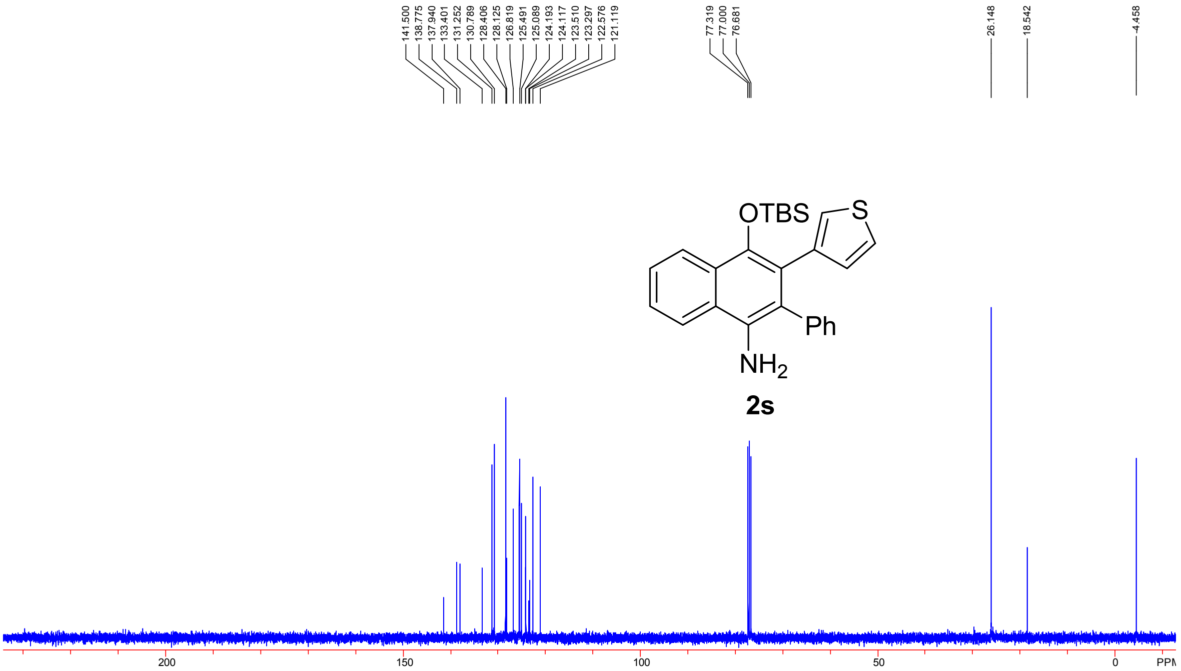

^{1}H NMR(400 MHz, C_6D_6, Me_4Si)

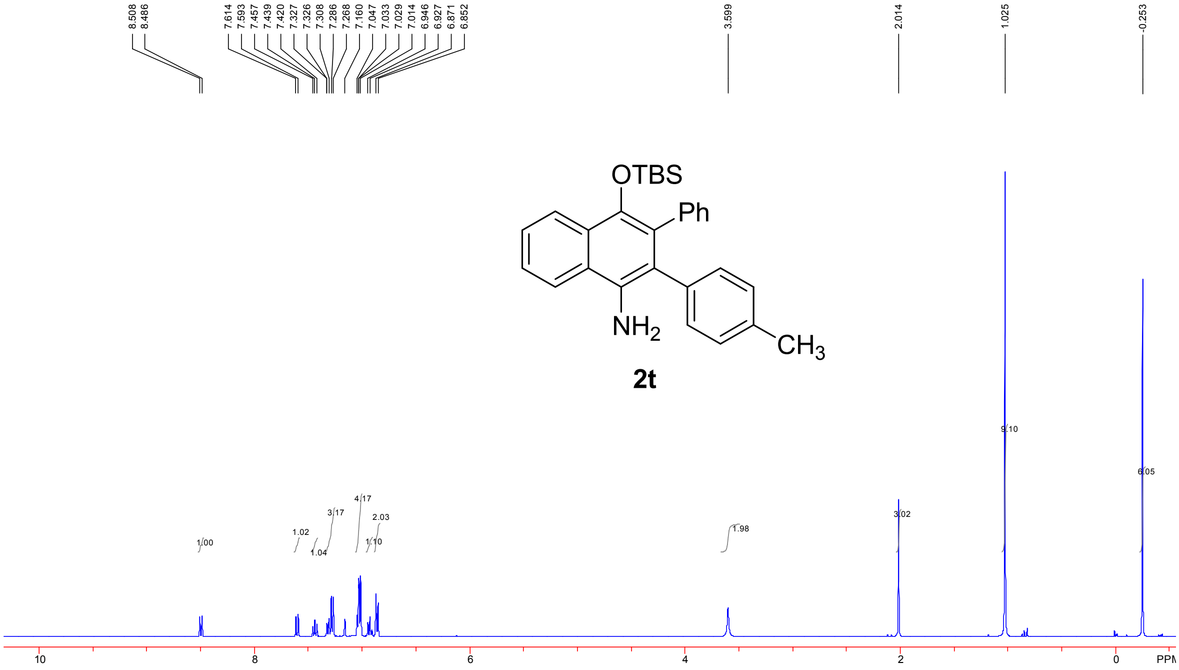

^{13}C NMR(100 MHz, C_6D_6, Me_4Si)

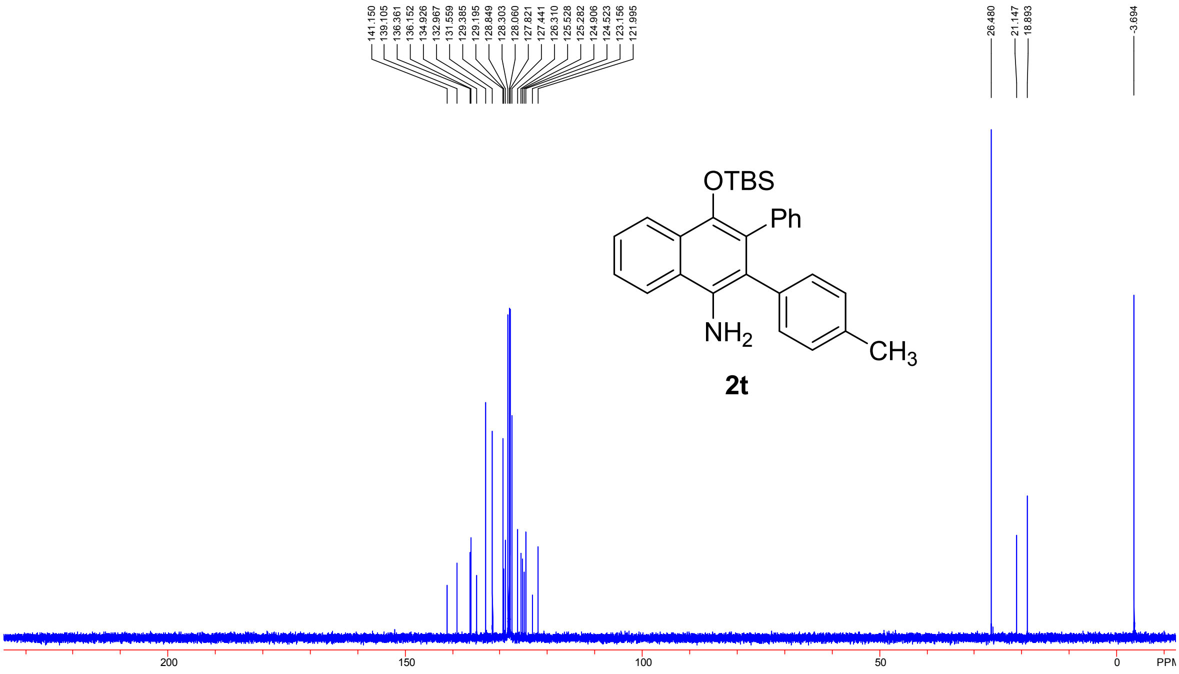

^{1}H NMR(400 MHz, C_6D_6, Me_4Si)

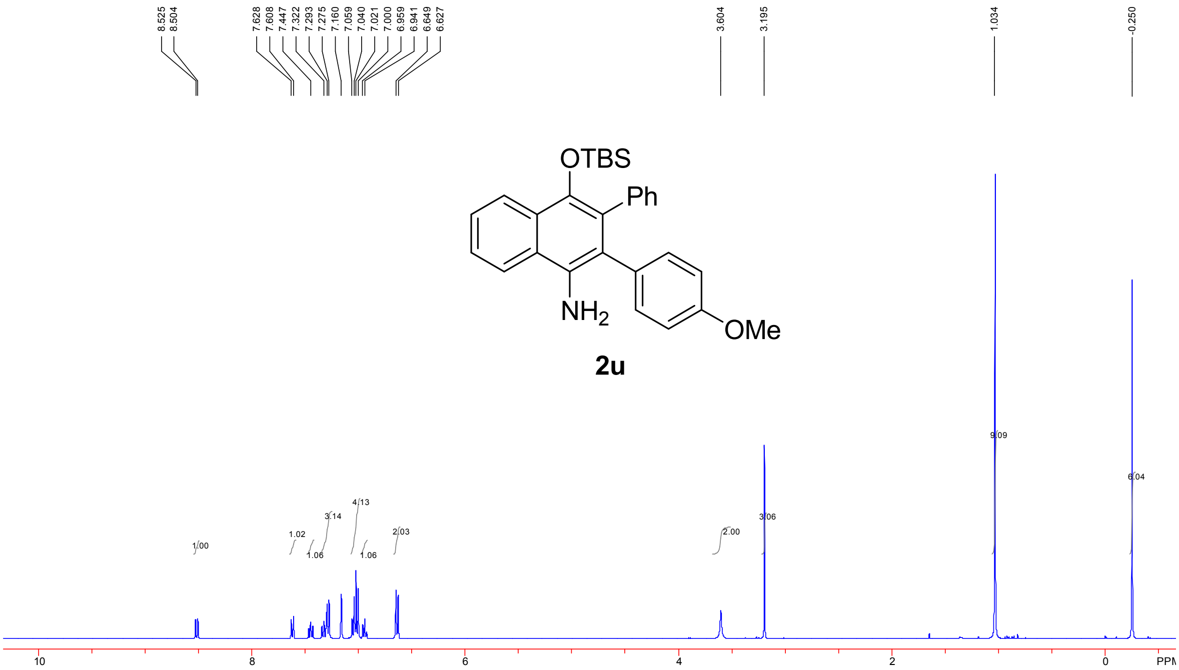

^{13}C NMR(100 MHz, C_6D_6, Me_4Si)

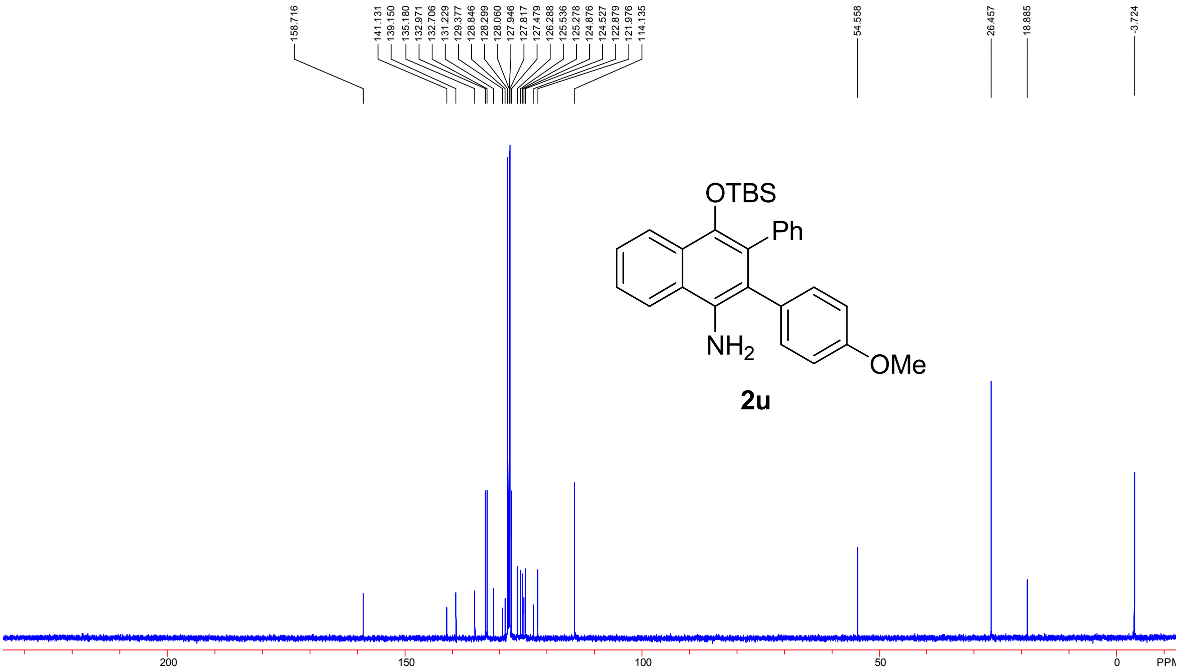

^{1}H NMR(400 MHz, C_6D_6, Me_4Si)

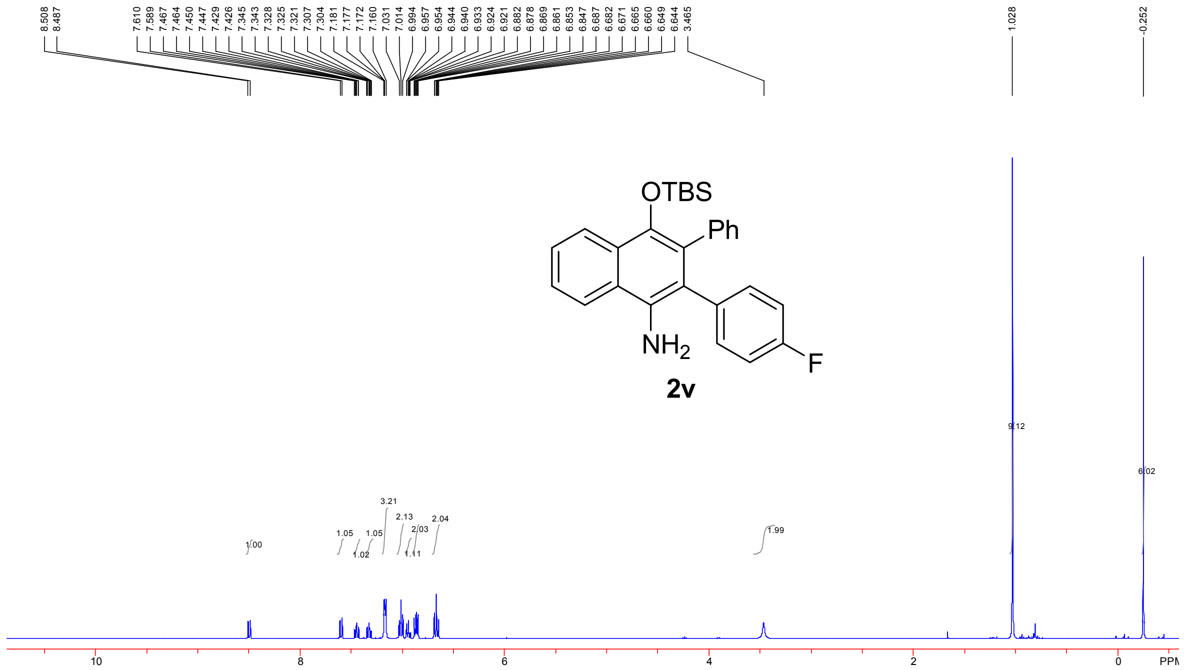

^{13}C NMR(100 MHz, C_6D_6, Me_4Si)

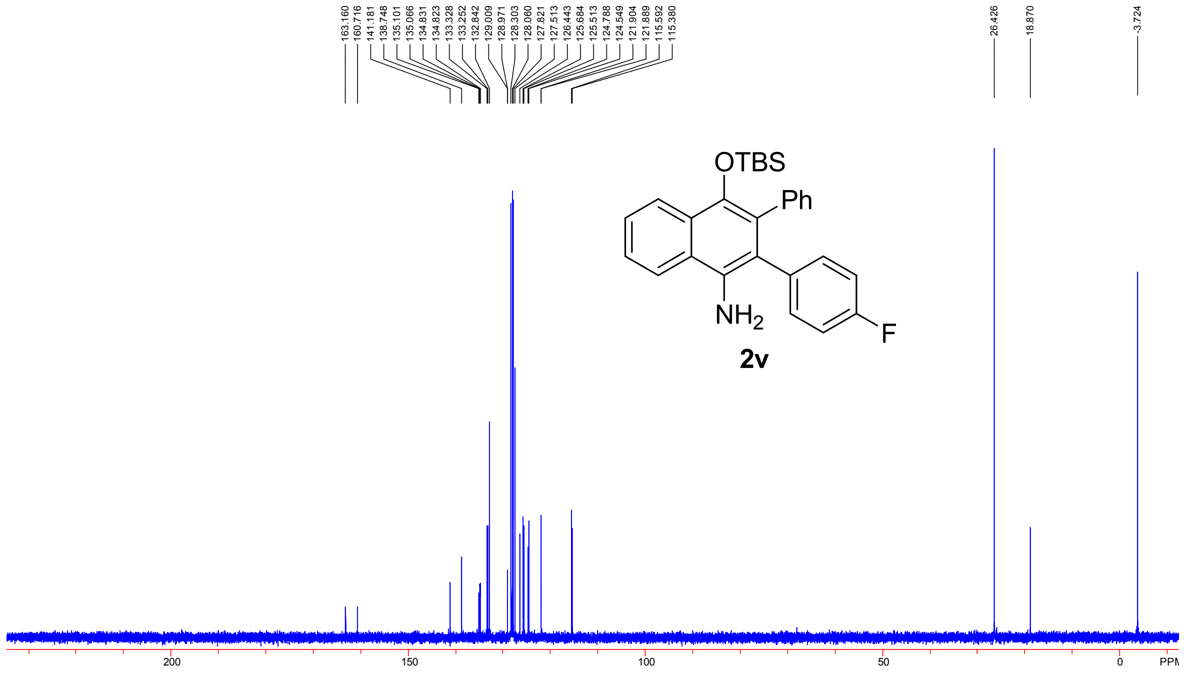

^{1}H NMR(400 MHz, C_6D_6, Me_4Si)

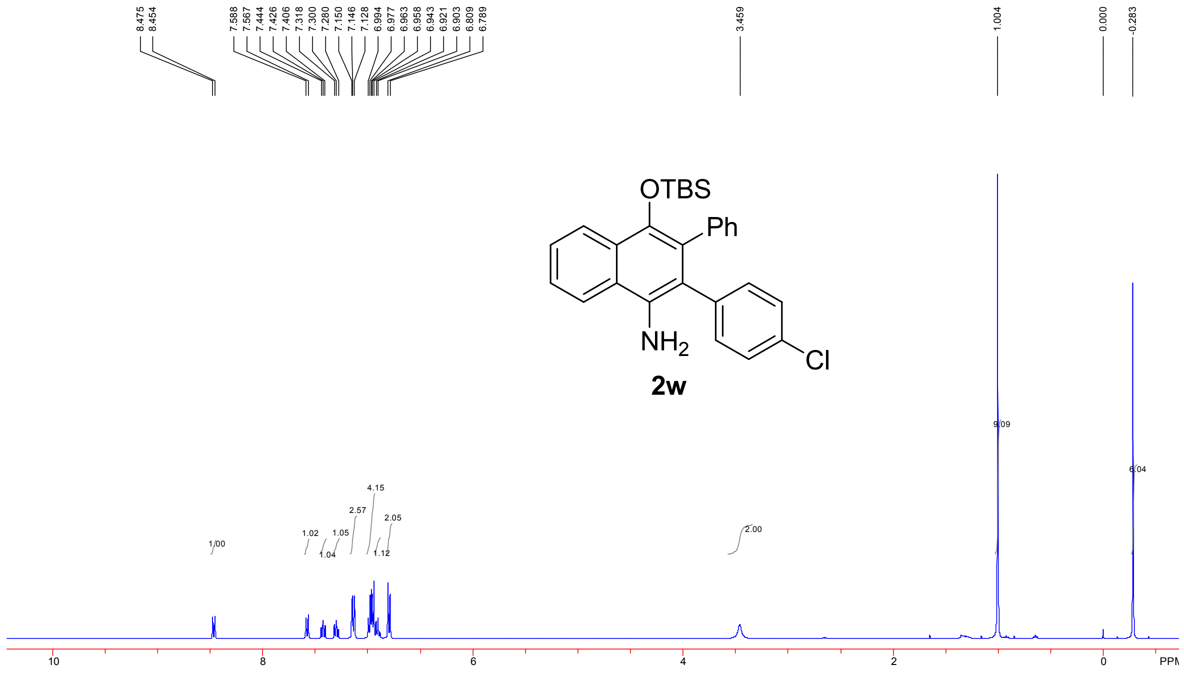

^{13}C NMR(100 MHz, C_6D_6, Me_4Si)

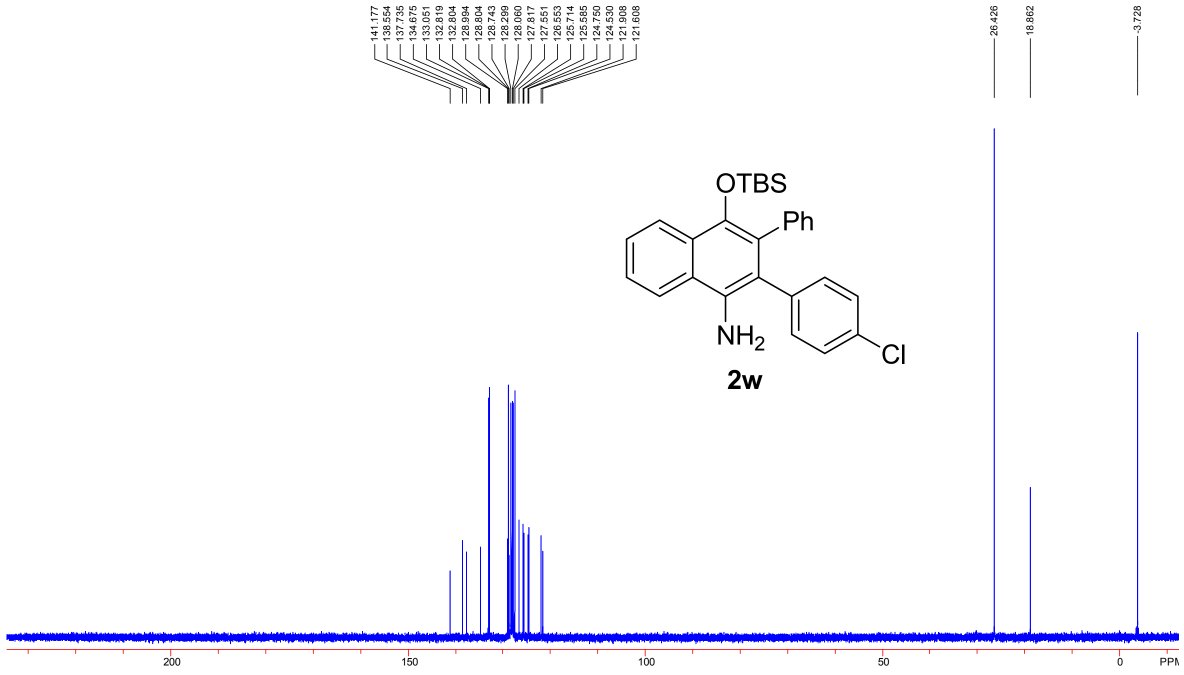

^{1}H NMR(400 MHz, C_6D_6, Me_4Si)

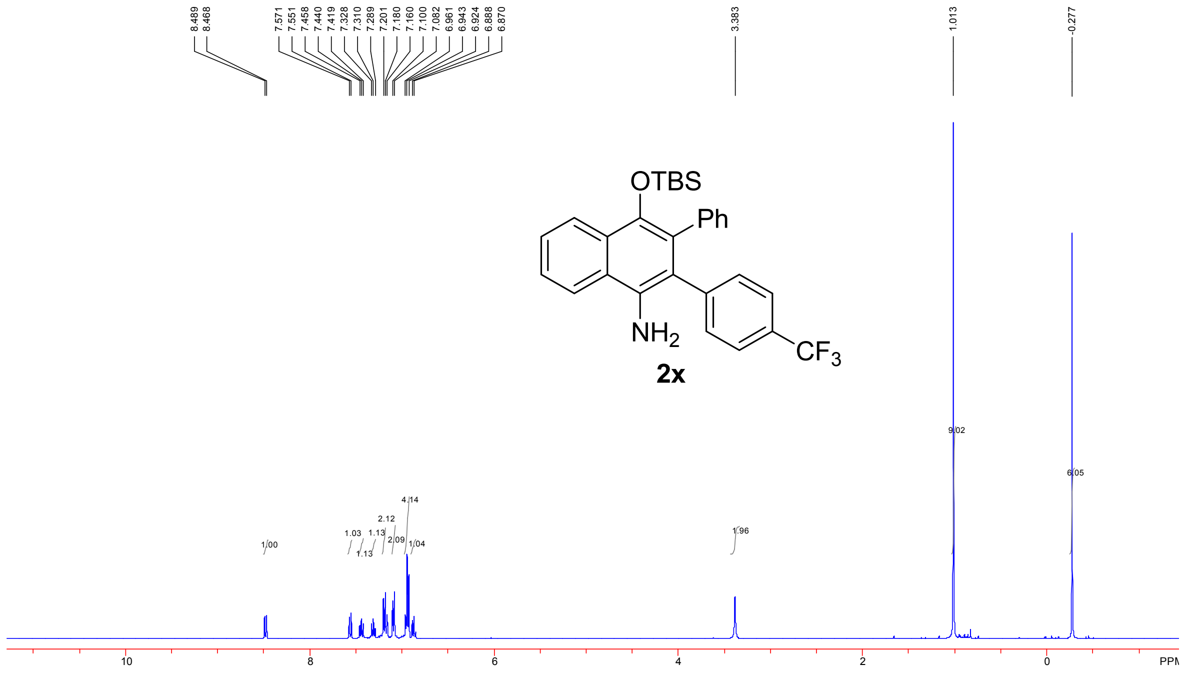

^{13}C NMR(100 MHz, C_6D_6, Me_4Si)

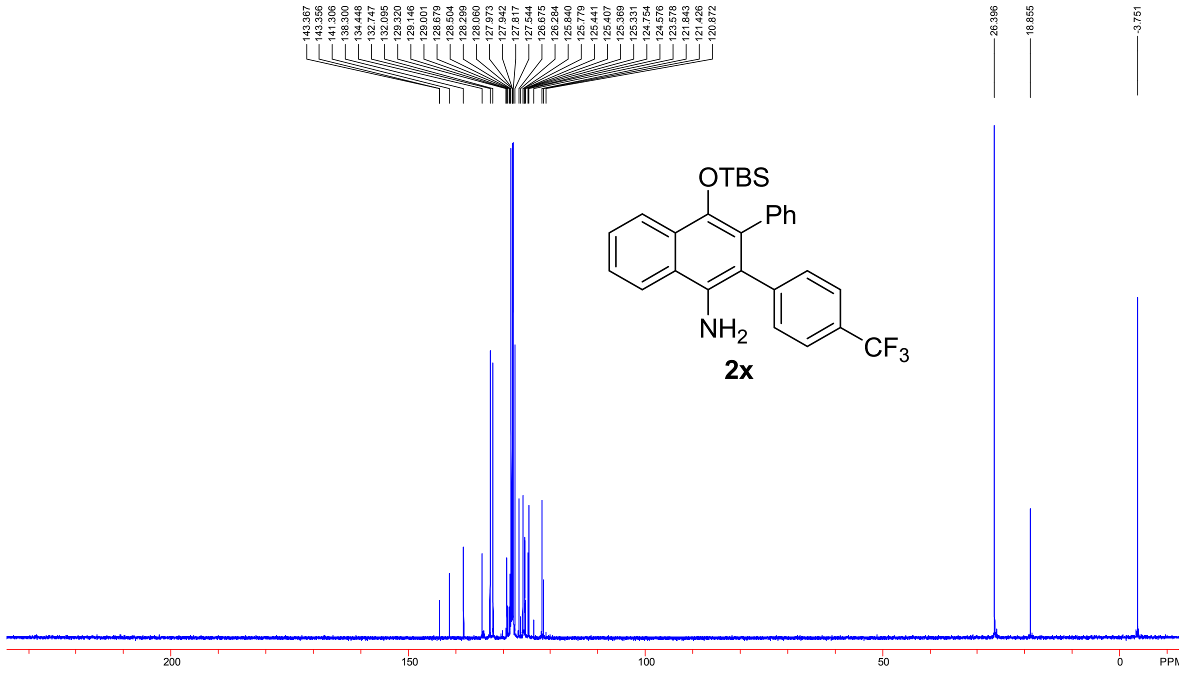

^{1}H NMR(400 MHz, C_6D_6, Me_4Si)

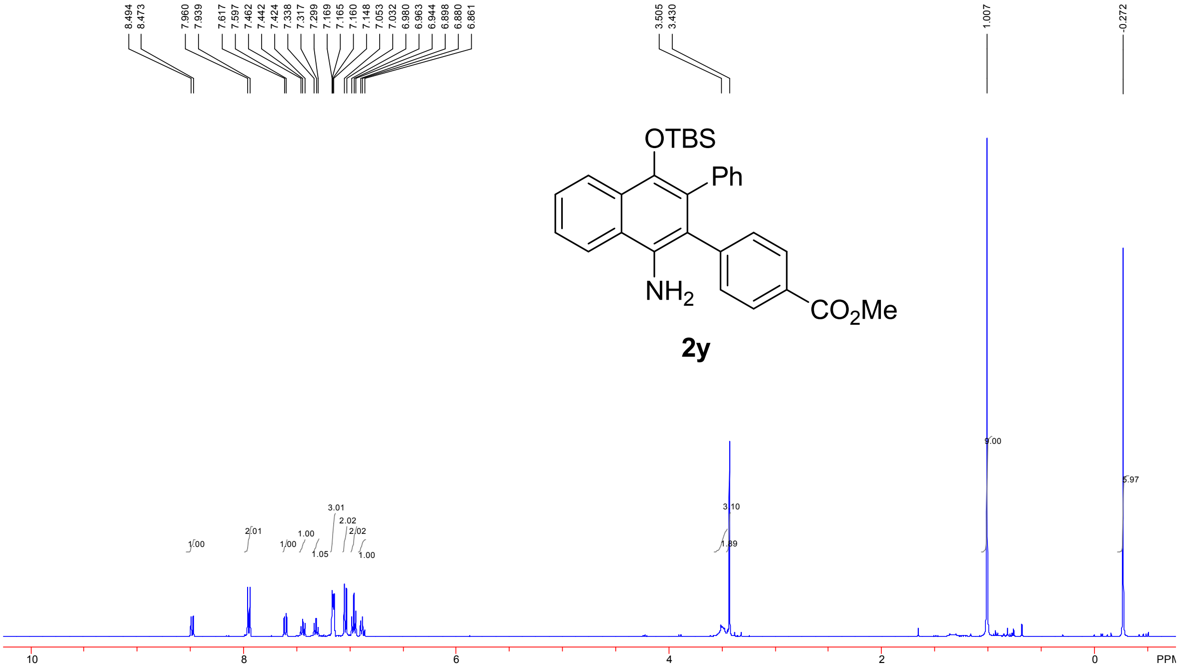

^{13}C NMR(100 MHz, C_6D_6, Me_4Si)

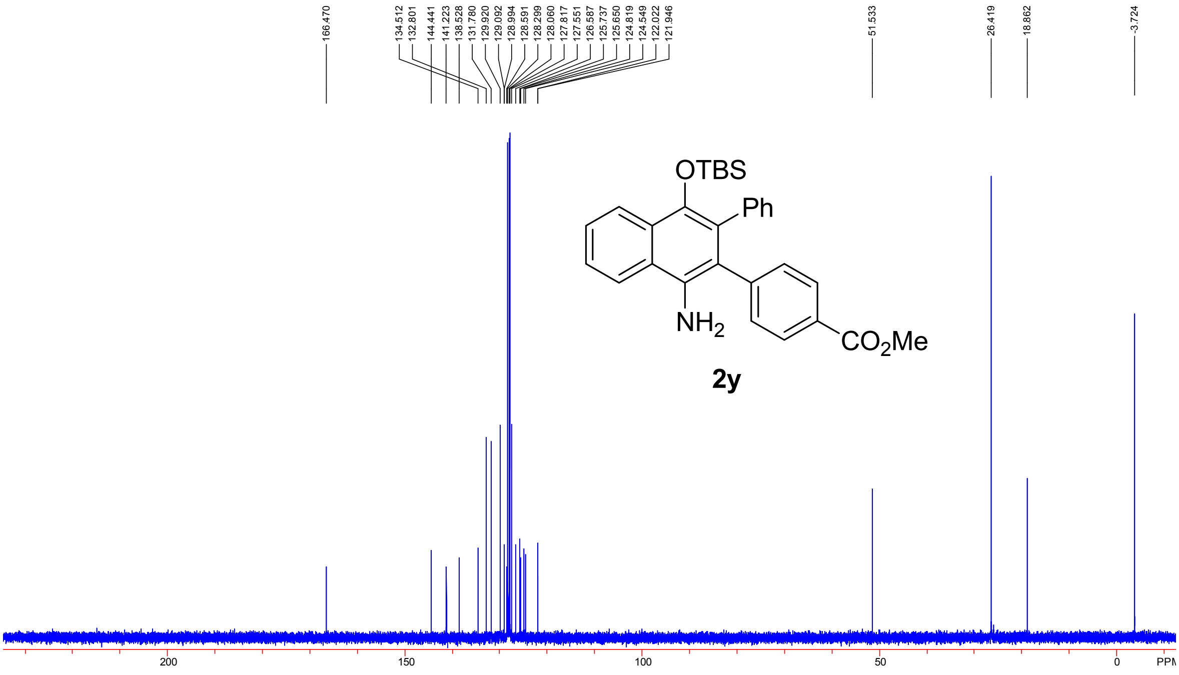

^{1}H NMR(400 MHz, C_6D_6, Me_4Si)

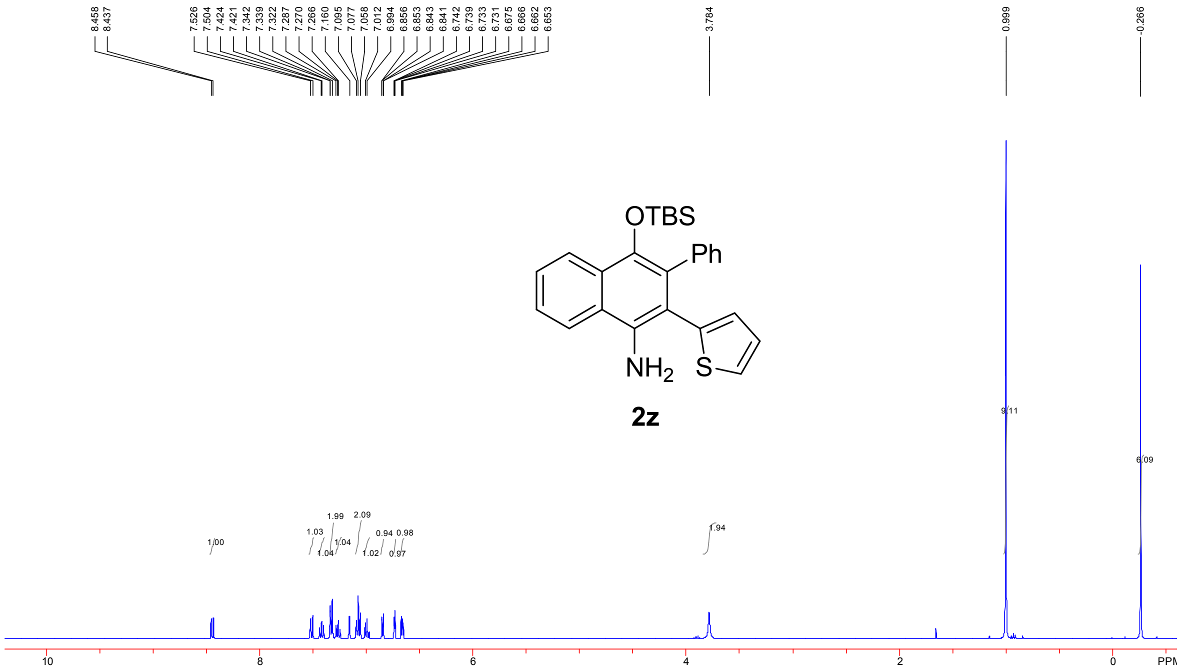

^{13}C NMR(100 MHz, C_6D_6, Me_4Si)

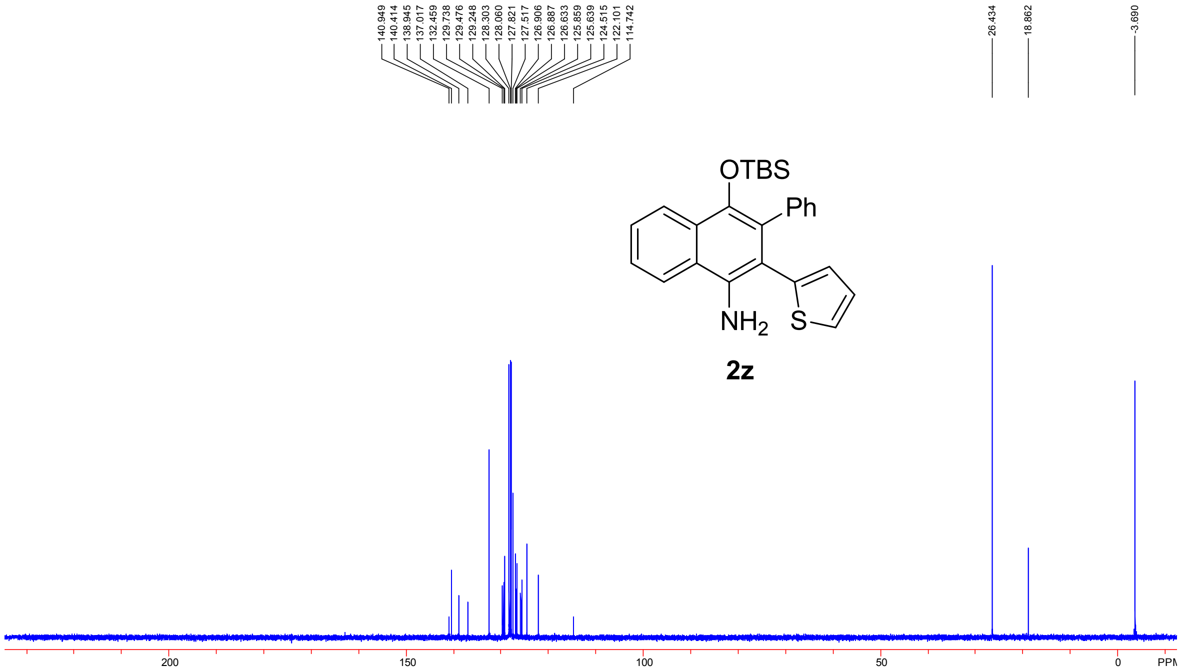

^{1}H NMR(400 MHz, C_6D_6, Me_4Si)

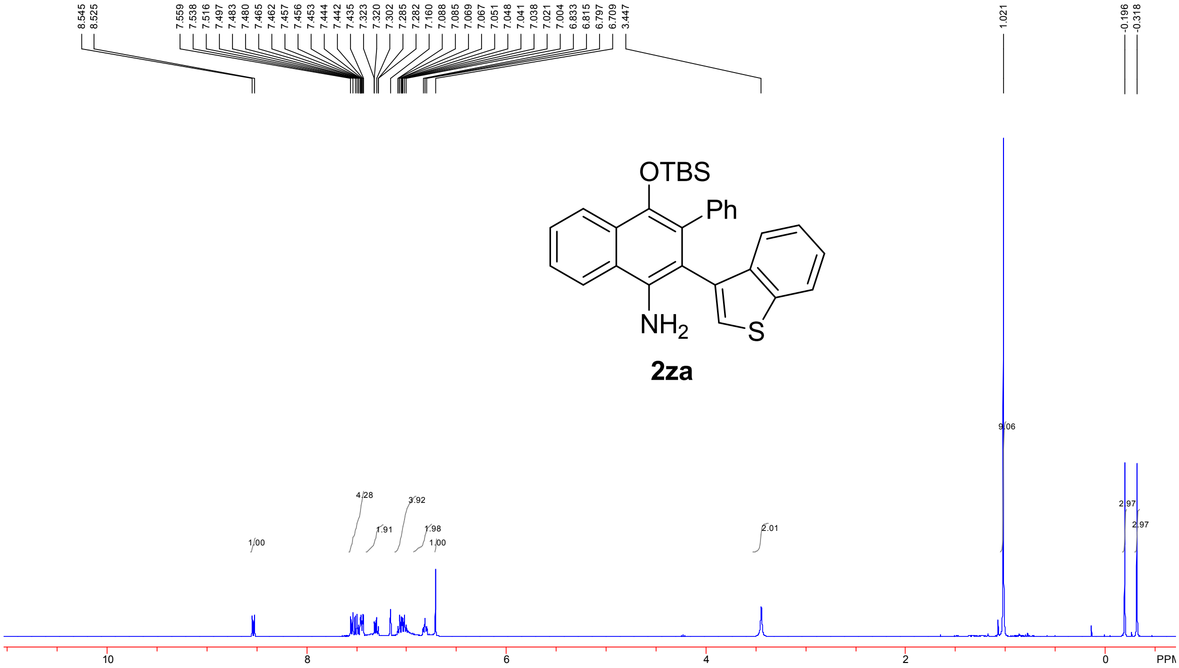

^{13}C NMR(100 MHz, C_6D_6, Me_4Si)

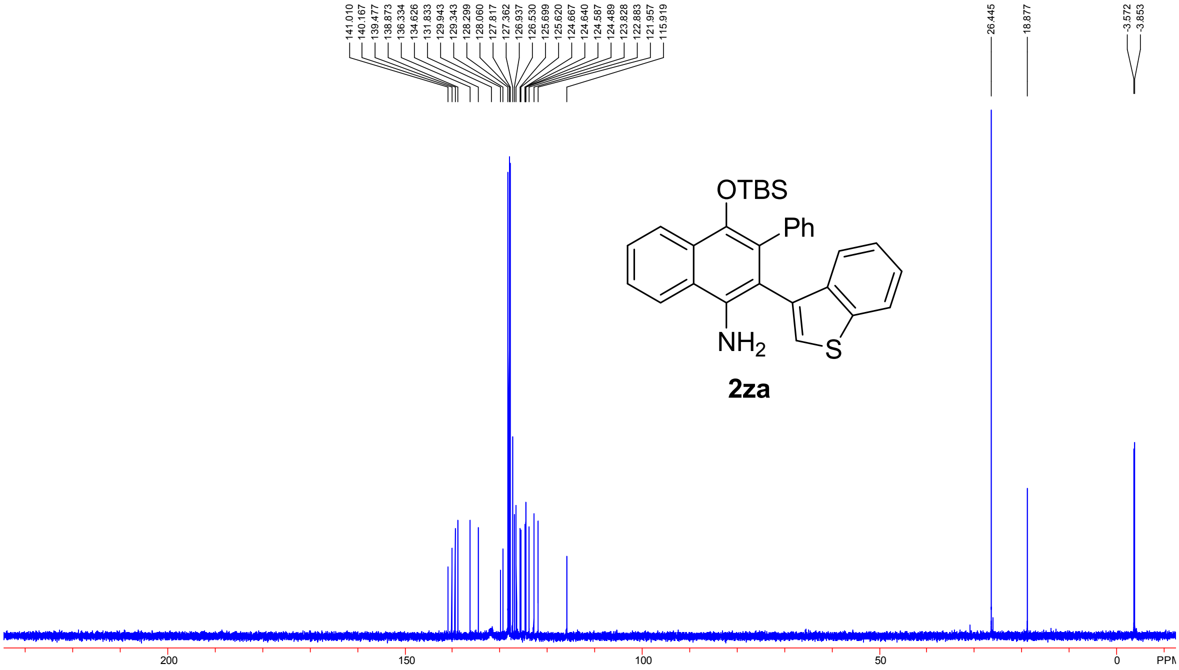

^{1}H NMR(400 MHz, C_6D_6, Me_4Si)

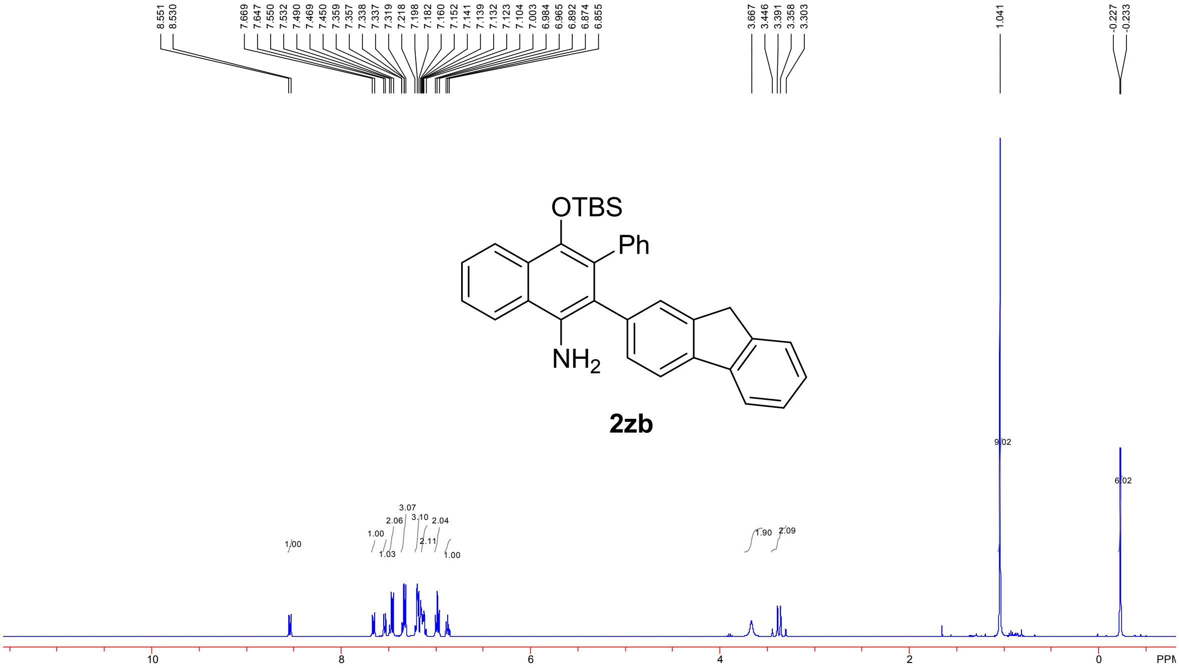

^{13}C NMR(100 MHz, C_6D_6, Me_4Si)

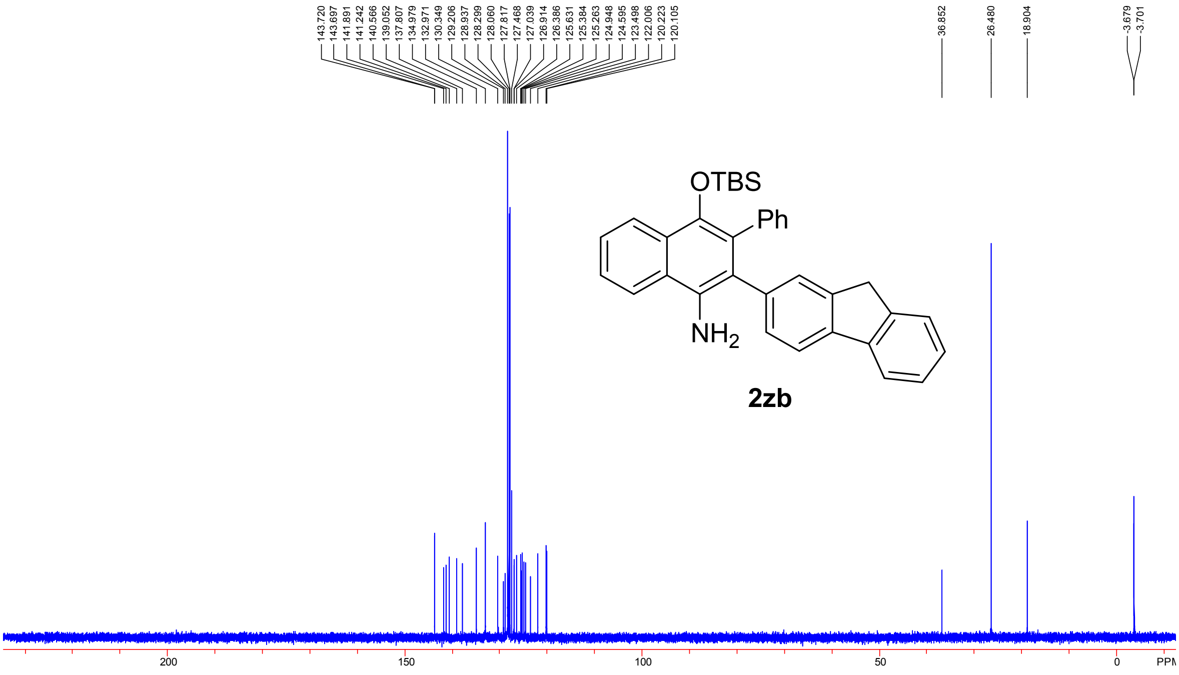

^{1}H NMR(400 MHz, C_6D_6, Me_4Si)

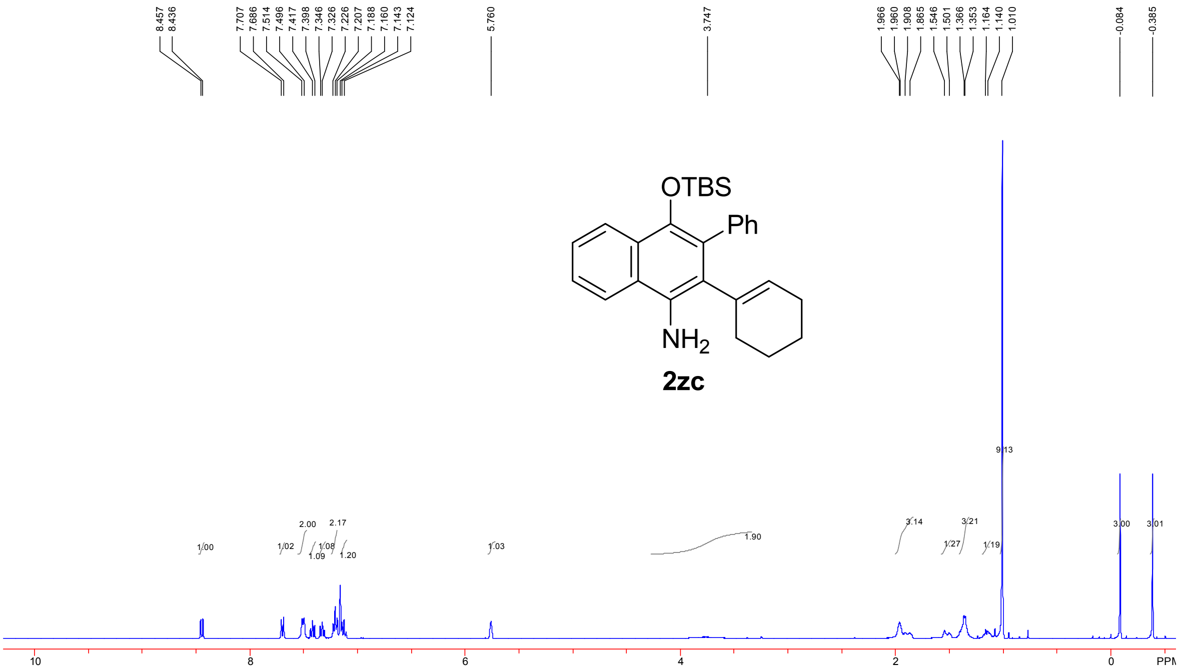

^{13}C NMR(100 MHz, C_6D_6, Me_4Si)

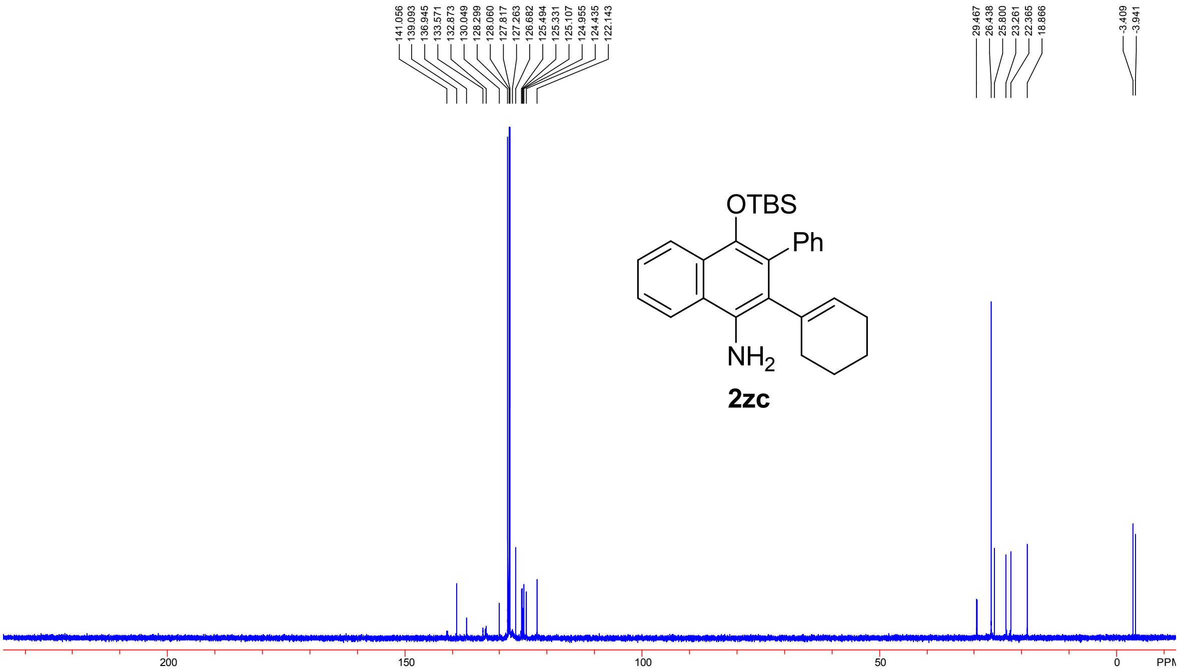

^{1}H NMR(400 MHz, C_6D_6, Me_4Si)

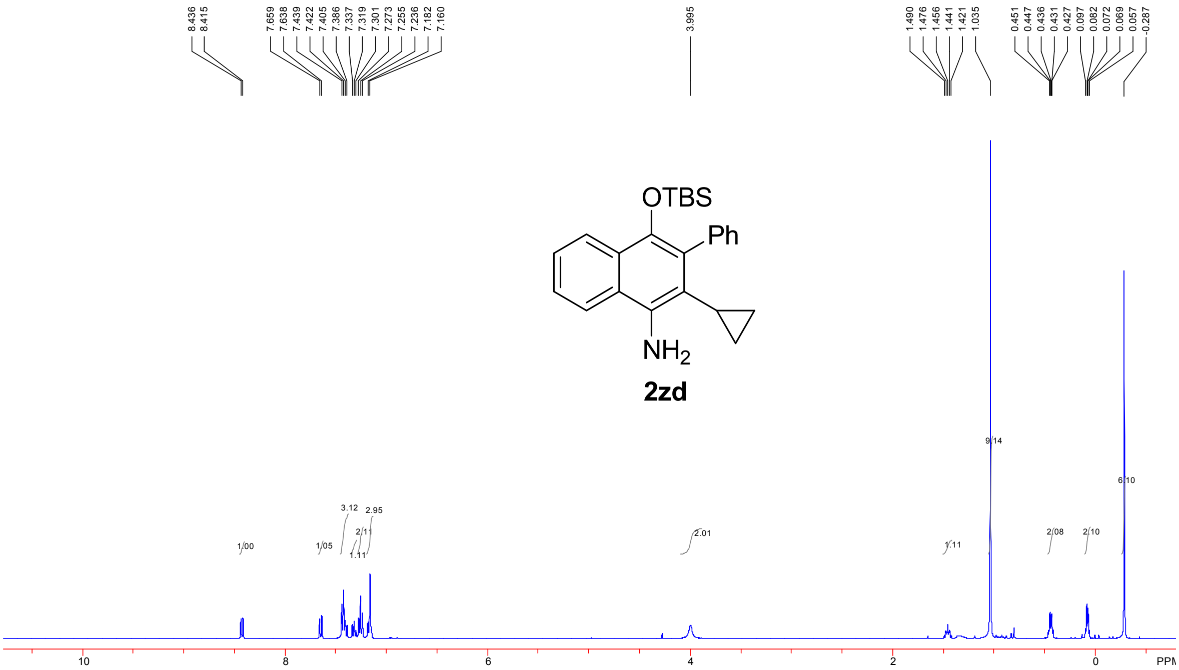

^{13}C NMR(100 MHz, C_6D_6, Me_4Si)

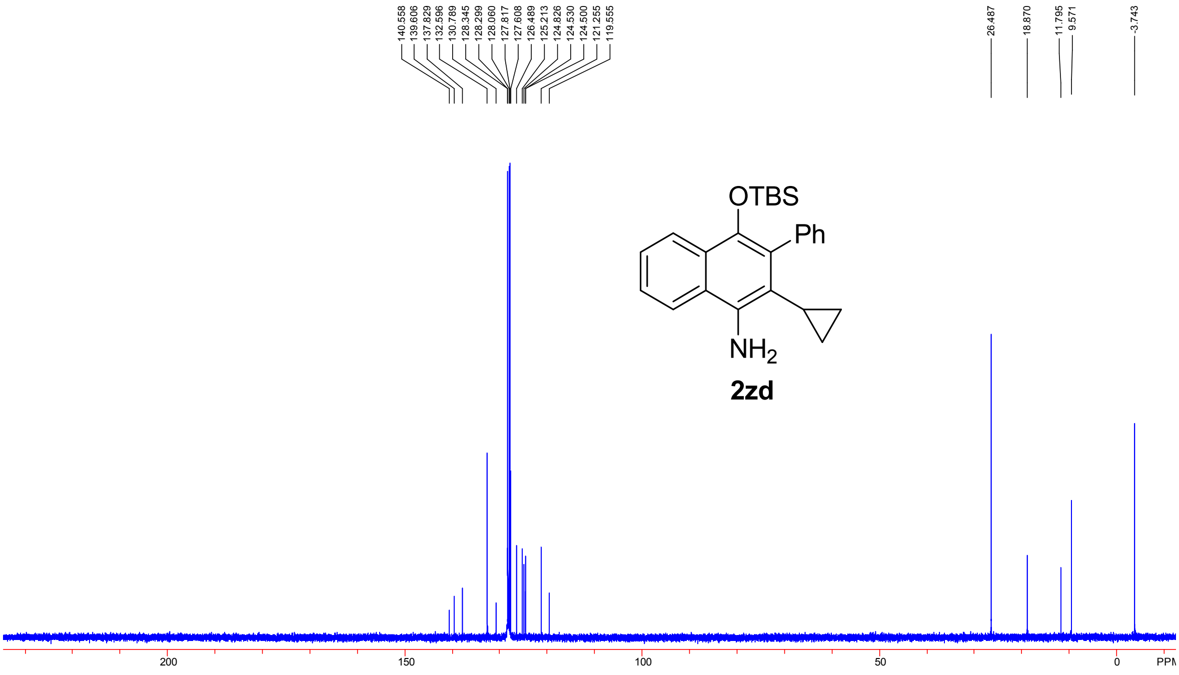

^{1}H NMR(400 MHz, C_6D_6, Me_4Si)

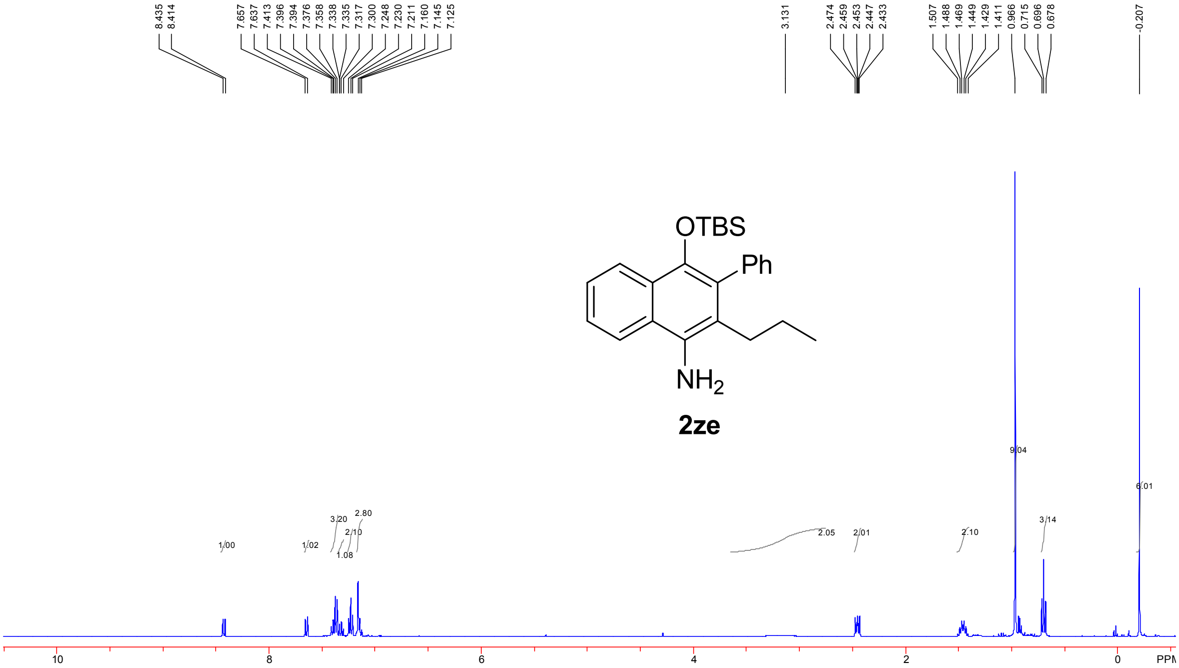

^{13}C NMR(100 MHz, C_6D_6, Me_4Si)

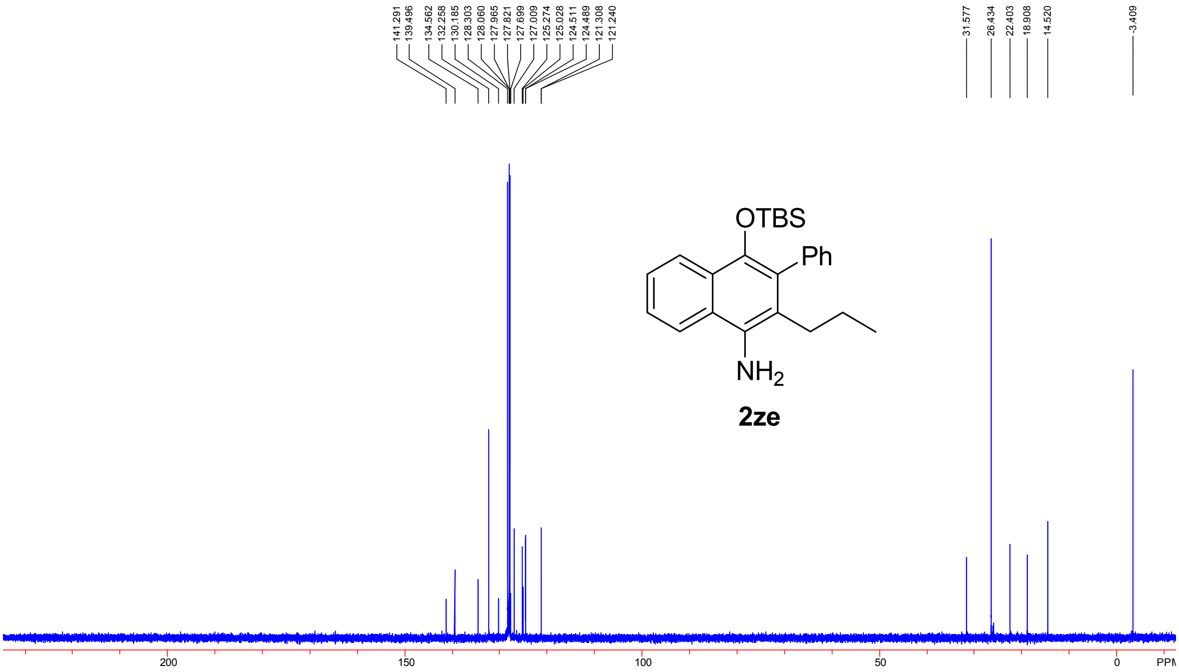

^{1}H NMR(400 MHz, C_6D_6, Me_4Si, -20-15 ppm)

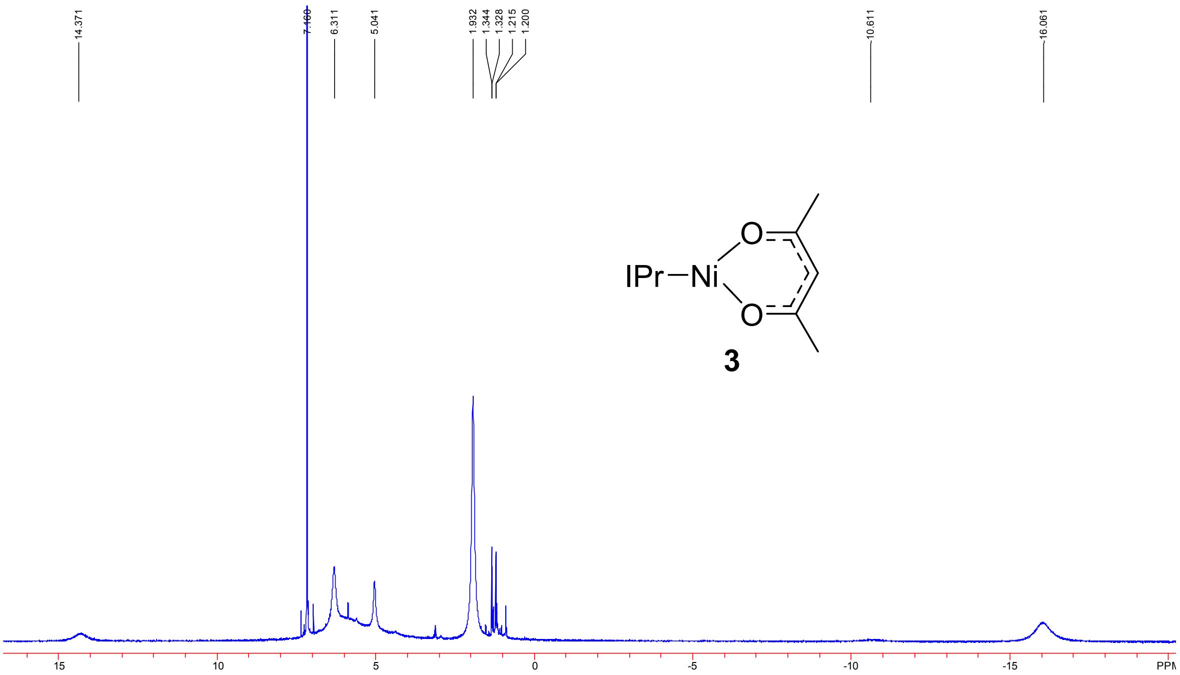

^{1}H NMR(400 MHz, C_6D_6, Me_4Si, 0-10 ppm)

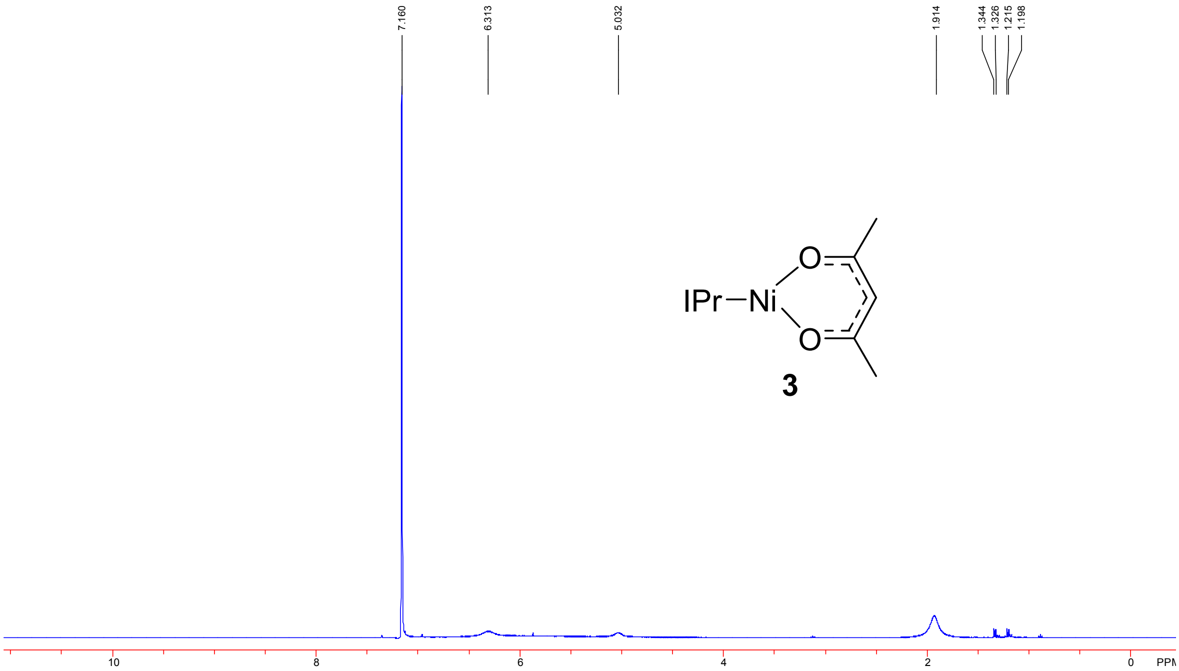

^{1}H NMR(400 MHz, $CDCl_3$, Me_4Si)

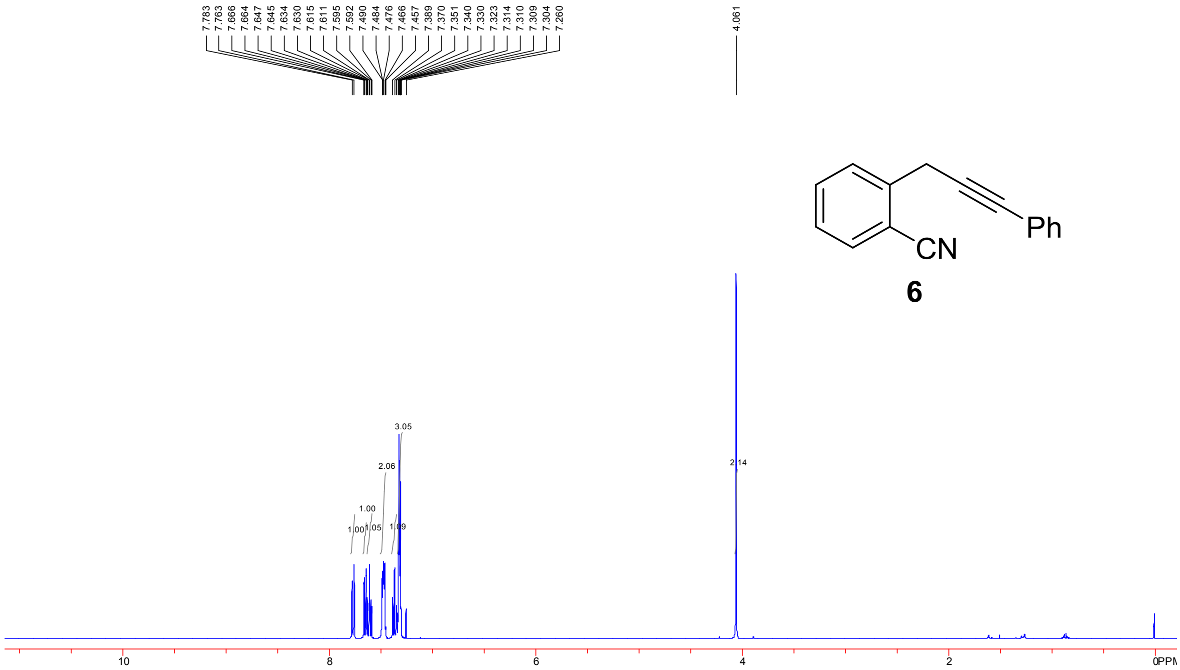

^{13}C NMR(100 MHz, $CDCl_3$, Me_4Si)

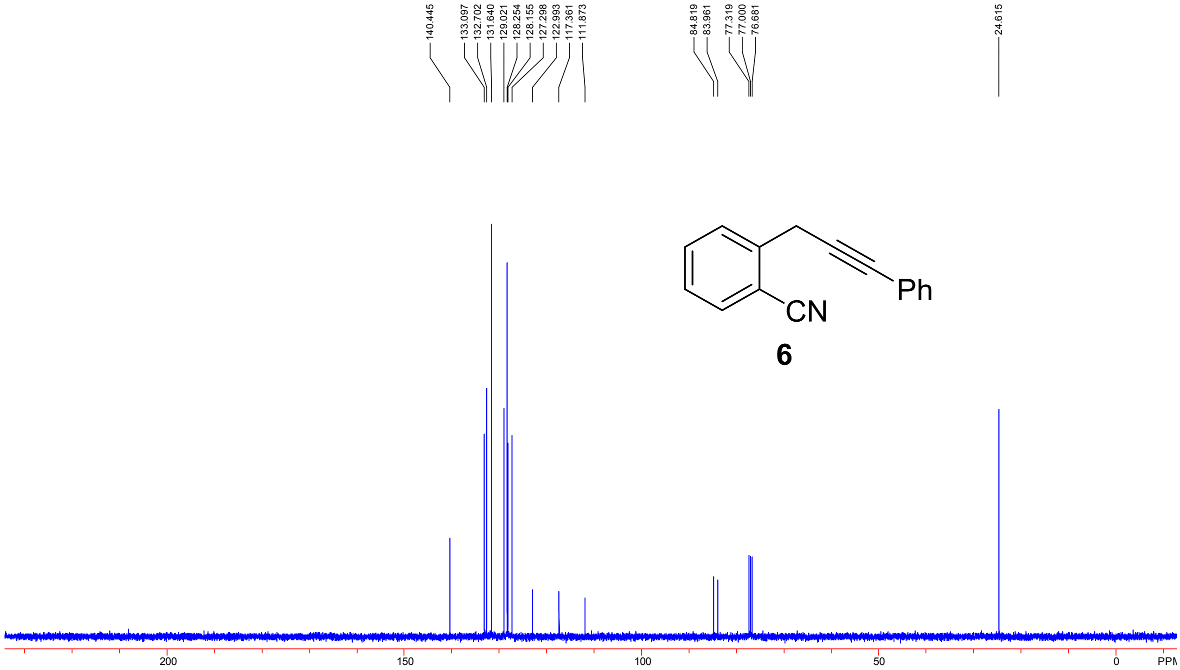

^1H NMR(400 MHz, C_6D_6, Me_4Si)

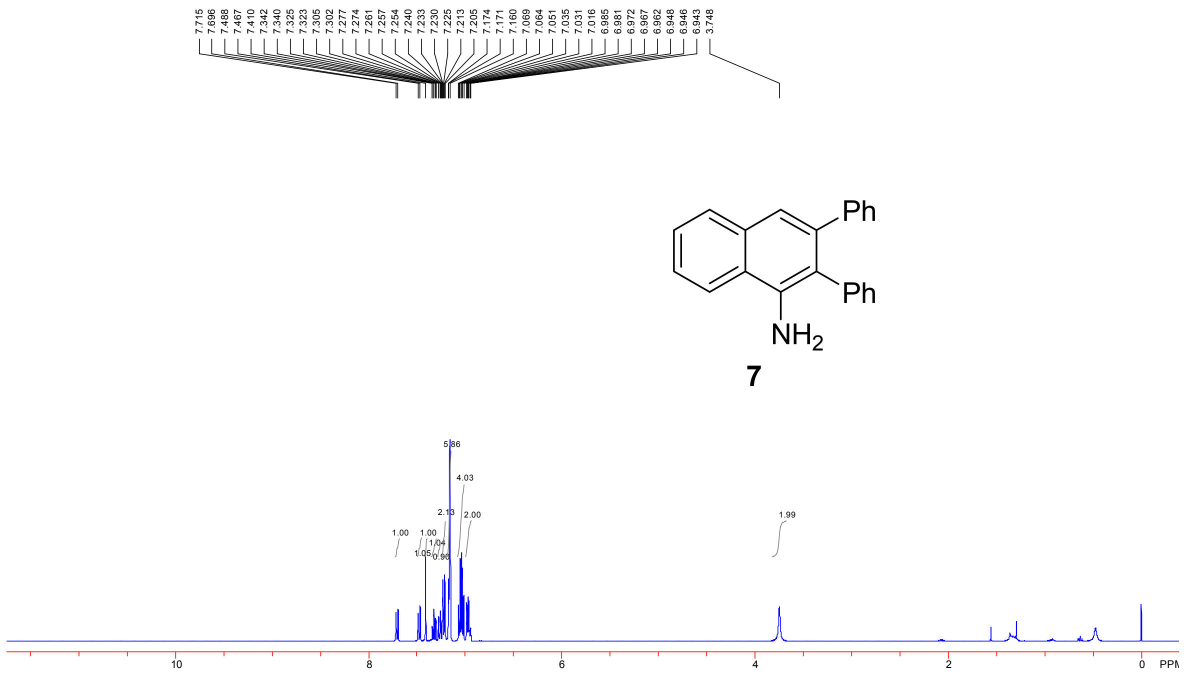

^{13}C NMR(100 MHz, C_6D_6, Me_4Si)

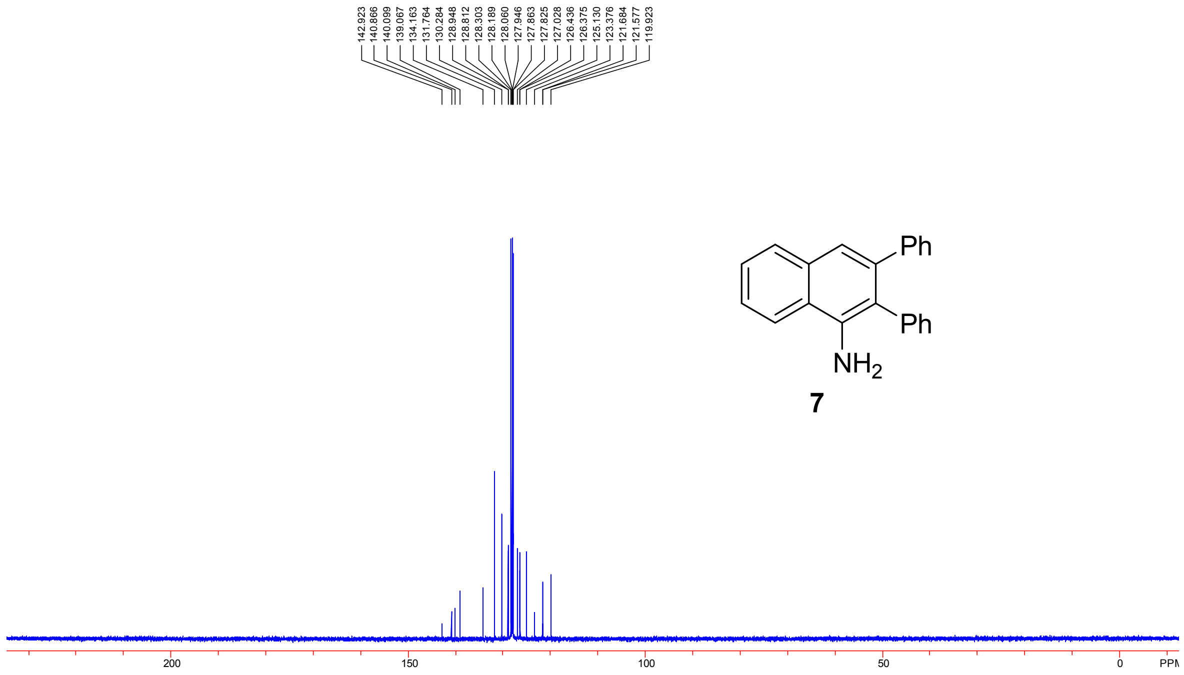

^{1}H NMR(400 MHz, C_6D_6, Me_4Si)

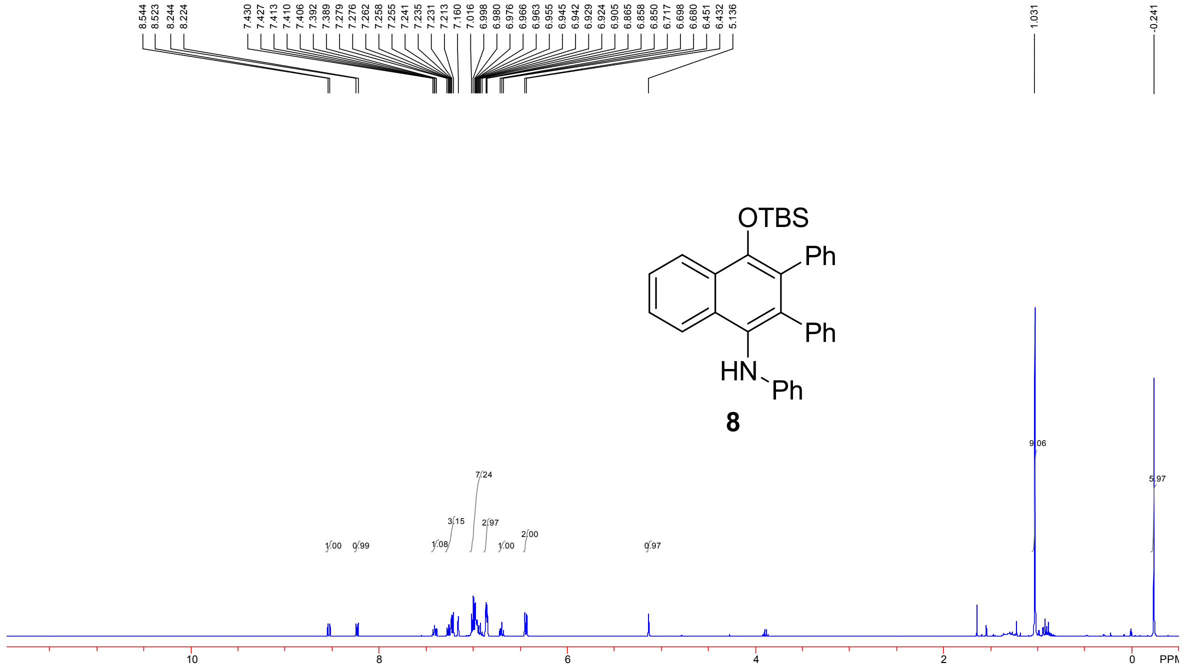

^{13}C NMR(100 MHz, C_6D_6, Me_4Si)

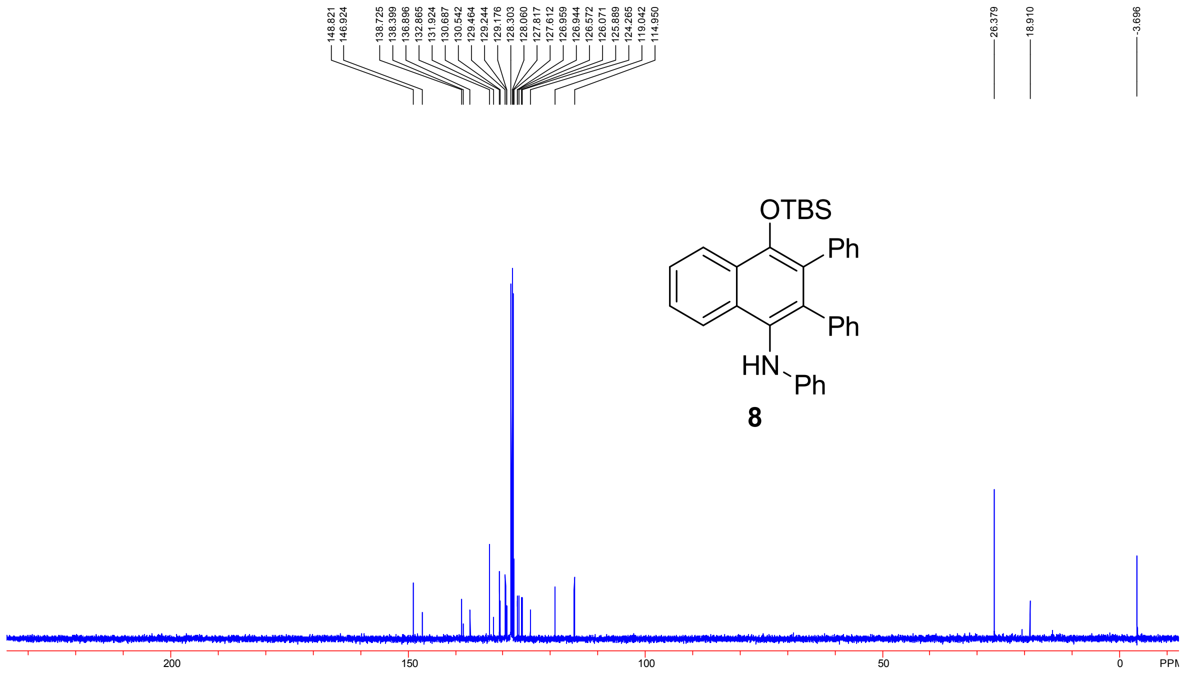

Supplement: Supplementary file 1 [file SC-007-C6SC01191H-s001.pdf]
